# International expert consensus on micronutrient supplement use during the early life course

**DOI:** 10.1186/s12884-024-07123-5

**Published:** 2025-01-20

**Authors:** Irene Cetin, Roland Devlieger, Erika Isolauri, Rima Obeid, Francesca Parisi, Stefan Pilz, Lenie van Rossem, Maternal Nutrition Delphi Study Group, Régine Steegers-Theunissen

**Affiliations:** 1 Department of Women, Mother and Neonate, “Vittore Buzzi” Children’s Hospital, University of Milan, Milan, Italy; 2 Department of Obstetrics and Gynaecology, University Hospitals Leuven, Leuven, Belgium; 3 Department of Obstetrics, Gynaecology and Fertility, GZA campus Sint-Augustinus, Wilrijk, Belgium; 4 Department of Pediatrics, University of Turku, Turku University Hospital, Turku, Finland; 5 Department of Clinical Chemistry, Saarland University Hospital, Homburg, Germany; 6 Department of Endocrinology and Diabetology, Medical University of Graz, Graz, Austria; 7 Department of Obstetrics and Gynaecology, Erasmus MC, University Medical Center Rotterdam, Dr. Molewaterplein 40, Rotterdam, 3015 CE Netherlands; 8 Present Address: Department of BioMedical and Clinical Sciences, University of Milan and Fondazione IRCCS CA’ GRANDA, Ospedale Maggiore Policlinico, Milan, Italy; 9 Present Address: Division of Human Nutrition and Health, Wageningen University, Wageningen, Netherlands

**Keywords:** Maternal diet, Vitamins, Trace-elements, Preconception, Pregnancy, Lactation, Guidelines

## Abstract

**Background:**

Growing evidence demonstrates that maternal nutrition is crucial for the health of the mother-to-be, and early life course of the offspring. However, for most micronutrients, guidelines are inconsistent. This Delphi study aimed to investigate the level of expert consensus on maternal nutrition and micronutrient needs during preconception, pregnancy and lactation.

**Methods:**

We conducted a two-round web-based Delphi survey on various topics including general approaches to diet and supplement use, and existing guidelines. For the periods of preconception, pregnancy and lactation, questions focused on the importance and strength of evidence for supplement use with the following micronutrients for low- and high-risk populations: folic acid, choline, iodine, magnesium, calcium, iron, selenium, docosahexaenoic acid (DHA), and vitamins B1, B2, B6, B12, D and K.

**Results:**

Thirty-five experts participated in the panel, who were healthcare professionals (HCPs), researchers and joint HCP-researchers with expertise in nutrition, gynaecology and/or obstetrics. Panellists reached consensus on the importance of diet and dietary supplement use during pregnancy and agreed on the lack of clarity and consistency in current guidelines, and the need for education in these areas for HCPs, pregnant people and the general population. For general low-risk populations, there was consensus on the importance of supplement use with iron and vitamin D from preconception through lactation, with folic acid and iodine from preconception through the second and third trimesters, respectively, with DHA from the first trimester through lactation and with calcium during lactation. Panellists agreed that the evidence for supplement use with each of these micronutrients during these phases to improve outcomes and/or foetal development is strong, except for vitamin D (preconception), DHA (first trimester), and iron (both periods). There was also consensus that supplement use advice should be tailored for people following vegan/vegetarian diets, restricted diets due to food intolerances, obesity, polycystic ovary syndrome, diabetes mellitus, and previous nutrition-related pregnancy complications.

**Conclusion:**

The findings revealed robust consensus on various aspects of maternal nutrition, including the need for education, the lack of consistency in current guidelines on supplement use, the importance of supplement use across specific phases of pregnancy and the at-risk groups requiring tailored approaches.

**Supplementary Information:**

The online version contains supplementary material available at 10.1186/s12884-024-07123-5.

## Background

The preconception period and pregnancy are critical periods in the early life course of two generations, marked by significant physiological changes [1]. As the maternal body prepares for pregnancy and adapts to support foetal growth and development, the demand for certain nutrients increases [2]. Accumulating evidence suggests that nutrients, especially micronutrients, play a crucial role in maternal health throughout the reproductive period and beyond [2]. Deficiencies can impact fertility, the course and outcome of pregnancy, and the lifelong health of both the mother and child [2–6].

Although certain nutrients are widely recognised and incorporated into guidelines for intake and/or supplement use, debate continues around their significance at different pregnancy stages, while others are less frequently highlighted [2, 7, 8].

Folate (vitamin B9) is a recognised micronutrient before and during the first trimester of pregnancy [9], as folic acid has been found to reduce the risk of neural tube defects (NTDs) and potentially other adverse outcomes [10]. However, the benefits of continuing folic acid supplement use later in pregnancy, the use of high dosages, and its long-term health impacts on mother and child are not fully clear [11].

Iron is another commonly acknowledged micronutrient for which demands increase during pregnancy [4, 12, 13]. Despite its recognised role in foetal development and maternal haematopoiesis, concerns around potential iron overload have resulted in varied recommendations [4, 12, 14]. Several studies support the role of iodine in foetal thyroid and neural development [15], and the significance of vitamin D for bone health and various pregnancy outcomes [16, 17]. The essential nutrient choline is crucial for foetal health and brain development. The evidence on the impact of sufficient choline intake during pregnancy and lactation on foetal and infant health is emerging. Growing evidence also suggests that higher maternal choline intake is associated with a lower risk of NTDs in the offspring [9, 18, 19]. This raises the question as to why choline is not commonly emphasised in dietary recommendations for these critical periods. Similarly, docosahexaenoic acid (DHA), which is associated with foetal neural development and a reduced risk of preterm birth (PTB) [20, 21], and calcium, which has been associated in some studies with foetal bone health and prevention of pre-eclampsia [9, 22], are not as widely recommended.

It is confusing that the recommendations for micronutrient supplement use vary between guidelines (summarised in Additional File 1). For example, for iron, the US Institute of Medicine (IOM) set the recommended dietary intake (RDI) at 27 mg/day during pregnancy [23], whereas the European Food Safety Authority (EFSA) and the Standing Advisory Committee on Nutrition (SACN) have the same RDI for pregnant and lactating women, and non-pregnant women of reproductive age (16 mg/day and 14.8 mg/day, respectively) [24]. In addition, whilst the World Health Organization (WHO) recommends a daily universal supplement with iron (30–60 mg) [25], routine iron supplement use is not recommended in certain countries, such as the UK and Italy [26, 27]. For iodine, the EFSA has set the adequate intake at 200 µg/day during pregnancy and lactation compared with 150 µg/day for adult women who are not pregnant or breastfeeding. The American Thyroid Association and Australia and New Zealand national guidelines recommend that women who are planning a pregnancy, or who are currently pregnant or breastfeeding, should have a daily oral iodine supplement containing 150–200 µg of iodine [28, 29], and the German national consensus and German Federal Institute of Risk Assessment (BfR) recommend that pregnant and breastfeeding women should use a supplement with 100–150 µg/day [30, 31], whereas the WHO and United Nations Children’s Fund (UNICEF) recommend universal salt iodisation to improve iodine status [32]. For vitamin D, recommendations during pregnancy vary considerably. For example, the IOM and the EFSA have an RDI of 600 international units (IU)/day (15 µg/day) [33, 34], D–A–CH (Germany, Austria, Switzerland) nutrition societies recommend 800 IU/day (20 µg/day) [35], and the Endocrine Society in the US and Central European expert guidelines recommend 1500–2000 IU/day (37.5–50 µg/day) [36, 37].

Beyond recommendations for specific micronutrients, there are broader questions surrounding overall approaches to diet and supplement use during pregnancy. Factors such as individual needs, pre-existing health conditions and lifestyle choices can influence nutritional requirements and are frequently debated [6].

Given the recognised importance of maternal nutrition, combined with the ongoing debates and heterogeneous recommendations, there is a need to explore expert opinions regarding knowledge gaps, unmet scientific and educational needs, and state-of-the-art knowledge on maternal nutrition. The Delphi method provides a robust approach for determining consensus among experts [38], especially in situations where there is limited evidence and expert opinion is valuable [39]. Through iterative surveys, the Delphi method can be used to gather and refine expert perspectives, working to build consensus and identify common areas of agreement and disagreement. The insights gained can then help inform healthcare professionals (HCPs) and target groups, assist in implementation in clinical practice, and be used for future research.

The aim of this study was, therefore, to investigate the level of expert consensus on optimal maternal nutrition during the early life course of the offspring, covering the critical preconception, pregnancy and lactation periods, using a modified Delphi method.

## Methods

### Study design

**Fig. 1:**
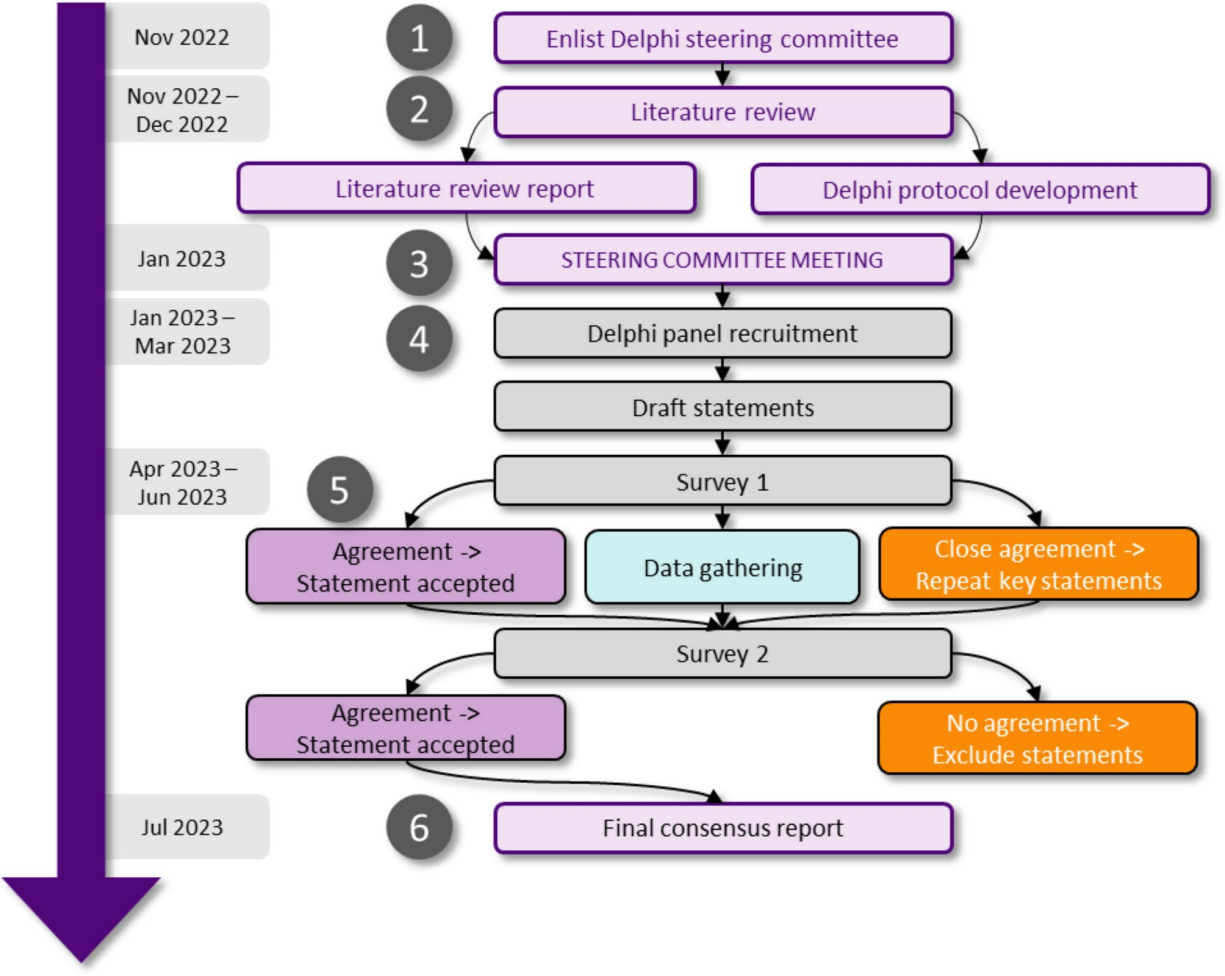
Overview of modified Delphi process

The study employed a modified Delphi process (Fig. 1), consisting of two iterative survey rounds, based on published standards of Delphi methodology [38, 39]. First, the lead and corresponding authors (IC and RST), serving as co-chairs, identified experts to form a steering committee (SC). The SC consisted of eight clinicians and/or researchers specialising in gynaecology and obstetrics (IC, RD, FP), periconception epidemiology and medicine (RST), paediatrics (EI), nutrition (RO, LVR) and endocrinology (SP). To inform survey development and provide structure to SC discussions, a scoping review was conducted on 31 October and 1 November 2022, focusing on maternal nutrition, including diet and selected micronutrient needs during preconception and pregnancy, as well as existing guidelines for their intake and supplement use. We restricted the search to human studies that had been published in the last 5–10 years and in the English language. Details of the search strategy are included in the Supplementary Methods (Additional File 2). A total of 82 publications were reviewed in depth. Based on subsequent discussions of the literature review findings, the SC prioritised topics for inclusion in the Delphi surveys, developed survey questions and determined the panel recruitment method along with the definition of consensus.

### Panel recruitment

The corresponding and first authors of key articles identified in the literature review were invited to participate as expert panellists; 140 authors were invited to take part. No additional inclusion criteria were defined. Prior to participating, prospective panellists were informed of the survey topics and approximate survey length and told that their responses would be kept anonymous. Panellists did not receive reimbursement for their time. The SC were not panel members and did not complete the Delphi surveys. The study was reviewed by Pearl Institutional Review Board (2023-0047, https://www.pearlirb.com/) and received a Category 2 exemption from full review, in accordance with the US Department of Health and Human Services and Code of Federal Regulations (CFR; 45 CFR 46.104(d)(2)).

### Delphi surveys

A two-round web-based survey was conducted between April and June 2023 using SurveyMonkey^®^ (https://uk.surveymonkey.com/). All questions from both surveys are provided in the Supplementary Methods (Additional File 2). The first survey collected basic demographic information and was then organised into three main sections. Section 1 included multiple general questions relating to educational needs, existing guidelines, and approaches to diet (for example, a Mediterranean diet [40]) and supplement use. Section 2 was structured to correspond with the key periods of preconception, pregnancy and lactation. For each period, questions focused on the importance of supplementing with the following micronutrients for general or low-risk populations: folic acid, choline, iodine, magnesium, calcium, iron, selenium, DHA, and vitamins B1, B2, B6, B12, D and K. This was followed by a general subsection with questions around supplement use of specific nutrients. Section 3 included multiple general questions around tailored approaches and nutrient needs in specific at-risk groups.

Throughout the surveys, panellists were asked to rate questions on a 7-point Likert scale. For each question, a ‘not applicable’ (NA) option was included, and panellists were asked to select this if they felt that a question was outside of their experience or area of expertise. They were also encouraged to provide comments/reasoning for their responses in free-text boxes. Panellists’ responses to the first survey were used to inform the focus of the second survey. In line with standard Delphi methodology [38, 39], which advocates for informed decision-making and result sharing, panellists received a summary of the first survey’s results prior to completing the second survey. For both surveys, panellists’ responses were kept anonymous and were equally weighted.

### Consensus definition and analysis

In line with other Delphi studies, consensus agreement/disagreement was defined a priori as ≥ 75% of panellists selecting 1 to 3 (agreement) or -1 to -3 (disagreement) for questions rated on a 7-point Likert scale [41]. ‘NA’ responses were considered invalid and excluded from the consensus calculation.

Questions on the importance of supplementing with specific nutrients that reached ‘close consensus’ in the first survey (defined as 70–74% of panellists selecting 1 to 3 or -1 to -3) were repeated in the second survey. To delve further into the rationale behind panellists’ rating of importance, the second survey introduced additional questions on the strength of evidence that supplement use with each of the examined nutrients improves outcomes. In instances where panellists rated the evidence as weak, subsequent questions asked them to specify whether this was due to a lack of evidence, discrepancies in existing evidence, existing evidence being of low quality or other rationale. The strength of consensus was investigated using the median and interquartile range (IQR) once consensus had been reached. Consensus was considered strong if the median was ≥ 2 or ≤-2 and the IQR ≤ 1. Where relevant, free-text responses were described in the results. An overview of free-text responses is available upon request.

## Results

In total, 140 experts were invited to participate in the panel. Of these, 35 (25%) participated in the first survey, with 32 completing and three partially completing the survey. Thirty-two (91%) panellists completed the second survey. Survey 1 ran from 28 March to 8 April 2023 and Survey 2 ran from 26 May to 13 June 2023. The panel (Maternal Nutrition Delphi Study Group) comprised 10 HCPs, 11 researchers and 14 joint HCP-researchers. Panellists were experienced in diverse clinical specialties, including gynaecology and obstetrics and nutrition. Demographic characteristics of the panellists are detailed in Table 1. Results from both surveys are provided in the Supplementary Results (Additional File 3).

**Table 1:** Demographic characteristics of panellists

Number of panellists	35
Continent	
Asia	2 (5.71%)
Australia	1 (2.86%)
Europe	25 (71.43%)
North America	6 (17.14%)
South America	1 (2.86%)
Current occupation	
Researcher/academic	11 (31.43%)
HCP	10 (28.57%)
Joint HCP-researcher/academic	14 (40.00%)
Clinical specialty	
Obstetrician/gynaecologist	4 (11.43%)
Nutritionist/dietician	10 (28.57%)
Paediatrician	4 (11.43%)
Endocrinologist	2 (5.71%)
Midwife	1 (2.86%)
Cardiologist	1 (2.86%)
Pharmacologist	1 (2.86%)
Biochemist	1 (2.86%)
Experience in current occupation	
≤ 5 years	0 (0%)
> 5–≤10 years	6 (17.14%)
> 10–≤15 years	8 (22.68%)
> 15–≤20 years	6 (17.14%)
> 20–≤25 years	6 (17.14%)
> 25 years	9 (25.71%)

HCP, healthcare professional

### General considerations around maternal nutrition

Panellists reached consensus on various aspects of maternal nutrition, encompassing educational needs, general approaches to diet and dietary supplement use, and perspectives on current guidelines (Additional File 3: Supplementary Results Table S1). There was strong consensus agreement on the importance of HCPs providing nutritional and supplement advice from preconception and throughout lactation (combined estimates median = 2–3, IQR = 1, 97–100% agreement), as well as on the need for increased education and awareness in these areas for HCPs, pregnant people and the public (combined estimates median = 3, IQR = 1, 94–97% agreement).

Panellists strongly agreed on providing advice to follow a Mediterranean diet and regular national dietary guidelines (combined estimates median = 2, IQR = 1, 77–84% agreement).

There was no consensus on the Dietary Approaches to Stop Hypertension diet or that specific diets should not be advised. Both general multivitamin/multi-micronutrient supplement use and tailored individualised approaches were considered important (combined estimates median 2–3, IQR = 1, 79–88% agreement). In free-text comments, panellists noted that although individualised strategies are optimal, they are resource intensive and may not be economically viable or more effective at enhancing health or preventing additional complications in general populations.

Regarding current guidelines, there was consensus and close consensus that these are not clear or harmonised across countries and organisations, respectively (combined estimates median=-1, IQR = 1–2, 73–75% agreement).

### Micronutrient supplement use during preconception, pregnancy and lactation in general low-risk populations

**Fig. 2:**
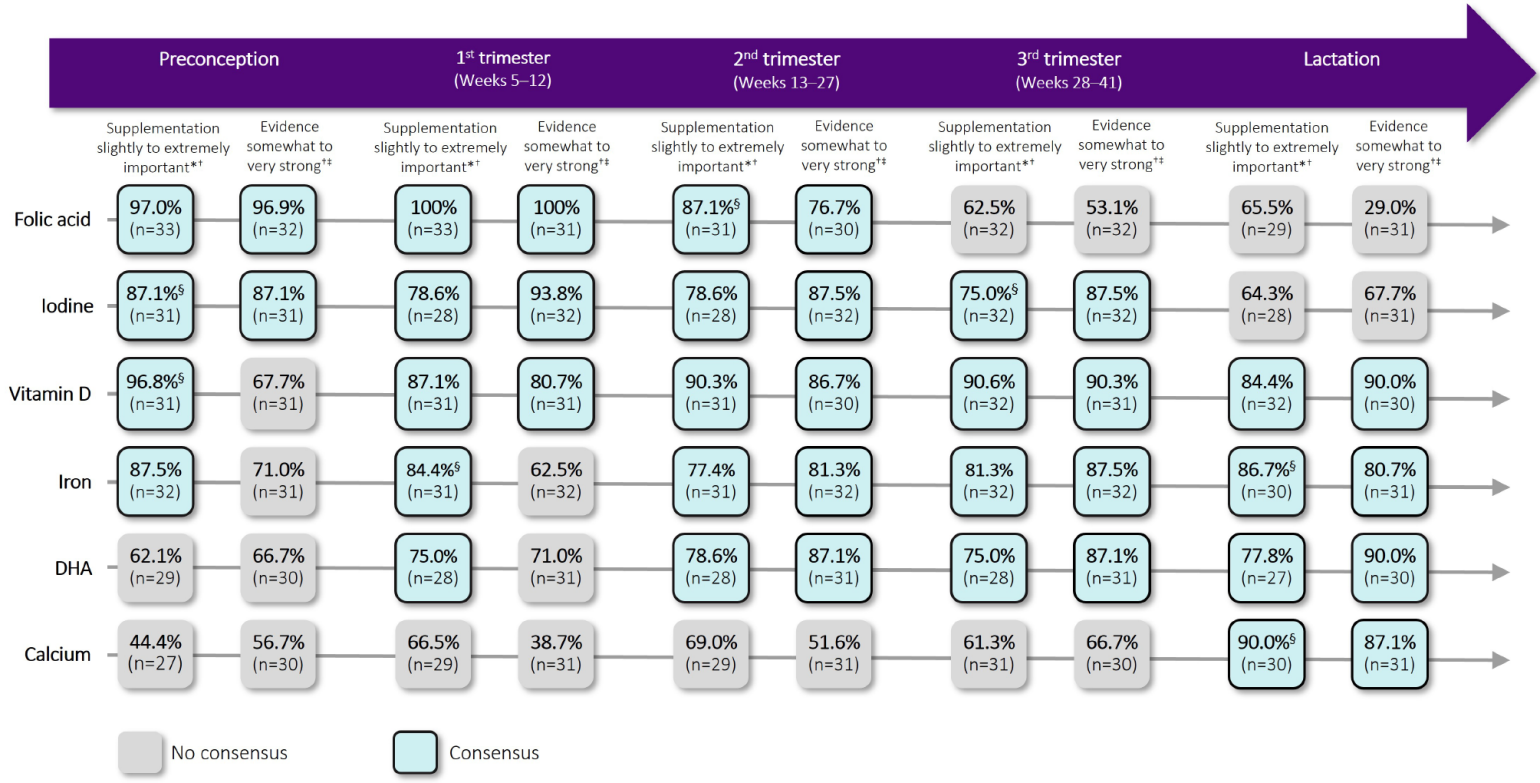
Micro- and macro-nutrients for which consensus was reached on supplement use. *Percentages represent the proportion of panellists selecting 1–3 on a 7-point Likert scale of importance for questions on the importance of supplement use. ^†^n refers to the number of valid responses; ‘NA/unsure’ responses were considered invalid and excluded. ^‡^Percentages represent the proportion of panellists selecting 1–3 on a 7-point Likert scale of strength for questions on the strength of evidence that supplement use improves outcomes and/or foetal development. ^§^Based on data from Survey 2; questions reaching close consensus in Survey 1 were repeated in Survey 2. DHA, docosahexaenoic acid

**Fig. 3:**
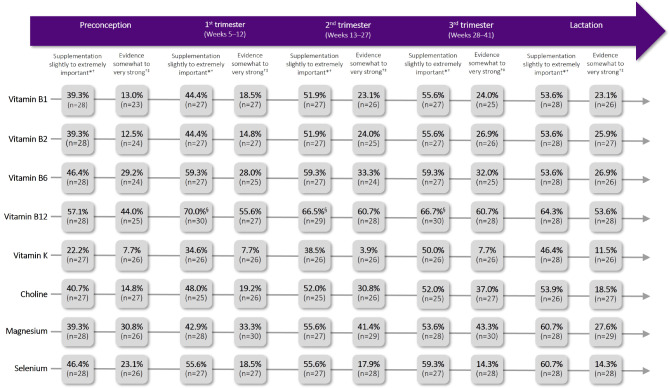
Micro- and macro-nutrients for which consensus was not reached on supplement use. *Percentages represent the proportion of panellists selecting 1–3 on a 7-point Likert scale of importance for questions on the importance of supplement use. ^†^n refers to the number of valid responses; ‘NA/unsure’ responses were considered invalid and excluded. ^‡^Percentages represent the proportion of panellists selecting 1–3 on a 7-point Likert scale of strength for questions on the strength of evidence that supplement use improves outcomes and/or foetal development. ^§^Based on data from Survey 2; questions reaching close consensus in Survey 1 were repeated in Survey 2

Panellists reached consensus on the importance of supplement use, and on the strength of evidence that supplement use improves outcomes and/or foetal development, for some key micronutrients across different stages of pregnancy (Figs. 2 and 3; Additional File 3: Supplementary Results Tables S2–S7).

#### Preconception

For a person trying to conceive, panellists strongly agreed that supplement use should be advised up to 6 months before they get pregnant (combined estimates median = 2, IQR = 1, 84% agreement). There was consensus that a balanced diet is mostly not sufficient to meet all micronutrient requirements and that supplement use should be advised (76% agreement, median=-1, IQR = 1).

In the preconception period, panellists strongly agreed on the importance of supplementing with vitamin D, folic acid, iodine and iron (combined estimates median = 2–3, IQR = 0–1, 87–97% agreement). They also agreed that the evidence for supplementing with folic acid and iodine is somewhat to very strong during this phase (combined estimates median = 1–3, IQR = 1, 87–97% agreement). Although not reaching consensus, most panellists considered the evidence for supplementing with vitamin K during preconception to be somewhat to very weak (62% agreement, median=-1.5, IQR = 2), attributing this primarily to a lack of evidence.

#### First trimester

For people in the first trimester, consensus was reached on the importance of supplementing with vitamin D, folic acid, iodine, iron and DHA (combined estimates median = 2–3, IQR = 1–1.5, 75–100% agreement). Panellists also agreed that the evidence for supplementing with vitamin D, folic acid and iodine during this stage is somewhat to very strong (combined estimates median = 2–3, IQR = 1–2, 81–100% agreement). Despite not reaching consensus, the majority of panellists regarded the evidence for supplementing with vitamins B2 and K to be somewhat to very weak (combined estimates median=-1, IQR = 2, 52–62% agreement), with most ascribing this to a lack of evidence.

#### Second trimester

For the second trimester, there was consensus agreement on the importance of supplementing with the same nutrients as for the first trimester: vitamin D, folic acid, iodine, iron and DHA (combined estimates median = 2, IQR = 1–2, 77–90% agreement). Consensus was also reached that the strength of evidence for supplementing with each of these nutrients during the second trimester is somewhat to very strong (combined estimates median = 1–2, IQR = 1–2, 77–88% agreement). Although not reaching consensus, most panellists considered the evidence for supplementing with vitamins B2 and K during the second trimester to be somewhat to very weak (combined estimates median=-1–1.5, IQR = 2, 52–58% agreement), primarily due to a lack of evidence.

#### Third trimester

In the third trimester, panellists reached consensus on the importance of supplementing with vitamin D, iodine, iron and DHA (combined estimates median = 2, IQR = 1–1.5, 75–91% agreement). There was also consensus that the strength of evidence for supplementing with each of these nutrients at this stage is somewhat to very strong (combined estimates median = 1–2, IQR = 1–1.3, 87–90% agreement). Despite not reaching consensus, most panellists rated the evidence for supplementing with selenium and vitamins B1, B2 and K during the third trimester to be somewhat to very weak (combined estimates median=-1, IQR = 2–3, 52–57% agreement), mainly ascribing this to a lack of evidence.

#### Postpartum/lactation

During lactation, there was consensus agreement on the importance of supplementing with vitamin D, iron, DHA and calcium (combined estimates median = 1–2, IQR = 1, 78–90% agreement). Panellists also agreed that the strength of evidence for supplementing with each of these nutrients in this phase is somewhat to very strong (combined estimates median = 1–2, IQR = 1, 81–90% agreement). While there was no consensus on the strength of evidence for other micronutrients, the majority of panellists regarded the evidence for supplementing with choline, selenium and vitamins B1, B2, B6 and K during lactation to be somewhat to very weak, with most again attributing this to a lack of evidence (combined estimates median=-1, IQR = 2–3, 52–62% agreement). There was no consensus on whether people should be advised to continue supplement use post-weaning or if people who are not breastfeeding should continue postpartum.

### Approaches to nutrition in specific at-risk groups during pregnancy

Panellists reached consensus on the need to tailor dietary supplement use recommendations to a person’s lifestyle, medical conditions and nutrition-related pregnancy complications (combined estimates median = 2–3, IQR = 1–2, 88–100% agreement; Additional File 3: Supplementary Results Table S8). There was strong consensus that dietary supplement use recommendations are not universally applicable (84% agreement, median=-2, IQR = 1).

Although there were a relatively high number of ‘NA’ responses to questions in this section, panellists agreed that approaches to diet and dietary supplement use during pregnancy should be tailored for each of the examined groups, including athletes/highly active people, people following vegan/vegetarian diets, restricted diets due to food intolerance, obesity, polycystic ovary disease (PCOS), diabetes mellitus and/or a history of birth complications such as NTDs, pre-eclampsia, PTB or gestational diabetes (combined estimates median = 2–3, IQR = 0–1, 86–100% agreement). In free-text comments, panellists noted the need to consider a person’s biomarkers, nutritional status and the nature of any disease/complications. In each of the examined groups, there was consensus agreement on the importance of adjusting supplement recommendations for vitamin D, iron and DHA (Additional file 3: Supplementary Results Table S7). For other nutrients, ratings of importance varied considerably between the groups.

## Discussion

This Delphi study examined the level of expert consensus on optimal maternal nutrition including diet and micronutrient supplement use. An international multidisciplinary panel, with expertise in specialties such as nutrition, gynaecology and obstetrics, participated and reached consensus in key areas of uncertainty, including general considerations around diet and dietary supplement use, the importance of supplementing with folic acid, DHA, iron, iodine, calcium and vitamin D during distinct phases of preconception, pregnancy and lactation, and the at-risk groups requiring tailored approaches.

Evidence suggests that micronutrient intake is generally lower than recommended, even in high-income countries [42, 43]. Examples of shortfalls at the population level in achieving recommended levels have been reported for vitamin D [35, 44], folate [45–47], iodine [30, 47, 48], vitamin B12 [49, 50], iron [47] and DHA [51], which can be particularly difficult to achieve via diet during pregnancy and lactation, when some fish species should be avoided due to heavy metal content [30, 52, 53].

Among the key general insights, panellists strongly agreed on providing advice to follow a Mediterranean diet, in line with several studies demonstrating that a maternal healthy diet and lifestyle contribute to improving short- and long-term reproductive and pregnancy outcomes [54–56]. The Mediterranean diet can be a good source of folate, DHA, vitamin D and other vitamins, as it emphasises foods that are rich in antioxidants, vitamins, minerals, polyphenols and fibre, i.e. vegetables, fruits, grains, nuts, olive oil, fish and seafood, and white meat [40]. Adherence to the Mediterranean diet during pregnancy has been shown to be advantageous, especially with regard to reducing the risk of gestational diabetes and gestational weight gain [57]. However, it is not clear which component(s) of the diet these positive effects might be attributed to, and barriers to adoption exist in countries with limited access to the types of foods necessary for this diet.

However, panellists also agreed on the lack of clarity and consistency in current guidelines, and the need for education in these areas for HCPs, pregnant people and the public. These findings align well with previous surveys of gynaecologists in which uncertainty around micronutrient supplement use during pregnancy was emphasised, along with the lack of widely recognised guidelines for gynaecologists [7, 58]. Altogether, this highlights the need for standardised, clear guidelines to ensure consistent and evidence-based recommendations.

### Consensus findings for micronutrient supplement use in pregnancy and lactation in general (low-risk) populations

#### Folate

There was strong consensus for folic acid supplement use during the preconception period and the first trimester, which is in line with existing guidelines to take supplemental folic acid (vitamin B9) to reduce the risk of NTDs. Obstetricians and gynaecologists are generally aware of the importance of folic acid intake during preconception and the first trimester, but may be less aware of the role of folic acid in prevention of other congenital defects [59] as a substrate in one-carbon metabolism [58, 60–62]. In addition, folic acid supplement use during early pregnancy (4 weeks before to 8 weeks after conception) appears to benefit neurocognitive development of the child [63]. However, the potential advantages of folic acid supplement use beyond the first trimester are unclear. A recent systematic review and meta-analysis (three randomised controlled trials [RCTs] and 17 cohort studies) has shown that folic acid supplement use during pregnancy was associated with a lower risk of pre-eclampsia, although the timing of supplement use was not specified [64]. Folic acid supplement use after the first trimester has been shown to increase maternal and cord blood folate levels and prevent the increase in homocysteine normally seen late in the second and third trimesters [65, 66]. However, given that these studies did not include pregnancy outcomes and that current guidelines only cover preconception and early pregnancy, the lack of consensus for folic acid during the third trimester may be expected.

#### Other B vitamins

Consensus for other B vitamins (B1, B2, B6, B12) besides folic acid was weak. Similar to folate, vitamins B2, B6 and B12 are involved as cofactors in one-carbon metabolism and are therefore likely to be relevant for foetal development [67]. For example, studies have shown that using multivitamin supplements containing folic acid and iron in addition to other nutrients (vitamins B1, B2, B3, B6, B12, vitamins A, D, E, omega-3 fatty acids and minerals) was more likely to show a positive impact on pregnancy outcomes than those containing only folic acid and iron [67]. Using multi-micronutrients including folic acid and iron could be of special importance among women from low- and middle-income countries where multiple micronutrient deficiencies are common [67]. Vitamin B12 supplement use during pregnancy could lower the risk of deficiency in the mother during pregnancy and lactation [68]. The evidence on pregnancy outcomes such as PTB and low birth weight is uncertain due to the low number of studies available [68]. Unrecognised vitamin B12 deficiency in the mother is a risk factor for vitamin B12 deficiency in newborns, which can manifest in infants at age 3–6 months as serious developmental delay and neurological symptoms. A German pilot study of newborns reported a prevalence rate for vitamin B12 deficiency of 1:3586 births (95% confidence interval 1:3577–1:3596) [69]. If antenatal supplements contain the adequate dose of vitamin B12, this deficiency could be prevented in mothers and neonates. Thus far, methodological limitations prevent definitive conclusions on specific clinical outcomes related to each of the B vitamins [68]. This could explain why B vitamins (other than folic acid) are not yet embedded in current guidelines, and the weak to no consensus for B vitamins in this Delphi.

#### DHA

There was consensus agreement on the importance of supplementing with DHA across all three trimesters of pregnancy and throughout the lactation period. Interestingly, when voting on the strength of evidence that DHA supplement use improves pregnancy outcomes and/or foetal/neonatal development, a consensus of ≥ 75% was reached for the second and third trimesters and lactation, but not for the first trimester.

DHA/omega-3 fatty acid supplement use in pregnancy has been shown to reduce risk of PTB and early preterm birth (EPB). A recent Cochrane review found an 11% risk reduction of PTB < 37 weeks, and a 42% risk reduction of EPB < 34 weeks [20], but in most studies supplement use only started in the second trimester, around 20 weeks of gestation. A recent clinical practice guideline based on a review of the available evidence and a formal consensus process advocates DHA and eicosapentaenoic acid supplement use in pregnancy for risk reduction of PTB and EPB [70].

Positive effects of DHA/omega-3 long-chain polyunsaturated fatty acids on pregnancy outcomes are likely driven by the downregulation of inflammation, mediated by changing the balance between pro- and anti-inflammatory prostaglandins in the uterus [71]. Moreover, the accumulation of DHA in the brain in the third trimester and first year of life may have positive effects on brain and cognitive development [72].

#### Iron

There was consensus agreement on the importance of iron supplement use in both general (low-) and high-risk populations across all stages of female reproduction, with consensus agreement on the improvement of pregnancy, foetal and neonatal outcomes for the second and third trimesters, and lactation period. Iron-deficient anaemia is highly prevalent worldwide, with frequencies of iron deficiency ranging from 40% in early pregnancy to 80% in late pregnancy [73, 74]. This explains the consensus and awareness among experts on the need for iron supplement use from preconception to the postpartum period. Iron is essential for foetal neurogenesis and placental development, as well as haematopoiesis in mother and foetus. An increase in severe maternal morbidity and mortality [75], as well as increased risks of short- (i.e. PTB, small for gestational-age babies) and long-term (i.e. autism spectrum disorders, impaired cognitive status) adverse outcomes in the offspring [76], were described in iron-deficient anaemic pregnant women, although benefits of routine antenatal supplement use are controversial. Nevertheless, routine screening for anaemia and iron deficiency in early pregnancy and the third trimester is consistently supported as cost-effective [26, 77].

#### Iodine

The panel reached consensus agreement on the importance of iodine supplement use from preconception through all stages of pregnancy, also reaching consensus on the strength of the evidence. Pregnant women and infants are exceptionally vulnerable to iodine deficiency; poor iodine intake has been shown to cause harmful effects on the foetus, including delayed development and brain maturation [78]. Adequate iodine supplement use from preconception until the end of the first trimester of pregnancy has been shown to reduce the incidence of cretinism in the areas of highest deficiency risk by up to 73% [79, 80]. The need for iodine supplement use during pregnancy was also emphasised in a survey of German gynaecologists [7]. However, a recent Cochrane review did not find significant benefits of iodine supplement use in the preconception, pregnancy or postpartum periods, although these findings must be interpreted cautiously due to limited data and low-quality trials [81]. The widespread problem of iodine deficiency has led medical societies in Europe and North America to recommend that women planning a pregnancy, or who are pregnant or breastfeeding, take a daily tablet containing at least 150 µg of iodine [82].

#### Calcium

The importance of optimal calcium intake is seen during periods of rapid foetal growth, particularly the third trimester. Physiological adaptations with increased intestinal mineral absorption during pregnancy ensure maternal bone integrity, mineralisation of the foetal skeleton and continued skeletal growth in the neonate/infant [83]. Therefore, healthy pregnant people residing in countries where calcium intake is generally higher may not benefit from supplement use compared with women in countries where the calcium intake is low. It is possible that calcium was not specifically considered in a survey of German gynaecologists due to likely high milk intake in the population [7]. The lack of consistency in current guidelines may have led to distinctions in expert opinion on calcium supplement use during pregnancy in the present study with its focus on a European population. By contrast, during lactation, when calcium supply to breast milk is mainly derived from the skeleton of the mother [83], consensus was reached on calcium supplement use.

Beyond maintaining calcium homeostasis, a Cochrane review has linked calcium supplement use to lowering of blood pressure and prevention of pregnancy-induced hypertension and related complications [84]. However, the impact of supplement use on the risk of preterm delivery remains conflicted, as does the quality of evidence for outcomes other than hypertensive disorders. Consequently, current WHO recommendations are confined to at-risk situations [85].

#### Vitamin D

There was consensus agreement on the importance of vitamin D supplement use in the preconception phase, across all three trimesters of pregnancy and throughout the lactation period. In agreement with these recommendations, the strength/quality of evidence arguing for vitamin D supplement use to improve maternal and/or foetal/infant health was also rated as somewhat to very strong, except for the preconception period. This strong consensus in favour of widespread vitamin D supplement use may not sufficiently reflect the still ongoing scientific discussion on vitamin D in these settings [86–88]. There is compelling evidence that a poor vitamin D status, reflected by low serum 25(OH)D concentrations, is associated with various adverse pregnancy outcomes [86]. In contrast, data from vitamin D RCTs are inconsistent and often limited due to low sample sizes, enrolment starting after the first trimester, and/or including women with a sufficient vitamin D status [87, 88]. While it is beyond the scope of this manuscript to critically appraise the whole literature on vitamin D, widespread vitamin D supplement use is supported by evidence that (a) vitamin D deficiency is very common, (b) vitamin D is critical for bone health (i.e. prevention and treatment of rickets and osteomalacia) as consistently documented by evidence-based guidelines, and (c) vitamin D supplement use at moderate doses (e.g. 800–2000 IU, 20–50 µg daily) is relatively safe from preconception throughout pregnancy until lactation [86, 89]. There are also additional potential beneficial effects of vitamin D, such as for fertility, preventing pre-eclampsia or gestational diabetes mellitus, although evidence remains limited [86, 87, 90].

#### Choline

Choline supplement use in pregnancy and lactation did not achieve consensus among the panellists. Choline contributes to normal liver function and lipid metabolism in adults [91, 92]. The requirements for choline are greater during pregnancy and lactation than outside these critical time periods [91, 92], due to the need to meet the needs of both the mother and foetus or breastfed infant at the same time. Choline is actively transported through the placenta and accumulates in the foetus. Choline supplement use by the mother leads to higher choline levels in breast milk and consequently increased choline intake for breastfed infants [93]. The EFSA has recently evaluated the evidence on the role of maternal choline intake in health outcomes in the foetus and infants [94], and concluded that a sufficient intake of choline during pregnancy and lactation (480 mg/day and 520 mg/day, respectively) can contribute to normal liver function of the foetus and exclusively breastfed infants [94].

A recent meta-analysis of observational studies reported that low maternal choline intake or blood levels are associated with a higher risk of NTDs in newborns [18]. Higher choline intake is likely to have positive impacts on foetal brain development and neurodevelopment of the child after birth [18]. The growing evidence on the role of choline during pregnancy and lactation is still awaiting translation into population-wide recommendations and policy. This could explain why the panellists did not prioritise choline supplement use for pregnant and lactating people in this Delphi study.

#### Other examined micronutrients: Vitamin K

Consensus among panellists was that the evidence for vitamin K supplement use was weak. This reflects the current lack of evidence, with most pregnancy and neonatal outcomes unassessed, not significant or providing very low-quality evidence, and few trials reporting neonatal adverse effects. A study of women admitted for elective caesarean section found that vitamin K supplement use was favourable for maternal bleeding but was of limited value for neonatal bleeding and other outcomes [95]. The existing literature gaps warrant future investigations on un-assessed or inadequately reported outcomes [96].

### Consensus findings for specific at-risk groups

People following vegan/vegetarian diets, restricted diets, or with obesity, PCOS, diabetes or a history of pregnancy complications were identified by panellists as groups for whom supplement use advice should be tailored.

Particularly, the global “diabesity” pandemic involves a growing number of women of reproductive age and is associated with increased risk for PCOS and infertility, gestational diabetes, and other pregnancy and birth complications [97]. Also, a recent systematic review revealed that maternal obesity increases the risk for nutritional deficiencies, especially folate, vitamin D and vitamin B12 [98]. Hence the panellists’ concern that tailored recommendations for people living with obesity and planning pregnancy should be developed. For women with a history of bariatric surgery, a well-recognised high-risk group for nutritional deficiencies [99], evidence-based guidelines have recently been developed [100]. Of note, panellists emphasised the need for more research on dietary/nutritional requirements in these specific at-risk groups. Ethical concerns around allocating specific at-risk groups of pregnant people to placebo arms in large micronutrient RCTs may, however, limit our ability to clarify several remaining knowledge gaps during preconception and pregnancy in the future.

### Limitations

While the Delphi method is recognised as reliable and robust, it is not without limitations, including the lack of standardised criteria for defining consensus and the possibility that anonymity may lead to insufficiently considered responses. Additionally, in the present study, it is possible that the broad scope of the Delphi surveys, spanning different areas of expertise and employing equal weighting of responses, influenced consensus and contributed to the high prevalence of ‘NA’ responses for certain questions. Although there was representation from a range of specialties, the panel selection method may have resulted in panellists with narrow expertise and contributed to the observed variability in responses. The possible bias towards the northern hemisphere and developed countries may also have impacted the generalisability of the findings. Another limitation is that we did not specifically account for between-country differences in diet and food fortification.

### Future directions

Future research is needed to address the evidence gaps highlighted by the Delphi findings, which confirmed strong evidence for maternal supplement use of some micronutrients, yet consistently pointed to a lack or low level of evidence for others. The variability in panel responses and lack of consensus around micronutrients such as choline and vitamin B12, despite accumulating evidence of their benefit, may suggest knowledge gaps among HCPs. This reinforces the importance of developing targeted educational programmes for those involved in maternal care, care of people planning or preparing to become pregnant, and creating accessible lay communications for the public. Alongside educational efforts, there is a pressing need for harmonised supplement use guidelines that reflect the latest evidence. The generalisability of results must be addressed, with studies needed in different populations and research ideally led by an international consortium. Nutritional research is, in general, complicated: as people eat diets and not nutrients, it is difficult to study the effect of a single nutrient on a study participant in a supplement study, because we might miss the interaction with other nutrients. Therefore, we would suggest conducting studies in populations with deficiencies of selected micronutrients, for example to better characterise the role of adequate vitamin B12 in pregnancy and birth outcomes for women following vegan diets. It is also important to study the long-term effects on offspring health in populations where nutritional data have been collected during pregnancy. The development of harmonised supplement use guidelines is more relevant than ever, both for women and for healthcare providers, and scientific societies and institutions need to be involved to reach consensus on guidelines. Nutrient and supplement guidelines should balance general and personalised approaches, as well as considering the toxicity of high dosages and cost-effectiveness for low- or general-risk populations.

Similar Delphi approaches could prove valuable in exploring areas of consensus, as well as evidence and knowledge gaps in the field of nutraceuticals, in particular approaches to pre-, pro-, syn- and postbiotics.

## Conclusions

This Delphi study revealed robust consensus on various aspects of maternal nutrition, including the need for education/awareness around maternal diet and dietary supplement use, the lack of clarity and consistency in current guidelines, the importance of supplementing with folic acid, vitamin D, iron, iodine, DHA and calcium during preconception and across specific phases of pregnancy, and the at-risk groups requiring tailored approaches. The lack of consensus around several other micronutrients, despite growing evidence of their benefit, reinforces the need for prompt translation of available evidence into guidelines, policies and practice. Future initiatives are required to facilitate the development of harmonised supplement use guidelines that reflect the latest evidence and to enhance maternal nutrition education in order to improve the health of at least two generations.

## Electronic supplementary material

Below is the link to the electronic supplementary material.

Supplementary Material 1: Additional File 1

Supplementary Material 2: Additional File 2

Supplementary Material 3: Additional File 3

## Data Availability

All data generated or analysed during this study are included in this published article (and its supplementary information files) – and an overview of the free-text comments is available from the corresponding author on reasonable request.

## Abbreviations

BfR: German Federal Institute of Risk Assessment
CFR: Code of Federal Regulations
D–A–CH: (Germany, Austria, Switzerland)
DHA: Docosahexaenoic acid
EFSA: European Food Safety Authority
EPB: Early preterm birth
HCP: Healthcare professional
IOM: US Institute of Medicine
IQR: Interquartile range
IU: International units
NA: Not applicable
NTD: Neural tube defect
PCOS: Polycystic ovary disease
PTB: Preterm birth
RCT: Randomised controlled trial
RDI: Recommended dietary intake
SACN: Standing Advisory Committee on Nutrition
SC: Steering committee
UNICEF: United Nations Children’s Fund
WHO: World Health Organization

## References

[1] van Zundert SKM, van Rossem L, Schoenmakers S, Steegers-Theunissen RPM. Lifestyle care for transformation of medical care using an early life course approach. Reprod Biomed Online. 2022;45:847–50. 36130853 10.1016/j.rbmo.2022.08.096

[2] Berti C, Decsi T, Dykes F, Hermoso M, Koletzko B, Massari M, et al. Critical issues in setting micronutrient recommendations for pregnant women: an insight. Matern Child Nutr. 2010;6:5–22. 22296248 10.1111/j.1740-8709.2010.00269.x PMC6860719

[3] Black RE. Micronutrients in pregnancy. Br J Nutr. 2001;85:S193–7. 11509110 10.1079/bjn2000314

[4] Koletzko B, Bauer CP, Bung P, Cremer M, Flothkotter M, Hellmers C, et al. German national consensus recommendations on nutrition and lifestyle in pregnancy by the ‘Healthy start - Young family network’. Ann Nutr Metab. 2013;63:311–22. 24514069 10.1159/000358398

[5] Mousa A, Naqash A, Lim S. Macronutrient and micronutrient intake during pregnancy: an overview of recent evidence. Nutrients. 2019;11:443. 30791647 10.3390/nu11020443 PMC6413112

[6] Marshall NE, Abrams B, Barbour LA, Catalano P, Christian P, Friedman JE, et al. The importance of nutrition in pregnancy and lactation: lifelong consequences. Am J Obstet Gynecol. 2022;226:607–32. 34968458 10.1016/j.ajog.2021.12.035 PMC9182711

[7] Buhling KJ, Scheuer M, Laakmann E. Recommendation and intake of dietary supplements periconceptional and during pregnancy: results of a nationwide survey of gynaecologists. Arch Gynecol Obstet. 2023;308:1863–9. 37715805 10.1007/s00404-023-07167-6 PMC10579106

[8] Schenkelaars N, Schoenmakers S, Rousian M, Willemsen SP, Faas MM, Steegers-Theunissen RPM. Periconceptional maternal supplement intake and human embryonic growth, development, and birth outcomes: the Rotterdam Periconception Cohort. Hum Reprod. 2024;39:1925–33. 10.1093/humrep/deae168 PMC11373404 39025484

[9] Hanson MA, Bardsley A, De-Regil LM, Moore SE, Oken E, Poston L, et al. The International Federation of Gynecology and Obstetrics (FIGO) recommendations on adolescent, preconception, and maternal nutrition: “Think Nutrition First”. Int J Gynaecol Obstet. 2015;131:S213–53. 26433230 10.1016/S0020-7292(15)30034-5

[10] De-Regil LM, Pena-Rosas JP, Fernandez-Gaxiola AC, Rayco-Solon P. Effects and safety of periconceptional oral folate supplementation for preventing birth defects. Cochrane Database Syst Rev. 2015;2015:CD007950. 26662928 10.1002/14651858.CD007950.pub3 PMC8783750

[11] Fekete K, Berti C, Trovato M, Lohner S, Dullemeijer C, Souverein OW, et al. Effect of folate intake on health outcomes in pregnancy: a systematic review and meta-analysis on birth weight, placental weight and length of gestation. Nutr J. 2012;11:75. 22992251 10.1186/1475-2891-11-75 PMC3499376

[12] Brannon PM, Taylor CL. Iron supplementation during pregnancy and infancy: uncertainties and implications for research and policy. Nutrients. 2017;9:1327. 29210994 10.3390/nu9121327 PMC5748777

[13] Pena-Rosas JP, De-Regil LM, Garcia-Casal MN, Dowswell T. Daily oral iron supplementation during pregnancy. Cochrane Database Syst Rev. 2015;2015:CD004736. 26198451 10.1002/14651858.CD004736.pub5 PMC8918165

[14] Zimmer M, Sieroszewski P, Oszukowski P, Huras H, Fuchs T, Pawlosek A. Polish society of gynecologists and obstetricians recommendations on supplementation during pregnancy. Ginekol Pol. 2020;91:644–53. 33184834 10.5603/GP.2020.0159

[15] Leung AM, Pearce EN, Braverman LE. Iodine nutrition in pregnancy and lactation. Endocrinol Metab Clin North Am. 2011;40:765–77. 22108279 10.1016/j.ecl.2011.08.001 PMC3266621

[16] Moon RJ, Green HD, D’Angelo S, Godfrey KM, Davies JH, Curtis EM, et al. The effect of pregnancy vitamin D supplementation on offspring bone mineral density in childhood: a systematic review and meta-analysis. Osteoporos Int. 2023;34:1269–79. 37103591 10.1007/s00198-023-06751-5

[17] Mousa A, Abell S, Scragg R, de Courten B. Vitamin D in reproductive health and pregnancy. Semin Reprod Med. 2016;34:e1–13. 27228115 10.1055/s-0036-1583529

[18] Obeid R, Derbyshire E, Schon C. Association between maternal choline, fetal brain development, and child neurocognition: systematic review and meta-analysis of human studies. Adv Nutr. 2022;13:2445–57. 36041182 10.1093/advances/nmac082 PMC9776654

[19] Zeisel SH. Nutrition in pregnancy: the argument for including a source of choline. Int J Women’s Health. 2013;5:193–9. 23637565 10.2147/IJWH.S36610 PMC3639110

[20] Middleton P, Gomersall JC, Gould JF, Shepherd E, Olsen SF, Makrides M. Omega-3 fatty acid addition during pregnancy. Cochrane Database Syst Rev. 2018;11:CD003402. 30480773 10.1002/14651858.CD003402.pub3 PMC6516961

[21] Braarud HC, Markhus MW, Skotheim S, Stormark KM, Froyland L, Graff IE, Kjellevold M. Maternal DHA status during pregnancy has a positive impact on infant problem solving: a Norwegian prospective observation study. Nutrients. 2018;10:529. 29695097 10.3390/nu10050529 PMC5986409

[22] Thomas M, Weisman SM. Calcium supplementation during pregnancy and lactation: effects on the mother and the fetus. Am J Obstet Gynecol. 2006;194:937–45. 16580279 10.1016/j.ajog.2005.05.032

[23] Institute of Medicine Panel on Micronutrients. Dietary reference intakes for vitamin A, vitamin K, arsenic, boron, chromium, copper, iodine, iron, manganese, molybdenum, nickel, silicon, vanadium, and zinc. Washington (DC): National Academies Press (US); 2001. 25057538

[24] EFSA Panel on Dietetic Products. Nutrition and allergies. Scientific opinion on dietary reference values for iron. EFSA J. 2015;13:4254.

[25] World Health Organization. WHO recommendations on antenatal care for a positive pregnancy experience. 2016. https://iris.who.int/bitstream/handle/10665/250796/9789241549912-eng.pdf 28079998

[26] Pavord S, Daru J, Prasannan N, Robinson S, Stanworth S, Girling J, BSH Committee. UK guidelines on the management of iron deficiency in pregnancy. Br J Haematol. 2020;188:819–30. 31578718 10.1111/bjh.16221

[27] Italian Society of Human Nutrition. [LARN IV Revision]. 2024. https://eng.sinu.it/larn/

[28] National Health and Medical Research Council (Australia). Public Statement: Iodine supplementation for pregnant and breastfeeding women. 2010. https://www.nhmrc.gov.au/about-us/publications/iodine-supplementation-pregnant-and-breastfeeding-women

[29] National Health and Medical Research Council (Australia). Australian Dietary Guidelines. 2013. https://www.eatforhealth.gov.au/sites/default/files/2022-09/n55_australian_dietary_guidelines.pdf

[30] German Federal Institute for Risk Assessment. Iodine intake in Germany on the decline again - tips for a good iodine intake. 2021. https://www.bfr.bund.de/cm/349/iodine-intake-in-germany-on-the-decline-again-tips-for-a-good-iodine-intake.pdf

[31] German Society for Nutrition. Iodine. https://www.dge.de/wissenschaft/referenzwerte/jod/

[32] World Health Organization. Reaching optimal iodine nutrition in pregnant and lactating women and young children. 2007. https://cdn.who.int/media/docs/default-source/nutritionlibrary/reaching-optimal-iodine-nutrition-in-pregnant-and-lactating-women-and-young-children.pdf 10.1017/s1368980007705360 18333291

[33] EFSA Panel on Dietetic Products. Nutrition and allergies. Dietary reference values for vitamin D. EFSA J. 2016;14:e04547.

[34] Institute of Medicine Committee to Review Dietary Reference Intakes for Vitamin D., Calcium: The National Academies Collection: Reports funded by National Institutes of Health. In: Dietary Reference Intakes for Calcium and Vitamin D. edn. Edited by Ross AC, Taylor CL, Yaktine AL. Del Valle HB. Washington (DC): National Academies Press (US); 2011. 21796828

[35] German Society for Nutrition. New reference values for vitamin D. Ann Nutr Metab. 2012;60:241–6. 22677925 10.1159/000337547

[36] Holick MF, Binkley NC, Bischoff-Ferrari HA, Gordon CM, Hanley DA, Heaney RP, et al. Evaluation, treatment, and prevention of vitamin D deficiency: an Endocrine Society clinical practice guideline. J Clin Endocrinol Metab. 2011;96:1911–30. 21646368 10.1210/jc.2011-0385

[37] Pludowski P, Karczmarewicz E, Bayer M, Carter G, Chlebna-Sokol D, Czech-Kowalska J, et al. Practical guidelines for the supplementation of vitamin D and the treatment of deficits in Central Europe - recommended vitamin D intakes in the general population and groups at risk of vitamin D deficiency. Endokrynol Pol. 2013;64:319–27. 24002961 10.5603/ep.2013.0012

[38] Murphy MK, Black NA, Lamping DL, McKee CM, Sanderson CF, Askham J, Marteau T. Consensus development methods, and their use in clinical guideline development. Health Technol Assess. 1998;2:i–iv. 9561895

[39] Beiderbeck D, Frevel N, von der Gracht HA, Schmidt SL, Schweitzer VM. Preparing, conducting, and analyzing Delphi surveys: cross-disciplinary practices, new directions, and advancements. MethodsX. 2021;8:101401. 34430297 10.1016/j.mex.2021.101401 PMC8374446

[40] Sikalidis AK, Kelleher AH, Kristo AS. Mediterranean diet. Encyclopedia. 2021;1:371–87.

[41] Diamond IR, Grant RC, Feldman BM, Pencharz PB, Ling SC, Moore AM, Wales PW. Defining consensus: a systematic review recommends methodologic criteria for reporting of Delphi studies. J Clin Epidemiol. 2014;67:401–9. 24581294 10.1016/j.jclinepi.2013.12.002

[42] Parisi F, Laoreti A, Cetin I. Multiple micronutrient needs in pregnancy in industrialized countries. Ann Nutr Metab. 2014;65:13–21. 25227491 10.1159/000365794

[43] Berti C, Cetin I, Agostoni C, Desoye G, Devlieger R, Emmett PM, et al. Pregnancy and infants’ outcome: nutritional and metabolic implications. Crit Rev Food Sci Nutr. 2016;56:82–91. 24628089 10.1080/10408398.2012.745477

[44] Spiro A, Buttriss JL, Vitamin D. An overview of vitamin D status and intake in Europe. Nutr Bull. 2014;39:322–50. 25635171 10.1111/nbu.12108 PMC4288313

[45] Obeid R, Schön C, Wilhelm M, Pietrzik K, Pilz S. The effectiveness of daily supplementation with 400 or 800 µg/day folate in reaching protective red blood folate concentrations in non-pregnant women: a randomized trial. Eur J Nutr. 2018;57:1771–80. 28447203 10.1007/s00394-017-1461-8 PMC6060806

[46] Mensink GBM, Weißenborn A, Richter A. Folate status in Germany. J Health Monit. 2016;1:24–8. 36654830 10.17886/RKI-GBE-2016-040.2 PMC9838576

[47] Stephenson J, Heslehurst N, Hall J, Schoenaker D, Hutchinson J, Cade JE, et al. Before the beginning: nutrition and lifestyle in the preconception period and its importance for future health. Lancet. 2018;391:1830–41. 29673873 10.1016/S0140-6736(18)30311-8 PMC6075697

[48] Alexander EK, Pearce EN, Brent GA, Brown RS, Chen H, Dosiou C, et al. Guidelines of the American thyroid association for the diagnosis and management of thyroid disease during pregnancy and the postpartum. Thyroid. 2017;27:315–89. 28056690 10.1089/thy.2016.0457

[49] Sukumar N, Rafnsson SB, Kandala NB, Bhopal R, Yajnik CS, Saravanan P. Prevalence of vitamin B-12 insufficiency during pregnancy and its effect on offspring birth weight: a systematic review and meta-analysis. Am J Clin Nutr. 2016;103:1232–51. 27076577 10.3945/ajcn.115.123083

[50] Al-Musharaf S, McTernan PG, Hussain SD, Aleisa KA, Alnaami AM, Wani K et al. Prevalence and indicators of vitamin B12 insufficiency among young women of childbearing age. Int J Environ Res Public Health 2020;18:1. 10.3390/ijerph18010001 PMC7792587 33374905

[51] Sioen I, van Lieshout L, Eilander A, Fleith M, Lohner S, Szommer A, et al. Systematic review on N-3 and N-6 polyunsaturated fatty acid intake in European countries in light of the current recommendations - focus on specific population groups. Ann Nutr Metab. 2017;70:39–50. 28190013 10.1159/000456723 PMC5452278

[52] Royal College of Obstetricians & Gynaecologists. Healthy eating and vitamin supplements in pregnancy. 2022. https://www.rcog.org.uk/media/nkvpl2mn/healthy-eating-vitamin-supplements-pregnancy-patient-information.pdf

[53] EFSA Scientific Committee. Statement on the benefits of fish/seafood consumption compared to the risks of methylmercury in fish/seafood. EFSA J. 2015;13:3982.

[54] Timmermans S, Steegers-Theunissen RP, Vujkovic M, den Breeijen H, Russcher H, Lindemans J, et al. The Mediterranean diet and fetal size parameters: the Generation R Study. Br J Nutr. 2012;108:1399–409. 22348517 10.1017/S000711451100691X

[55] Vujkovic M, de Vries JH, Lindemans J, Macklon NS, van der Spek PJ, Steegers EA, Steegers-Theunissen RP. The preconception Mediterranean dietary pattern in couples undergoing in vitro fertilization/intracytoplasmic sperm injection treatment increases the chance of pregnancy. Fertil Steril. 2010;94:2096–101. 20189169 10.1016/j.fertnstert.2009.12.079

[56] Vujkovic M, Steegers EA, Looman CW, Ocke MC, van der Spek PJ, Steegers-Theunissen RP. The maternal Mediterranean dietary pattern is associated with a reduced risk of spina bifida in the offspring. BJOG. 2009;116:408–15. 19187373 10.1111/j.1471-0528.2008.01963.x

[57] Zhang Y, Xia M, Weng S, Wang C, Yuan P, Tang S. Effect of Mediterranean diet for pregnant women: a meta-analysis of randomized controlled trials. J Matern Fetal Neonatal Med. 2022;35:4824–9. 33632052 10.1080/14767058.2020.1868429

[58] Power ML, Holzman GB, Schulkin J. Knowledge and clinical practice regarding folic acid among obstetrician-gynecologists. Obstet Gynecol. 2000;95:895–8. 10831987 10.1016/s0029-7844(00)00793-6

[59] Czeizel AE. Periconceptional folic acid and multivitamin supplementation for the prevention of neural tube defects and other congenital abnormalities. Birth Defects Res Clin Mol Teratol. 2009;85:260–8. 10.1002/bdra.20563 19161162

[60] Steegers-Theunissen RP, Boers GH, Trijbels FJ, Eskes TK. Neural-tube defects and derangement of homocysteine metabolism. N Engl J Med. 1991;324:199–200. 1984202 10.1056/NEJM199101173240315

[61] Steegers-Theunissen RP, Boers GH, Trijbels FJ, Finkelstein JD, Blom HJ, Thomas CM, et al. Maternal hyperhomocysteinemia: a risk factor for neural-tube defects? Metabolism. 1994;43:1475–80. 7990699 10.1016/0026-0495(94)90004-3

[62] Steegers-Theunissen RP, Smithells RW, Eskes TK. Update of new risk factors and prevention of neural-tube defects. Obstet Gynecol Surv. 1993;48:287–93. 8492996 10.1097/00006254-199305000-00002

[63] Roth C, Magnus P, Schjolberg S, Stoltenberg C, Suren P, McKeague IW, et al. Folic acid supplements in pregnancy and severe language delay in children. JAMA. 2011;306:1566–73. 21990300 10.1001/jama.2011.1433 PMC3780384

[64] Yu Y, Sun X, Wang X, Feng X. The association between the risk of hypertensive disorders of pregnancy and folic acid: a systematic review and meta-analysis. J Pharm Pharm Sci. 2021;24:174–90. 33878280 10.18433/jpps31500

[65] McNulty B, McNulty H, Marshall B, Ward M, Molloy AM, Scott JM, et al. Impact of continuing folic acid after the first trimester of pregnancy: findings of a randomized trial of folic acid supplementation in the second and third trimesters. Am J Clin Nutr. 2013;98:92–8. 23719554 10.3945/ajcn.112.057489

[66] Holmes VA, Wallace JM, Alexander HD, Gilmore WS, Bradbury I, Ward M, et al. Homocysteine is lower in the third trimester of pregnancy in women with enhanced folate status from continued folic acid supplementation. Clin Chem. 2005;51:629–34. 15615817 10.1373/clinchem.2004.032698

[67] Keats EC, Haider BA, Tam E, Bhutta ZA. Multiple-micronutrient supplementation for women during pregnancy. Cochrane Database Syst Rev. 2019;3:CD004905. 30873598 10.1002/14651858.CD004905.pub6 PMC6418471

[68] Finkelstein JL, Fothergill A, Venkatramanan S, Layden AJ, Williams JL, Crider KS, Qi YP. Vitamin B12 supplementation during pregnancy for maternal and child health outcomes. Cochrane Database Syst Rev. 2024;1:CD013823. 38189492 10.1002/14651858.CD013823.pub2 PMC10772977

[69] Schnabel E, Kolker S, Gleich F, Feyh P, Horster F, Haas D, et al. Combined newborn screening allows comprehensive identification also of attenuated phenotypes for methylmalonic acidurias and homocystinuria. Nutrients. 2023;15:3355. 37571294 10.3390/nu15153355 PMC10420807

[70] Cetin I, Carlson SE, Burden C, da Fonseca EB, di Renzo GC, Hadjipanayis A, et al. Omega-3 fatty acid supply in pregnancy for risk reduction of preterm and early preterm birth. Am J Obstet Gynecol MFM. 2023;6:101251. 38070679 10.1016/j.ajogmf.2023.101251

[71] Aung MT, Yu Y, Ferguson KK, Cantonwine DE, Zeng L, McElrath TF, et al. Prediction and associations of preterm birth and its subtypes with eicosanoid enzymatic pathways and inflammatory markers. Sci Rep. 2019;9:17049. 31745121 10.1038/s41598-019-53448-z PMC6863859

[72] Lauritzen L, Brambilla P, Mazzocchi A, Harslof LB, Ciappolino V, Agostoni C. DHA effects in brain development and function. Nutrients. 2016;8:6. 26742060 10.3390/nu8010006 PMC4728620

[73] Auerbach M, Abernathy J, Juul S, Short V, Derman R. Prevalence of iron deficiency in first trimester, nonanemic pregnant women. J Matern Fetal Neonatal Med. 2021;34:1002–5. 31154873 10.1080/14767058.2019.1619690

[74] Cochrane KM, Hutcheon JA, Karakochuk CD. Iron-deficiency prevalence and supplementation practices among pregnant women: a secondary data analysis from a clinical trial in Vancouver, Canada. J Nutr. 2022;152:2238–44. 35687377 10.1093/jn/nxac135 PMC9535446

[75] Daru J, Zamora J, Fernandez-Felix BM, Vogel J, Oladapo OT, Morisaki N, et al. Risk of maternal mortality in women with severe anaemia during pregnancy and post partum: a multilevel analysis. Lancet Glob Health. 2018;6:e548–54. 29571592 10.1016/S2214-109X(18)30078-0

[76] Wiegersma AM, Dalman C, Lee BK, Karlsson H, Gardner RM. Association of prenatal maternal anemia with neurodevelopmental disorders. JAMA Psychiatry. 2019;76:1294–304. 31532497 10.1001/jamapsychiatry.2019.2309 PMC6751782

[77] Georgieff MK. Maternal gestational iron status and infant haematological and neurodevelopmental outcomes. BJOG. 2023;130:92–8. 37530464 10.1111/1471-0528.17612

[78] Jansen TA, Korevaar TIM, Mulder TA, White T, Muetzel RL, Peeters RP, Tiemeier H. Maternal thyroid function during pregnancy and child brain morphology: a time window-specific analysis of a prospective cohort. Lancet Diabetes Endocrinol. 2019;7:629–37. 31262704 10.1016/S2213-8587(19)30153-6

[79] Chittimoju SB, Pearce EN. Iodine deficiency and supplementation in pregnancy. Clin Obstet Gynecol. 2019;62:330–8. 30829881 10.1097/GRF.0000000000000428

[80] Marangoni F, Cetin I, Verduci E, Canzone G, Giovannini M, Scollo P, et al. Maternal diet and nutrient requirements in pregnancy and breastfeeding. An Italian consensus document. Nutrients. 2016;8:629. 27754423 10.3390/nu8100629 PMC5084016

[81] Harding KB, Pena-Rosas JP, Webster AC, Yap CM, Payne BA, Ota E, De-Regil LM. Iodine supplementation for women during the preconception, pregnancy and postpartum period. Cochrane Database Syst Rev. 2017;3:CD011761. 28260263 10.1002/14651858.CD011761.pub2 PMC6464647

[82] Lopes CA, Prazeres S, Martinez-de-Oliveira J, Limbert E, Lemos MC. Iodine supplementation in pregnancy in an iodine-deficient region: a cross-sectional survey. Nutrients. 2022;14:1393. 35406006 10.3390/nu14071393 PMC9002466

[83] Bollerslev J, Rejnmark L, Zahn A, Heck A, Appelman-Dijkstra NM, Cardoso L, et al. European expert consensus on practical management of specific aspects of parathyroid disorders in adults and in pregnancy: recommendations of the ESE Educational Program of Parathyroid Disorders. Eur J Endocrinol. 2022;186:R33–63. 34863037 10.1530/EJE-21-1044 PMC8789028

[84] Hofmeyr GJ, Lawrie TA, Atallah AN, Torloni MR. Calcium supplementation during pregnancy for preventing hypertensive disorders and related problems. Cochrane Database Syst Rev. 2018;10:CD001059. 30277579 10.1002/14651858.CD001059.pub5 PMC6517256

[85] World Health Organization. Guideline: Calcium supplementation in pregnant women. 2023. https://www.who.int/tools/elena/interventions/calcium-pregnancy 24006556

[86] Pilz S, Zittermann A, Obeid R, Hahn A, Pludowski P, Trummer C, et al. The role of vitamin D in fertility and during pregnancy and lactation: a review of clinical data. Int J Environ Res Public Health. 2018;15:2241. 30322097 10.3390/ijerph15102241 PMC6210343

[87] Palacios C, Kostiuk LK, Pena-Rosas JP. Vitamin D supplementation for women during pregnancy. Cochrane Database Syst Rev. 2019;7:CD008873. 31348529 10.1002/14651858.CD008873.pub4 PMC6659840

[88] Roth DE, Morris SK, Zlotkin S, Gernand AD, Ahmed T, Shanta SS, et al. Vitamin D supplementation in pregnancy and lactation and infant growth. N Engl J Med. 2018;379:535–46. 30089075 10.1056/NEJMoa1800927 PMC6004541

[89] Pilz S, Hahn A, Schon C, Wilhelm M, Obeid R. Effect of two different multimicronutrient supplements on vitamin D status in women of childbearing age: a randomized trial. Nutrients. 2017;9:30. 28054964 10.3390/nu9010030 PMC5295074

[90] Meng X, Zhang J, Wan Q, Huang J, Han T, Qu T, Yu LL. Influence of vitamin D supplementation on reproductive outcomes of infertile patients: a systematic review and meta-analysis. Reprod Biol Endocrinol. 2023;21:17. 36737817 10.1186/s12958-023-01068-8 PMC9896710

[91] Institute of Medicine (US) Standing Committee on the Scientific Evaluation of Dietary Reference Intakes and its panel on folate, other B vitamins, and choline: Dietary reference intakes for thiamin, riboflavin, niacin, vitamin B(6), folate, vitamin B(12), pantothenic acid, biotin, and choline. Washington (DC): National Academies; 1998. 23193625

[92] EFSA Panel on Dietetic Products, Nutrition and Allergies. Dietary reference values for choline. EFSA J. 2016;14:e04484.

[93] Fischer LM, da Costa KA, Galanko J, Sha W, Stephenson B, Vick J, Zeisel SH. Choline intake and genetic polymorphisms influence choline metabolite concentrations in human breast milk and plasma. Am J Clin Nutr. 2010;92:336–46. 20534746 10.3945/ajcn.2010.29459 PMC2904035

[94] EFSA Panel on Nutrition, Novel Foods and Food Allergens, Turck D, Bohn T, Castenmiller J, De Henauw S, Hirsch-Ernst KI, et al. Choline and contribution to normal liver function of the foetus and exclusively breastfed infants: evaluation of a health claim pursuant to article 14 of regulation (EC) 1924/2006. EFSA J. 2023;21:e08115. 37502017 10.2903/j.efsa.2023.8115 PMC10369243

[95] Abdel Aziz R, Khalifa E, Talaat B, Abouahmad E, Hassan H. Maternal and neonatal benefits of prophylactic administration of vitamin K before elective cesarean section; a randomized control trial. Ann Neonatol. 2022;5:42–580.

[96] Shahrook S, Ota E, Hanada N, Sawada K, Mori R. Vitamin K supplementation during pregnancy for improving outcomes: a systematic review and meta-analysis. Sci Rep. 2018;8:11459. 30061633 10.1038/s41598-018-29616-y PMC6065418

[97] Creanga AA, Catalano PM, Bateman BT. Obesity in pregnancy. N Engl J Med. 2022;387:248–59. 35857661 10.1056/NEJMra1801040

[98] Yang Y, Cai Z, Zhang J. The effect of prepregnancy body mass index on maternal micronutrient status: a meta-analysis. Sci Rep. 2021;11:18100. 34518612 10.1038/s41598-021-97635-3 PMC8437962

[99] Jans G, Matthys C, Bogaerts A, Lannoo M, Verhaeghe J, Van der Schueren B, Devlieger R. Maternal micronutrient deficiencies and related adverse neonatal outcomes after bariatric surgery: a systematic review. Adv Nutr. 2015;6:420–9. 26178026 10.3945/an.114.008086 PMC4496736

[100] Shawe J, Ceulemans D, Akhter Z, Neff K, Hart K, Heslehurst N, et al. Pregnancy after bariatric surgery: Consensus recommendations for periconception, antenatal and postnatal care. Obes Rev. 2019;20:1507–22. 31419378 10.1111/obr.12927 PMC6852078

