# Supplementary material for: International expert consensus on micronutrient supplement use during the early life course
Source: BMC Pregnancy Childbirth. 2025 Jan 20;25:44. doi: 10.1186/s12884-024-07123-5 (PMC11744953; doi:10.1186/s12884-024-07123-5)
Supplement: Supplementary file 1 — Supplementary Material 1: Additional File 1 [file 12884_2024_7123_MOESM1_ESM.docx]

**Summary of micronutrient intake and supplement use recommendations during preconception, pregnancy and lactation in current national/regional guidelines**

**International Expert Consensus on Micronutrient Supplement Use During the Early Life Course**

Irene Cetin, Roland Devlieger, Erika Isolauri, Rima Obeid, Francesca Parisi, Stefan Pilz, Lenie van Rossem, [Maternal Nutrition Delphi Study Group], Régine Steegers-Theunissen

The table below summarises recommendations on supplement use (in **bold** **text**) and/or increased daily intake during preconception, pregnancy and lactation within current guidelines identified in the targeted literature review (TLR). Where timing is specified for the increased intake recommendations, these are indicated in parentheses.

If no increased intake recommendation was identified, or if intake recommendation does not differ from the recommendation for women who are not planning pregnancy, pregnant or lactating, then this is indicated with a dash.

| **Micronutrient (units*)** | **National guidelines** | | | | | | **International/regional guidelines** | | | |
| --- | --- | --- | --- | --- | --- | --- | --- | --- | --- | --- |
|  | **Australia [1-3]** | **D–A–CH [4-15]** | **Italy  [16, 17]** | **Poland/ Central Europe [18, 19]** | **UK [20-23]** | **USA [24-32]** | **WHO [33-35]** | **EFSA [4, 36-47]** | **FIGO [48]** | **Other (ATA [49, 50], Endocrine Society [51, 52])** |
| **Folate/folic acid (μg)**  Increased intake recommended? | Yes | Yes | Yes | Yes | Yes | Yes | Yes | Yes | Yes | No |
| *Preconception* | **≥400** | **400** | **400–800**  (≥8w) | **400  [▲800]**  (≥12w) | **400** | **400–800  [▲ 4 mg]**  (≥4w) | **400** | – | **400**  **[▲5 mg]** | – |
| *Pregnancy* | **≥400** (FT)  RDI 600 | **400**  (FT) | **400–800**  (FT)  RDI 600 | **400–800  [▲4 mg]**  (FT)  **600–800**  (ST&TT) | **400  [▲5 mg]**  (FT) | **400–800**  **[▲ 4 mg]**  (FT) | **400** | **NS**  AI 600 | **400–600** | – |
| *Lactation* | RDI 500 | – | AR 450  RDI 500 | **600–800** | – | **500** | – | AR 380 | **600** | – |
| **Vitamin B1 (mg)**  Increased intake recommended? | Yes | Yes | Yes | No | No | Yes | Yes | No | No | No |
| *Preconception* | – | – | – | – | – | – | – | – | – | – |
| *Pregnancy* | RDI 1.4 | RDI 1.2  (FT&ST)  RDI 1.3  (TT) | AR 1.2 | – | – | RDI 1.4 | AR 1.4 | – | – | – |
| *Lactation* | RDI 1.4 | RDI 1.3 | AR 1.2 | – | – | RDI 1.4 | AR 1.5 | – | – | – |
| **Vitamin B2 (mg)**  Increased intake recommended? | Yes | Yes | Yes | No | No | Yes | Yes | Yes | No | No |
| *Preconception* | – | – | – | – | – | – | – | – | – | – |
| *Pregnancy* | RDI 1.4 | RDI 1.3  (FT&ST)  RDI 1.4  (TT) | AR 1.4 | – | – | RDI 1.4 | AR 1.4 | AR 1.5 | – | – |
| *Lactation* | RDI 1.6 | RDI 1.4 | AR 1.5 | – | – | RDI 1.6 | AR 1.6 | AR 1.7 | – | – |
| **Vitamin B6 (mg)**  Increased intake recommended? | Yes | Yes | Yes | No | No | Yes | No | Yes | [Yes] | No |
| *Preconception* | – | – | – | – | – | – | – | – | **[Yes^\|\|^]** | – |
| *Pregnancy* | RDI 1.9 | RDI 1.5  (FT)  RDI 1.8  (ST&TT) | AR 1.6 | – | – | RDI 1.9 | – | AR 1.5 | **[Yes^\|\|^]** RDI 1.9 | – |
| *Lactation* | RDI 2.0 | RDI 1.6 | AR 1.7 | – | – | RDI 2.0 | – | AR 1.4 | **[Yes^\|\|^]** RDI 2.0 | – |
| **Vitamin B12 (μg)**  Increased intake recommended? | Yes | Yes | Yes | No | No | Yes | Yes | Yes | [Yes] | No |
| *Preconception* | – | – | – | – | – | – | – | – | **[Yes^\|\|^]** | – |
| *Pregnancy* | RDI 2.6 | RDI 4.5 | AR 2.2 | – | – | RDI 2.6 | RDI 2.6 | AI 4.5 | **[Yes^\|\|^]** RDI 2.6 | – |
| *Lactation* | RDI 2.8 | RDI 5.5 | AR 2.4 | – | – | RDI 2.8 | RDI 2.8 | AI 5 | **[Yes^\|\|^]** RDI 2.8 | – |
| **DHA (mg)**  Increased intake recommended? | No | Yes | Yes | Yes | No | No | No | Yes | No | No |
| *Preconception* | – | – | – | **[Yes^‡^]** | – | – | – | – | – | – |
| *Pregnancy* | – | **200^†^** RDI ≥200 | AI 200 | **200**  **[▲1000]** | – | – | – | **100–200** | – | – |
| *Lactation* | – | RDI ≥200 | AI 200 | – | – | – | – | **100–200** | – | – |
| **Iron (mg)**  Increased intake recommended? | Yes | Yes | Yes | [Yes] | [Yes] | Yes | Yes | No | [Yes] | No |
| *Preconception* | – | – | – | – | – | – | – | – | **[▲ 60^¶^]**  RDI 15–18 | – |
| *Pregnancy* | **[▲ ≤27]** RDI 27 | RDI 27 | AR 22 | **[Yes**]** | **[▲ 40–80^\|\|^]** | RDI 27 | **30–60** | – | **[Yes^\|\|^]** RDI 27 | – |
| *Lactation* | RDI 9  [▲ 10] | RDI 16^§^ | AR 8 | – | – | RDI 9 [▲ 10] | – | – | **[Yes^\|\|^]** RDI 9 | – |
| **Iodine (μg)**  Increased intake recommended? | Yes | Yes | Yes | Yes | No | Yes | Yes | Yes | [Yes] | Yes |
| *Preconception* | **150** | – | – | – | – | – | – | – | **[Yes^\|\|^]** RDI 150 | **150–200 [▲ ~400 mg/year^§§^]**  AI 250 |
| *Pregnancy* | **150** RDI 220 | **100–150**^††^  RDI 230^‡‡^ | AI 200 | **150–200** | – | RDI 220 | RDI 250 | AI 200 | **[Yes^\|\|^]** RDI 220 | **150–200 [▲ ~400 mg/year^§§^]**  AI 250 |
| *Lactation* | **150** RDI 270 | **100–150**^††^ RDI 260^‡‡^ | AI 200 | – | – | RDI 290 | RDI 250 | AI 200 | **[Yes^\|\|^]** RDI 290 | **150–200 [▲ ~400 mg/year^§§^]** AI 250 |
| **Calcium (mg)**  Increased intake recommended? | Yes | Yes | Yes | No | No | Yes | [Yes] | No | [Yes] | No |
| *Preconception* | – | – | – | – | – | – | – | – | **[Yes^\|\|^]** RDI 1000–1300 | – |
| *Pregnancy* | RDI 1000  [▲ 1300] | RDI 1000 [▲ 1200] | AR 1000 | – | – | RDI 1000  [▲ 1300] | **[1500–2000**^¶¶^**]** | – | **[Yes^\|\|^]** RDI 1000–1300 | – |
| *Lactation* | RDI 1000  [▲ 1300] | RDI 1000 [▲ 1200] | AR 800 | – | – | RDI 1000  [▲ 1300] | – | – | **[Yes^\|\|^]** RDI 1000–1300 | – |
| **Vitamin D (IU)**  Increased intake recommended? | Yes | No | Yes | Yes | No | No | No | No | Yes | Yes |
| *Preconception* | – | – | – | – | – | – | – | – | RDI ≥600 | – |
| *Pregnancy* | AI 200 | – | **600**  **[▲2000]** | **1500–2000 [▲4000]** | – | – | – | – | **[▲ ≥400**^\|\|^] RDI ≥600 | **≥1400** |
| *Lactation* | AI 200 | – | **600**  **[▲2000]** | **1500–2000 [▲4000]** | – | – | – | – | RDI ≥600 | **≥1400**  **[▲6000]** |
| **Choline (mg)**  Increased intake recommended? | Yes | No | No | No | No | Yes | No | Yes | [Yes] | No |
| *Preconception* | – | – | – | – | – | – | – | – | **[▲ ~400^\|\|^]**  AI 400–425 | – |
| *Pregnancy* | AI 440  [▼ 415] | – | – | – | – | AI 450 | – | AI 480 | **[▲ ~400^\|\|^]** AI 450 | – |
| *Lactation* | AI 550  [▼ 525] | – | – | – | – | AI 550 | – | AI 520 | – | – |
| **Vitamin K (μg)**  Increased intake recommended? | No | No | No | **No** | [Yes] | No | No | No | No | No |
| *Preconception* | – | – | – | – | – | – | – | – | – | – |
| *Pregnancy* | – | – | – | – | **[Yes^\|\|^]** | – | – | – | – | – |
| *Lactation* | – | – | – | – | – | – | – | – | – | – |
| **Selenium (μg)**  Increased intake recommended? | Yes | Yes | Yes | No | No | Yes | Yes | Yes | [Yes[ | No |
| *Preconception* | – | – | – | – | – | – | – | – | **[Yes^\|\|^]**  RDI 55 | – |
| *Pregnancy* | RDI 65 | – | AR 50 | – | – | RDI 60 | RDI 28 (ST) RDI 30 (TT) | – | **[Yes^\|\|^]** RDI 60 | – |
| *Lactation* | RDI 75 | AI 75 | AR 60 | – | – | RDI 70 | RDI 35 (0–6m PP) RDI 42 (7–12m PP) | AI 85 | **[Yes^\|\|^]** RDI 70 | – |

– No guideline recommendations identified by the TLR for increased intake versus non-pregnant, non-lactating women of the same age group.

[Yes] Supplement use/increased intake recommended for specific subgroups of women only.

▲ Higher dose [maximum] recommended for certain age groups or risk groups of women.

▼ Lower dose [minimum] recommended for certain age groups or risk groups of women.

* Unless other units specified in individual intake recommendation entries.

† Women who do not (regularly) eat fatty sea fish are recommended to supplement with DHA to reach the RDI.

‡ Women consuming small amounts of fish during the preconception period should consider an increased intake.

§ Applies to breastfeeding and non-breastfeeding women after giving birth.

|| Consider supplement use only in case of deficiency or other specific risk factors.

¶ In regions where anaemia is highly prevalent (>20% of women).

** For Poland, iron supplementation ≥30 mg recommended only in non-anemic women with ferritin levels over 60 μg/L after 16 weeks of pregnancy [53]. Women with non-anemic iron deficiency should receive 65 mg elemental iron daily. Women with iron-deficient anemia, an initial daily dose of 60–200 mg elemental iron is recommended.

†† The German Federal Institute of Risk Assessment (BfR) also recommends that women who are currently pregnant or breastfeeding should use a daily oral supplement containing 100–150 µg iodine [54].

‡‡ For Switzerland, the RDI is 200 μg.

§§ In low-resource countries/regions where neither salt iodisation nor daily iodine supplements are feasible, an annual dose can be used for pregnant women and women of childbearing age as a temporary measure to protect vulnerable populations.

¶¶ Supplement use recommended for all women in populations with low dietary calcium – but especially those at high risk of developing pre-eclampsia.

AI, adequate intake; AR, average requirement; ATA, American Thyroid Association; D–A–CH, Germany, Austria, Switzerland; DHA, docosahexaenoic acid; EFSA, European Food Safety Authority; FIGO, International Federation of Gynecology and Obstetrics; FT, first trimester; IU, international units; m, months; PP, post partum; RDI, recommended dietary intake; ST, second trimester; TLR, targeted literature review; TT, third trimester; w, weeks; WHO, World Health Organization.

**References**

1. National Health and Medical Research Council (Australia). Australian Dietary Guidelines. 2013. <https://www.eatforhealth.gov.au/sites/default/files/2022-09/n55_australian_dietary_guidelines.pdf>.

2. National Health and Medical Research Council (Australia). Nutrient reference values for Australia and New Zealand. 2017. <https://www.nhmrc.gov.au/sites/default/files/images/nutrient-refererence-dietary-intakes.pdf>.

3. Food Standards Australia New Zealand. Folic acid/folate and pregnancy. 2023. <https://www.foodstandards.gov.au/consumer/nutrition/pregnancy/folic-acidfolate-and-pregnancy>.

4. EFSA Panel on Dietetic Products, Nutrition and Allergies. Scientific opinion on dietary reference values for fats, including saturated fatty acids, polyunsaturated fatty acids, monounsaturated fatty acids, trans fatty acids, and cholesterol. EFSA Journal 2010;8:1461.

5. German Society for Nutrition. Recommendations for action - Nutrition during pregnancy. 2018. <https://www.dge.de/gesunde-ernaehrung/gezielte-ernaehrung/ernaehrung-in-schwangerschaft-und-stillzeit/handlungsempfehlungen-ernaehrung-in-der-schwangerschaft/>.

6. German Society for Nutrition. New reference values for vitamin D. Annals of nutrition & metabolism 2012;60:241-246.

7. German Society for Nutrition. New reference values for calcium. Annals of nutrition & metabolism 2013;63:186-192.

8. German Society for Nutrition. Iodine. <https://www.dge.de/wissenschaft/referenzwerte/jod/>.

9. German Society for Nutrition. Iron. <https://www.dge.de/wissenschaft/referenzwerte/eisen/>.

10. Jungert A, Linseisen J, Wagner KH, Richter M. Revised D-A-CH reference values for the intake of vitamin B6. Annals of nutrition & metabolism 2020;76:213-222.

11. Kipp AP, Strohm D, Brigelius-Flohé R, Schomburg L, Bechthold A, Leschik-Bonnet E, Heseker H. Revised reference values for selenium intake. J Trace Elem Med Biol 2015;32:195-199.

12. Ströhle A, Richter M, González-Gross M, Neuhäuser-Berthold M, Wagner KH, Leschik-Bonnet E, Egert S. The revised D-A-CH-reference values for the intake of vitamin B12: Prevention of deficiency and beyond. Mol Nutr Food Res 2019;63:e1801178.

13. Strohm D, Bechthold A, Isik N, Leschik-Bonnet E, Heseker H. Revised reference values for the intake of thiamin (vitamin B1), riboflavin (vitamin B2), and niacin. NFS Journal 2016;3:20-24.

14. German Society for Nutrition. Vitamin K. <https://www.dge.de/wissenschaft/referenzwerte/vitamin-k/>.

15. Krawinkel MB, Strohm D, Weissenborn A, Watzl B, Eichholzer M, Bärlocher K, et al. Revised D-A-CH intake recommendations for folate: how much is needed? Eur J Clin Nutr 2014;68:719-723.

16. Italian Society of Human Nutrition. LARN: dietary reference values of nutrients and energy for the Italian population. 2014. <https://www.dge.de/gesunde-ernaehrung/gezielte-ernaehrung/ernaehrung-in-schwangerschaft-und-stillzeit/handlungsempfehlungen-ernaehrung-in-der-schwangerschaft/>.

17. Saggese G, Vierucci F, Prodam F, Cardinale F, Cetin I, Chiappini E, et al. Vitamin D in pediatric age: consensus of the Italian Pediatric Society and the Italian Society of Preventive and Social Pediatrics, jointly with the Italian Federation of Pediatricians. Ital J Pediatr 2018;44:51.

18. Zimmer M, Sieroszewski P, Oszukowski P, Huras H, Fuchs T, Pawlosek A. Polish Society of Gynecologists and Obstetricians recommendations on supplementation during pregnancy. Ginekol Pol 2020;91:644-653.

19. Pludowski P, Karczmarewicz E, Bayer M, Carter G, Chlebna-Sokol D, Czech-Kowalska J, et al. Practical guidelines for the supplementation of vitamin D and the treatment of deficits in Central Europe - recommended vitamin D intakes in the general population and groups at risk of vitamin D deficiency. Endokrynol Pol 2013;64:319-327.

20. National Institute for Health and Care Excellence. Maternal and child nutrition. 2014. <https://www.nice.org.uk/guidance/ph11/resources/maternal-and-child-nutrition-pdf-1996171502533>.

21. National Institute for Health and Care Excellence. Vitamin D: supplement use in specific population groups. 2017. <https://www.nice.org.uk/guidance/ph56/resources/vitamin-d-supplement-use-in-specific-population-groups-pdf-1996421765317>.

22. Royal College of Obstetricians & Gynaecologists. Healthy eating and vitamin supplements in pregnancy. 2022. <https://www.rcog.org.uk/media/nkvpl2mn/healthy-eating-vitamin-supplements-pregnancy-patient-information.pdf>.

23. Pavord S, Daru J, Prasannan N, Robinson S, Stanworth S, Girling J, BSH Committee. UK guidelines on the management of iron deficiency in pregnancy. Br J Haematol 2020;188:819-830.

24. Institute of Medicine (US) Standing Committee on the Scientific Evaluation of Dietary Reference Intakes and its Panel on Folate, Other B Vitamins, and Choline: Dietary reference intakes for thiamin, riboflavin, niacin, vitamin B(6), folate, vitamin B(12), pantothenic acid, biotin, and choline. Washington (DC): National Academies Press; 1998.

25. Institute of Medicine Committee to Review Dietary Reference Intakes for Vitamin D., Calcium: The National Academies Collection: Reports funded by National Institutes of Health. In: *Dietary Reference Intakes for Calcium and Vitamin D.* edn. Edited by Ross AC, Taylor CL, Yaktine AL, Del Valle HB. Washington (DC): National Academies Press (US); 2011.

26. Institute of Medicine Panel on Micronutrients: Dietary reference intakes for vitamin A, vitamin K, arsenic, boron, chromium, copper, iodine, iron, manganese, molybdenum, nickel, silicon, vanadium, and zinc. Washington (DC): National Academies Press (US); 2001.

27. Institute of Medicine of the National Academies. Dietary reference intakes for energy, carbohydrate, fiber, fat, fatty acids, cholesterol, protein, and amino acids. 2005. <https://nap.nationalacademies.org/catalog/10490/dietary-reference-intakes-for-energy-carbohydrate-fiber-fat-fatty-acids-cholesterol-protein-and-amino-acids>.

28. Institute of Medicine of the National Academies. Dietary reference intakes: The essential guide to nutrient requirements. 2006. <https://nap.nationalacademies.org/catalog/11537/dietary-reference-intakes-the-essential-guide-to-nutrient-requirements>.

29. U.S. Department of Agriculture, U.S. Department of Health and Human Services. Dietary guidelines for Americans. 2020-2025. 2020. <https://www.dietaryguidelines.gov/sites/default/files/2021-03/Dietary_Guidelines_for_Americans-2020-2025.pdf>.

30. Office on Women's Health. Folic acid. 2021. <https://www.womenshealth.gov/a-z-topics/folic-acid>.

31. Centers for Disease Control and Prevention. Folic acid: Facts for clinicians. 2024. <https://www.cdc.gov/folic-acid/hcp/clinical-overview/index.html>.

32. U.S. Preventive Services Task Force. Folic acid supplementation to prevent neural tube defects: Preventive medication. 2023. <https://www.uspreventiveservicestaskforce.org/uspstf/recommendation/folic-acid-for-the-prevention-of-neural-tube-defects-preventive-medication>.

33. World Health Organization. WHO recommendations on antenatal care for a positive pregnancy experience. 2016. <https://iris.who.int/bitstream/handle/10665/250796/9789241549912-eng.pdf>.

34. World Health Organization. Universal salt iodization and sodium intake reduction: compatible, cost-effective strategies of great public health benefit. 2022. <https://iris.who.int/bitstream/handle/10665/361823/9789240053717-eng.pdf?sequence=1>.

35. World Health Organization. Reaching optimal iodine nutrition in pregnant and lactating women and young children. 2007. <https://cdn.who.int/media/docs/default-source/nutritionlibrary/reaching-optimal-iodine-nutrition-in-pregnant-and-lactating-women-and-young-children.pdf>.

36. EFSA Panel on Dietetic Products, Nutrition and Allergies. Scientific opinion on dietary reference values for iodine. EFSA Journal 2014;12:3660.

37. EFSA Panel on Dietetic Products, Nutrition and Allergies. Scientific opinion on dietary reference values for iron. EFSA Journal 2015;13:4254.

38. EFSA Panel on Dietetic Products, Nutrition and Allergies. Dietary reference values for choline. EFSA Journal 2016;14:e04484.

39. EFSA Panel on Dietetic Products, Nutrition and Allergies. Dietary reference values for vitamin D. EFSA Journal 2016;14:e04547.

40. EFSA Panel on Dietetic Products, Nutrition and Allergies. Scientific opinion on dietary reference values for calcium. EFSA Journal 2015;13:4101.

41. EFSA Panel on Dietetic Products, Nutrition and Allergies. Scientific opinion on dietary reference values for folate. EFSA Journal 2014;12:3893.

42. EFSA Panel on Dietetic Products, Nutrition and Allergies. Scientific opinion on dietary reference values for selenium. EFSA Journal 2014;12:3846.

43. EFSA Panel on Dietetic Products, Nutrition and Allergies. Dietary reference values for thiamin. EFSA Journal 2016;14:4653.

44. EFSA Panel on Dietetic Products, Nutrition and Allergies. Dietary reference values for riboflavin. EFSA Journal 2017;15:4919.

45. EFSA Panel on Dietetic Products, Nutrition and Allergies. Dietary reference values for vitamin B6. EFSA Journal 2016;14:4485.

46. EFSA Panel on Dietetic Products, Nutrition and Allergies. Scientific opinion on dietary reference values for cobalamin (vitamin B12). EFSA Journal 2015;13:4150.

47. EFSA Panel on Dietetic Products, Nutrition and Allergies. Dietary reference values for vitamin K. EFSA Journal 2017;15:4780.

48. Hanson MA, Bardsley A, De-Regil LM, Moore SE, Oken E, Poston L, et al. The International Federation of Gynecology and Obstetrics (FIGO) recommendations on adolescent, preconception, and maternal nutrition: "Think Nutrition First". International journal of gynaecology and obstetrics: the official organ of the International Federation of Gynaecology and Obstetrics 2015;131:S213-253.

49. Alexander EK, Pearce EN, Brent GA, Brown RS, Chen H, Dosiou C, et al. 2017 Guidelines of the American Thyroid Association for the Diagnosis and Management of Thyroid Disease During Pregnancy and the Postpartum. Thyroid 2017;27:315-389.

50. Becker DV, Braverman LE, Delange F, Dunn JT, Franklyn JA, Hollowell JG, et al. Iodine supplementation for pregnancy and lactation-United States and Canada: recommendations of the American Thyroid Association. Thyroid 2006;16:949-951.

51. Holick MF, Binkley NC, Bischoff-Ferrari HA, Gordon CM, Hanley DA, Heaney RP, et al. Evaluation, treatment, and prevention of vitamin D deficiency: An Endocrine Society clinical practice guideline. J Clin Endocrinol Metab 2011;96:1911-1930.

52. De Groot L, Abalovich M, Alexander EK, Amino N, Barbour L, Cobin RH, et al. Management of thyroid dysfunction during pregnancy and postpartum: an Endocrine Society clinical practice guideline. J Clin Endocrinol Metab 2012;97:2543-2565.

53. Sieroszewski P, Bomba-Opon D, Cnota W, Drosdzol-Cop A, Gogacz M, Grzesiak M, et al. Guidelines of the Polish Society of Gynecologists and Obstetricians on the diagnosis and treatment of iron deficiency and iron deficiency with anemia. Ginekol Pol 2023;94:415-422.

54. German Federal Institute for Risk Assessment. Iodine intake in Germany on the decline again - tips for a good iodine intake. 2021. <https://www.bfr.bund.de/cm/349/iodine-intake-in-germany-on-the-decline-again-tips-for-a-good-iodine-intake.pdf>.
