# Supplementary material for: International expert consensus on micronutrient supplement use during the early life course
Source: BMC Pregnancy Childbirth. 2025 Jan 20;25:44. doi: 10.1186/s12884-024-07123-5 (PMC11744953; doi:10.1186/s12884-024-07123-5)
Supplement: Supplementary file 2 — Supplementary Material 2: Additional File 2 [file 12884_2024_7123_MOESM2_ESM.docx]

**Supplementary Results**

**International Expert Consensus on Micronutrient Supplement Use During the Early Life Course**

Irene Cetin, Roland Devlieger, Erika Isolauri, Rima Obeid, Francesca Parisi, Stefan Pilz, Lenie van Rossem, [Maternal Nutrition Delphi Study Group], Régine Steegers-Theunissen

**Contents**

[**Supplementary Results** 2](#_Toc175845309)

[Supplementary Results Table S1. General considerations 2](#_Toc175845310)

[Supplementary Results Table S2. Micronutrient supplementation during preconception 6](#_Toc175845311)

[Supplementary Results Table S3. Micronutrient supplementation during the first trimester 9](#_Toc175845312)

[Supplementary Results Table S4. Micronutrient supplementation during the second trimester 11](#_Toc175845313)

[Supplementary Results Table S5. Micronutrient supplementation during the third trimester 13](#_Toc175845314)

[Supplementary Results Table S6. Micronutrient supplementation during lactation 15](#_Toc175845315)

[Supplementary Results Table S7. Considerations around specific micronutrients 18](#_Toc175845316)

[Supplementary Results Table S8. Risk and lifestyle-based approaches 20](#_Toc175845317)

# **Supplementary Results**

## Supplementary Results Table S1. General considerations

| **For each of the following stages of pregnancy, please indicate how important it is for healthcare professionals to provide recommendations on maternal diet:** | | | | | |
| --- | --- | --- | --- | --- | --- |
|  | **Survey 1** | | | | |
|  | **n*** | **Slightly not to not at all important (%)** | **Neutral (%)** | **Slightly to extremely important (%)** | **Median (IQR)** |
| Preconception/planning phase | 35 | 0 | 0 | 100 | 3 (1) |
| First trimester (Weeks 5–12) | 34 | 0 | 0 | 100 | 3 (1) |
| Second trimester (Weeks 13–27) | 34 | 0 | 0 | 100 | 2 (1) |
| Third trimester (Weeks 28–41) | 35 | 0 | 2.86 | 97.14 | 2 (1) |
| Postpartum/lactation | 35 | 0 | 2.86 | 97.14 | 2 (1) |
| **For each of the following stages of pregnancy, please indicate how important it is for healthcare professionals to provide recommendations on maternal dietary supplementation:** | | | | | |
| Preconception/planning phase | 34 | 0 | 0 | 100 | 3 (1) |
| First trimester (Weeks 5–12) | 33 | 0 | 0 | 100 | 3 (1) |
| Second trimester (Weeks 13–27) | 32 | 0 | 0 | 100 | 2 (1) |
| Third trimester (Weeks 28–41) | 33 | 0 | 0 | 100 | 2 (1) |
| Postpartum/lactation | 34 | 0 | 0 | 100 | 2 (1) |
| **Is there a need for increased awareness and education around maternal nutrition, including diet and dietary supplementation, for pregnant people and the general public? Please rate your level of agreement for each of the following stages of pregnancy:** | | | | | |
|  | **n*** | **Somewhat to strongly disagree (%)** | **Neither agree nor disagree (%)** | **Somewhat to strongly agree (%)** | **Median (IQR)** |
| Preconception/planning phase | 35 | 2.86 | 0.00 | 97.14 | 3 (0) |
| First trimester (Weeks 5–12) | 34 | 2.94 | 2.94 | 94.12 | 3 (1) |
| Second trimester (Weeks 13–27) | 34 | 2.94 | 0.00 | 97.06 | 3 (1) |
| Third trimester (Weeks 28–41) | 35 | 2.86 | 2.86 | 94.29 | 3 (1) |
| Postpartum/lactation | 34 | 2.94 | 0.00 | 97.06 | 3 (1) |
| **Is there a need for increased awareness and education around maternal nutrition, including diet and dietary supplementation, for healthcare professionals? Please rate your level of agreement for each of the following stages of pregnancy:** | | | | | |
| Preconception/planning phase | 33 | 3.03 | 3.03 | 93.94 | 3 (1) |
| First trimester (Weeks 5–12) | 33 | 3.03 | 0.00 | 96.97 | 3 (1) |
| Second trimester (Weeks 13–27) | 33 | 3.03 | 3.03 | 93.94 | 3 (1) |
| Third trimester (Weeks 28–41) | 34 | 2.94 | 2.94 | 94.12 | 3 (1) |
| Postpartum/lactation | 33 | 3.03 | 3.03 | 93.94 | 3 (1) |
| **To ensure optimal nutrient intake from preconception through to lactation, which diet(s) should a person who is hoping to get pregnant, who is currently pregnant, or who is lactating be advised to follow? Please rate your level of agreement with each of the following statements:** | | | | | |
|  | **n*** | **Somewhat to strongly disagree (%)** | **Neither agree nor disagree (%)** | **Somewhat to strongly agree (%)** | **Median (IQR)** |
| Specific diets should not be advised | 33 | 39.39 | 21.21 | 39.39 | 0 (2) |
| Mediterranean diet | 31 | 6.45 | 16.13 | 77.42 | 2 (1) |
| DASH (Dietary Approaches to Stop Hypertension) diet | 28 | 17.86 | 17.86 | 64.29 | 1 (2) |
| Regular national dietary guidelines | 31 | 6.45 | 9.68 | 83.87 | 2 (1) |
| **Please indicate how important you consider each of the following dietary supplementation approaches in achieving optimal nutrient intake from preconception through to lactation:** | | | | | |
|  | **n*** | **Slightly not to not at all important (%)** | **Neutral (%)** | **Slightly to extremely important (%)** | **Median (IQR)** |
| General multi-vitamin/multi-micronutrient supplementation (e.g. including folate, iron and other essential nutrients) | 34 | 5.88 | 14.70 | 79.41 | 2 (1) |
| Individual vitamin/micronutrient supplementation | 33 | 3.03 | 9.09 | 87.88 | 2 (1) |
| Approaches that are tailored to specific stages of pregnancy | 34 | 5.88 | 5.88 | 88.23 | 2 (1) |
| Approaches that are tailored to a person’s individual vitamin/micronutrient status (e.g. based on serum vitamin D and ferritin assessment) | 33 | 9.09 | 3.03 | 87.88 | 3 (1) |
| **How do you perceive the current guidelines for maternal nutrition, including diet and dietary supplementation? Please rate your level of agreement with each of the following statements:** | | | | | |
|  | **n*** | **Somewhat to strongly disagree (%)** | **Neither agree nor disagree (%)** | **Somewhat to strongly agree (%)** | **Median (IQR)** |
| Current guidelines are clear and consistent/harmonized across countries | 32 | **75.00** | 3.13 | 21.88 | -1 (1.25) |
| Current guidelines are clear and consistent/harmonized across different organizations | 30 | **73.33** | 10.00 | 16.67 | -1 (1.75) |
| Current guidelines are reflective of the latest evidence | 34 | 41.17 | 17.65 | 41.17 | 0 (2) |
| Current guidelines comprehensively cover all stages of pregnancy, from preconception through to lactation | 34 | 52.94 | 11.76 | 35.29 | -1 (3) |
| Current guidelines are easy to access | 33 | 39.39 | 21.21 | 39.39 | 0 (2) |
| Current guidelines are easy to follow | 31 | 38.71 | 19.35 | 41.94 | 0 (2) |

*n refers to the number of valid responses; ‘NA/unsure’ responses were considered invalid and excluded.

Figures in bold reached the threshold for consensus.

IQR, interquartile range; NA, not applicable.

## Supplementary Results Table S2. Micronutrient supplementation during preconception

| **To ensure optimal nutrient intake, when should a person who is hoping to get pregnant be advised to start dietary supplementation? Please rate your level of agreement with each of the following statements:** | | | | | | | | | | | | | | |
| --- | --- | --- | --- | --- | --- | --- | --- | --- | --- | --- | --- | --- | --- | --- |
|  | **Survey 1** | | | | | | | | | | | | | |
|  | **n*** | | **Somewhat to strongly disagree (%)** | | | **Neither agree nor disagree (%)** | | | **Somewhat to strongly agree (%)** | | | **Median (IQR)** | | |
| A balanced diet is sufficient; dietary supplementation should not be explicitly advised | 33 | | 75.75 | | | 9.09 | | | 15.15 | | | -1 (1) | | |
| Irrespective of when they hope to get pregnant, people of reproductive age who are not using contraception should be advised to start dietary supplementation | 33 | | 30.30 | | | 9.09 | | | 60.60 | | | 1 (3) | | |
| Up to 6 months before they hope to get pregnant | 32 | | 6.25 | | | 9.38 | | | 84.38 | | | 2 (1) | | |
| Up to 3 months before they hope to get pregnant | 32 | | 18.75 | | | 6.25 | | | 75.00 | | | 2 (1.25) | | |
| Up to 1 month before they hope to get pregnant | 32 | | 28.13 | | | 6.25 | | | 65.63 | | | 1.5 (3.25) | | |
| Once it has been confirmed that they are pregnant | 33 | | 36.36 | | | 6.06 | | | 57.57 | | | 2 (5) | | |
| **For each of the following, please indicate how important dietary supplementation is for a person who is hoping to get pregnant and at low risk of pregnancy complications:** | | | | | | | | | | | | | | |
|  | **Survey 1** | | | | | | | **Survey 2** | | | | | | |
|  | **n*** | **Slightly not to not at all important (%)** | | **Neutral (%)** | **Slightly to extremely important (%)** | | **Median (IQR)** | **n*** | | **Slightly not to not at all important (%)** | **Neutral (%)** | | **Slightly to extremely important (%)** | **Median (IQR)** |
| Vitamin B1 | 28 | 28.57 | | 32.14 | 39.29 | | 0 (2) | - | | - | - | | - | - |
| Vitamin B2 | 28 | 28.57 | | 32.14 | 39.29 | | 0 (2) | - | | - | - | | - | - |
| Vitamin B6 | 28 | 21.43 | | 32.14 | 46.43 | | 0 (1) | - | | - | - | | - | - |
| Vitamin B12 | 28 | 14.29 | | 28.57 | 57.14 | | 1 (2) | - | | - | - | | - | - |
| Vitamin D | 31 | 6.45 | | 19.35 | 74.19 | | 2 (2.5) | 31 | | 0.00 | 3.23 | | 96.77 | 2 (0.00) |
| Vitamin K | 27 | 33.33 | | 44.44 | 22.22 | | 0 (1) | - | | - | - | | - | - |
| Folic acid | 33 | 3.03 | | 0.00 | 96.97 | | 3 (0) | - | | - | - | | - | - |
| Choline | 27 | 22.22 | | 37.04 | 40.74 | | 0 (2) | - | | - | - | | - | - |
| Iodine | 27 | 14.81 | | 14.81 | 70.37 | | 2 (2.5) | 31 | | 3.23 | 9.68 | | 87.10 | 2 (1.00) |
| Magnesium | 28 | 25.00 | | 35.71 | 39.29 | | 0 (2.25) | - | | - | - | | - | - |
| Calcium | 27 | 14.81 | | 40.74 | 44.44 | | 0 (2) | - | | - | - | | - | - |
| Iron | 30 | 6.67 | | 23.33 | 70.00 | | 2 (2) | 32 | | 0.00 | 12.50 | | 87.50 | 2 (0.25) |
| Selenium | 28 | 21.43 | | 32.14 | 46.43 | | 0 (2) | - | | - | - | | - | - |
| DHA | 29 | 13.79 | | 24.14 | 62.07 | | 2 (2) | - | | - | - | | - | - |
|  | **For each of the following, please rate your perception of the strength/quality of evidence that supplementation during preconception for a person who is at low risk of complications improves pregnancy outcomes and/or fetal development:** | | | | | | | **Follow-up question for neutral or somewhat weak to very weak evidence ratings only: Please provide a reasoning for your answer** | | | | | | |
|  | **Survey 2** | | | | | | | **Survey 2** | | | | | | |
|  | **n*** | **Somewhat to very strong (%)** | | **Neutral (%)** | **Somewhat to very weak (%)** | | **Median (IQR)** | **n^†^** | | **Lack of evidence (%)** | **Discrepancies in existing evidence (%)** | | **Existing evidence is of low quality (%)** | **Other (%)** |
| Vitamin B1 | 23 | 13.04 | | 39.13 | 47.83 | | 0 (2) | 20 | | 80.00 | 10.00 | | 5.00 | 5.00 |
| Vitamin B2 | 24 | 12.50 | | 37.50 | 50.00 | | -0.5 (2) | 21 | | 76.19 | 4.76 | | 9.52 | 9.52 |
| Vitamin B6 | 24 | 29.17 | | 25.00 | 45.83 | | 0 (3) | 18 | | 66.67 | 5.56 | | 16.67 | 11.11 |
| Vitamin B12 | 25 | 44.00 | | 12.00 | 44.00 | | 0 (4) | 14 | | 42.86 | 7.14 | | 28.57 | 21.43 |
| Vitamin D | 31 | 67.74 | | 6.45 | 25.81 | | 1 (2.5) | 10 | | 40.00 | 20.00 | | 30.00 | 10.00 |
| Vitamin K | 26 | 7.69 | | 30.77 | 61.54 | | -1.5 (2) | 24 | | 75.00 | 4.17 | | 16.67 | 4.17 |
| Folic acid | 32 | 96.88 | | 3.13 | 0.00 | | 3 (1) | 1 | | 100.00 | 0.00 | | 0.00 | 0.00 |
| Choline | 27 | 14.81 | | 29.63 | 55.56 | | -1 (2) | 23 | | 69.57 | 8.70 | | 13.04 | 8.70 |
| Iodine | 31 | 87.10 | | 3.23 | 9.68 | | 1 (1) | 4 | | 75.00 | 25.00 | | 0.00 | 0.00 |
| Magnesium | 26 | 30.77 | | 26.92 | 42.31 | | 0 (2) | 18 | | 55.56 | 5.56 | | 33.33 | 5.56 |
| Calcium | 30 | 56.67 | | 10.00 | 33.33 | | 1 (2) | 13 | | 61.54 | 15.38 | | 15.38 | 7.69 |
| Iron | 31 | 70.97 | | 16.13 | 12.90 | | 1 (2) | 9 | | 44.44 | 33.33 | | 11.11 | 11.11 |
| Selenium | 26 | 23.08 | | 26.92 | 50.00 | | -0.5 (2) | 20 | | 75.00 | 5.00 | | 20.00 | 0.00 |
| DHA | 30 | 66.67 | | 10.00 | 23.33 | | 1 (2) | 11 | | 72.73 | 9.09 | | 9.09 | 9.09 |

*n refers to the number of valid responses; ‘NA/unsure’ responses were considered invalid and excluded.
^†^n refers to the number of valid responses from participants who rated the strength of evidence as neutral or somewhat weak to very weak.

DHA, docosahexaenoic acid; IQR, interquartile range; NA, not applicable.

## Supplementary Results Table S3. Micronutrient supplementation during the first trimester

| **For each of the following, please indicate how important dietary supplementation is during the first trimester for a person who is at low risk of pregnancy complications:** | | | | | | | | | | |
| --- | --- | --- | --- | --- | --- | --- | --- | --- | --- | --- |
|  | **Survey 1** | | | | | **Survey 2** | | | | |
|  | **n*** | **Slightly not to not at all important (%)** | **Neutral (%)** | **Slightly to extremely important (%)** | **Median (IQR)** | **n*** | **Slightly not to not at all important (%)** | **Neutral (%)** | **Slightly to extremely important (%)** | **Median (IQR)** |
| Vitamin B1 | 27 | 25.93 | 29.63 | 44.44 | 0 (2.5) | - | - | - | - | - |
| Vitamin B2 | 27 | 25.93 | 29.63 | 44.44 | 0 (2.5) | - | - | - | - | - |
| Vitamin B6 | 27 | 22.22 | 18.52 | 59.26 | 1 (2) | - | - | - | - | - |
| Vitamin B12 | 27 | 7.41 | 22.22 | 70.37 | 2 (2) | 30 | 13.33 | 16.67 | 70.00 | 1 (2) |
| Vitamin D | 31 | 3.23 | 9.68 | 87.10 | 2 (1) | - | - | - | - | - |
| Vitamin K | 26 | 26.92 | 38.46 | 34.62 | 0 (2.5) | - | - | - | - | - |
| Folic acid | 33 | 0.00 | 0.00 | 100.00 | 3 (1) | - | - | - | - | - |
| Choline | 25 | 16.00 | 36.00 | 48.00 | 0 (2) | - | - | - | - | - |
| Iodine | 28 | 3.57 | 17.86 | 78.57 | 2 (1.25) | - | - | - | - | - |
| Magnesium | 28 | 21.43 | 35.71 | 42.86 | 0 (2) | - | - | - | - | - |
| Calcium | 29 | 10.34 | 24.14 | 65.52 | 2 (2) | - | - | - | - | - |
| Iron | 31 | 6.45 | 19.35 | 74.19 | 2 (2.5) | 32 | 6.25 | 9.38 | 84.38 | 2 (1) |
| Selenium | 27 | 14.81 | 29.63 | 55.56 | 1 (2) | - | - | - | - | - |
| DHA | 28 | 7.14 | 17.86 | 75.00 | 2 (1.5) | - | - | - | - | - |
|  | **For each of the following, please rate your perception of the strength/quality of evidence that supplementation during the first trimester for a person who is at low risk of complications improves pregnancy outcomes and/or fetal development:** | | | | | **Follow-up question for neutral or somewhat weak to very weak evidence ratings only: Please provide a reasoning for your answer** | | | | |
|  | **Survey 2** | | | | | **Survey 2** | | | | |
|  | **n*** | **Somewhat to very strong (%)** | **Neutral (%)** | **Somewhat to very weak (%)** | **Median (IQR)** | **n^†^** | **Lack of evidence (%)** | **Discrepancies in existing evidence (%)** | **Existing evidence is of low quality (%)** | **Other (%)** |
| Vitamin B1 | 27 | 18.52 | 33.33 | 48.15 | 0 (2) | 22 | 77.27 | 4.55 | 13.64 | 4.55 |
| Vitamin B2 | 27 | 14.81 | 33.33 | 51.85 | -1 (2) | 24 | 75.00 | 8.33 | 8.33 | 8.33 |
| Vitamin B6 | 25 | 28.00 | 32.00 | 40.00 | 0 (3) | 18 | 66.67 | 5.56 | 27.78 | 0.00 |
| Vitamin B12 | 27 | 55.56 | 22.22 | 22.22 | 1 (1.5) | 12 | 66.67 | 16.67 | 16.67 | 0.00 |
| Vitamin D | 31 | 80.65 | 12.90 | 6.45 | 2 (2) | 6 | 33.33 | 50.0 | 16.67 | 0.00 |
| Vitamin K | 26 | 7.69 | 30.77 | 61.54 | -1 (2) | 24 | 75.00 | 8.33 | 16.67 | 0.00 |
| Folic acid | 31 | 100.00 | 0.00 | 0.00 | 3 (1) | - | - | - | - | - |
| Choline | 26 | 19.23 | 30.77 | 50.00 | -0.5 (2) | 21 | 71.43 | 0.00 | 23.81 | 4.76 |
| Iodine | 32 | 93.75 | 0.00 | 6.25 | 2 (1) | 3 | 33.33 | 33.33 | 33.33 | 0.00 |
| Magnesium | 30 | 33.33 | 23.33 | 43.33 | 0 (2.75) | 21 | 71.43 | 4.76 | 14.29 | 9.52 |
| Calcium | 31 | 38.71 | 22.58 | 38.71 | 0 (2) | 20 | 70.00 | 15.00 | 0.00 | 15.00 |
| Iron | 32 | 62.50 | 28.13 | 9.38 | 1 (2) | 12 | 33.33 | 41.67 | 0.00 | 25.00 |
| Selenium | 27 | 18.52 | 33.33 | 48.15 | 0 (2) | 22 | 72.73 | 9.09 | 18.18 | 0.00 |
| DHA | 31 | 70.97 | 22.58 | 6.45 | 1 (2) | 10 | 60.00 | 30.00 | 0.00 | 10.00 |

*n refers to the number of valid responses; ‘NA/unsure’ responses were considered invalid and excluded.
^†^n refers to the number of valid responses from participants who rated the strength of evidence as neutral or somewhat weak to very weak.
DHA, docosahexaenoic acid; IQR, interquartile range; NA, not applicable.

## Supplementary Results Table S4. Micronutrient supplementation during the second trimester

| **For each of the following, please indicate how important dietary supplementation is during the second trimester for a person who is at low risk of pregnancy complications:** | | | | | | | | | | |
| --- | --- | --- | --- | --- | --- | --- | --- | --- | --- | --- |
|  | **Survey 1** | | | | | **Survey 2** | | | | |
|  | **n*** | **Slightly not to not at all important (%)** | **Neutral (%)** | **Slightly to extremely important (%)** | **Median (IQR)** | **n*** | **Slightly not to not at all important (%)** | **Neutral (%)** | **Slightly to extremely important (%)** | **Median (IQR)** |
| Vitamin B1 | 27 | 22.22 | 25.93 | 51.85 | 1 (2) | - | - | - | - | - |
| Vitamin B2 | 27 | 22.22 | 25.93 | 51.85 | 1 (2) | - | - | - | - | - |
| Vitamin B6 | 27 | 22.22 | 18.52 | 59.26 | 2 (2) | - | - | - | - | - |
| Vitamin B12 | 27 | 7.41 | 22.22 | 70.37 | 2 (2) | 29 | 13.79 | 20.69 | 65.52 | 1 (2) |
| Vitamin D | 31 | 3.23 | 6.45 | 90.32 | 2 (1) | - | - | - | - | - |
| Vitamin K | 26 | 26.92 | 34.62 | 38.46 | 0 (2.75) | - | - | - | - | - |
| Folic acid | 32 | 12.50 | 15.63 | 71.88 | 2 (2) | 31 | 3.23 | 9.68 | 87.10 | 2 (1) |
| Choline | 25 | 12.00 | 36.00 | 52.00 | 1 (2) | - | - | - | - | - |
| Iodine | 28 | 3.57 | 17.86 | 78.57 | 2 (1) | - | - | - | - | - |
| Magnesium | 27 | 22.22 | 22.22 | 55.56 | 2 (2) | - | - | - | - | - |
| Calcium | 29 | 10.34 | 20.69 | 68.97 | 2 (2) | - | - | - | - | - |
| Iron | 31 | 6.45 | 16.13 | 77.42 | 2 (2) | - | - | - | - | - |
| Selenium | 27 | 14.81 | 29.36 | 55.56 | 2 (2) | - | - | - | - | - |
| DHA | 28 | 7.14 | 14.29 | 78.57 | 2 (1) | - | - | - | - | - |
|  | **For each of the following, please rate your perception of the strength/quality of evidence that supplementation during the second trimester for a person who is at low risk of complications improves pregnancy outcomes and/or fetal development:** | | | | | **Follow-up question for neutral or somewhat weak to very weak evidence ratings only: Please provide a reasoning for your answer** | | | | |
|  | **Survey 2** | | | | | **Survey 2** | | | | |
|  | **n*** | **Somewhat to very strong (%)** | **Neutral (%)** | **Somewhat to very weak (%)** | **Median (IQR)** | **n^†^** | **Lack of evidence (%)** | **Discrepancies in existing evidence (%)** | **Existing evidence is of low quality (%)** | **Other (%)** |
| Vitamin B1 | 26 | 23.08 | 26.92 | 50.00 | -0.5 (2) | 20 | 85.00 | 0.00 | 10.00 | 5.00 |
| Vitamin B2 | 25 | 24.00 | 24.00 | 52.00 | -1 (2) | 19 | 78.95 | 5.26 | 10.53 | 5.26 |
| Vitamin B6 | 24 | 33.33 | 25.00 | 41.67 | 0 (3) | 16 | 81.25 | 6.25 | 12.5 | 0.00 |
| Vitamin B12 | 28 | 60.71 | 10.71 | 28.57 | 1 (2.25) | 11 | 90.91 | 0.00 | 0.00 | 9.09 |
| Vitamin D | 30 | 86.67 | 3.33 | 10.00 | 2 (2) | 4 | 25.00 | 50.00 | 25.00 | 0.00 |
| Vitamin K | 26 | 3.85 | 38.46 | 57.69 | -1.5 (2) | 25 | 80.00 | 8.00 | 12.00 | 0.00 |
| Folic acid | 30 | 76.67 | 6.67 | 16.67 | 2 (1) | 7 | 28.57 | 42.86 | 0.00 | 28.57 |
| Choline | 26 | 30.77 | 23.08 | 46.15 | 0 (3) | 18 | 77.78 | 0.00 | 16.67 | 5.56 |
| Iodine | 32 | 87.5 | 3.13 | 9.38 | 1 (1) | 4 | 0.00 | 75.00 | 0.00 | 25.00 |
| Magnesium | 29 | 41.38 | 17.24 | 41.38 | 0 (2) | 17 | 64.71 | 11.76 | 17.65 | 5.88 |
| Calcium | 31 | 51.61 | 16.13 | 32.26 | 1 (2) | 15 | 86.67 | 0.00 | 6.67 | 6.67 |
| Iron | 32 | 81.25 | 15.63 | 3.13 | 2 (2) | 6 | 16.67 | 50.00 | 0.00 | 33.33 |
| Selenium | 28 | 17.86 | 28.57 | 53.57 | -1 (2) | 23 | 65.22 | 17.39 | 17.39 | 0.00 |
| DHA | 31 | 87.10 | 6.45 | 6.45 | 2 (1) | 5 | 40.00 | 60.00 | 0.00 | 0.00 |

*n refers to the number of valid responses; ‘NA/unsure’ responses were considered invalid and excluded.

^†^n refers to the number of valid responses from participants who rated the strength of evidence as neutral or somewhat weak to very weak.

DHA, docosahexaenoic acid; IQR, interquartile range; NA, not applicable.

## Supplementary Results Table S5. Micronutrient supplementation during the third trimester

| **For each of the following, please indicate how important dietary supplementation is during the third trimester for a person who is at low risk of pregnancy complications:** | | | | | | | | | | |
| --- | --- | --- | --- | --- | --- | --- | --- | --- | --- | --- |
|  | **Survey 1** | | | | | **Survey 2** | | | | |
|  | **n*** | **Slightly not to not at all important (%)** | **Neutral (%)** | **Slightly to extremely important (%)** | **Median (IQR)** | **n*** | **Slightly not to not at all important (%)** | **Neutral (%)** | **Slightly to extremely important (%)** | **Median (IQR)** |
| Vitamin B1 | 27 | 18.52 | 25.93 | 55.56 | 1 (2) | - | - | - | - | - |
| Vitamin B2 | 27 | 18.52 | 25.93 | 55.56 | 1 (2) | - | - | - | - | - |
| Vitamin B6 | 27 | 18.52 | 22.22 | 59.26 | 1(2) | - | - | - | - | - |
| Vitamin B12 | 27 | 7.41 | 22.22 | 70.37 | 2 (2) | 30 | 10.00 | 23.33 | 66.67 | 1 (2) |
| Vitamin D | 32 | 3.13 | 6.25 | 90.63 | 2 (1) | - | - | - | - | - |
| Vitamin K | 26 | 23.08 | 26.92 | 50.00 | 0.5 (2) | - | - | - | - | - |
| Folic acid | 32 | 15.63 | 21.88 | 62.50 | 2 (2) | - | - | - | - | - |
| Choline | 25 | 12.00 | 36.00 | 52.00 | 1 (2) | - | - | - | - | - |
| Iodine | 28 | 3.57 | 25.00 | 71.43 | 2 (2) | 32 | 9.38 | 15.63 | 75.00 | 2 (1.25) |
| Magnesium | 28 | 21.43 | 25.00 | 53.57 | 2 (2) | - | - | - | - | - |
| Calcium | 29 | 10.34 | 17.24 | 72.41 | 2 (2) | 31 | 9.68 | 29.03 | 61.29 | 1 (2) |
| Iron | 32 | 6.25 | 12.50 | 81.25 | 2 (1.25) | - | - | - | - | - |
| Selenium | 27 | 14.81 | 25.93 | 59.26 | 2 (2) | - | - | - | - | - |
| DHA | 28 | 7.14 | 17.86 | 75.00 | 2 (1.5) | - | - | - | - | - |
|  | **For each of the following, please rate your perception of the strength/quality of evidence that supplementation during the third trimester for a person who is at low risk of complications improves pregnancy outcomes and/or fetal development:** | | | | | **Follow-up question for neutral or somewhat weak to very weak evidence ratings only: Please provide a reasoning for your answer** | | | | |
|  | **Survey 2** | | | | | **Survey 2** | | | | |
|  | **n*** | **Somewhat to very strong (%)** | **Neutral (%)** | **Somewhat to very weak (%)** | **Median (IQR)** | **n^†^** | **Lack of evidence (%)** | **Discrepancies in existing evidence (%)** | **Existing evidence is of low quality (%)** | **Other (%)** |
| Vitamin B1 | 25 | 24.00 | 24.00 | 52.00 | -1 (2) | 19 | 89.47 | 0.00 | 10.53 | 0.00 |
| Vitamin B2 | 26 | 26.92 | 19.23 | 53.85 | -1 (2.75) | 19 | 89.47 | 0.00 | 10.53 | 0.00 |
| Vitamin B6 | 25 | 32.00 | 20.00 | 48.00 | 0 (2) | 17 | 76.47 | 5.88 | 17.65 | 0.00 |
| Vitamin B12 | 28 | 60.71 | 17.86 | 21.43 | 1 (1) | 11 | 63.64 | 9.09 | 27.27 | 0.00 |
| Vitamin D | 31 | 90.32 | 3.23 | 6.45 | 2 (1) | 3 | 33.33 | 33.33 | 33.33 | 0.00 |
| Vitamin K | 26 | 7.69 | 38.46 | 53.85 | -1 (2) | 24 | 75.00 | 0.00 | 25.00 | 0.00 |
| Folic acid | 32 | 53.13 | 25.00 | 21.88 | 1 (2) | 15 | 66.67 | 13.33 | 13.33 | 6.67 |
| Choline | 27 | 37.04 | 22.22 | 40.74 | 0 (3) | 17 | 88.24 | 0.00 | 11.76 | 0.00 |
| Iodine | 32 | 87.50 | 6.25 | 6.25 | 1 (1) | 5 | 20.00 | 60.00 | 20.00 | 0.00 |
| Magnesium | 30 | 43.33 | 30.00 | 26.67 | 0 (1.75) | 17 | 58.82 | 23.53 | 17.65 | 0.00 |
| Calcium | 30 | 66.67 | 23.33 | 10.00 | 1 (2) | 10 | 80.00 | 20.00 | 0.00 | 0.00 |
| Iron | 32 | 87.50 | 9.38 | 3.13 | 2 (1.25) | 4 | 25.00 | 50.00 | 0.00 | 25.00 |
| Selenium | 28 | 14.29 | 28.57 | 57.14 | -1 (2) | 24 | 75.00 | 4.17 | 20.83 | 0.00 |
| DHA | 31 | 87.10 | 3.23 | 9.68 | 2 (1) | 5 | 40.00 | 40.00 | 20.00 | 0.00 |

*n refers to the number of valid responses; ‘NA/unsure’ responses were considered invalid and excluded.

^†^n refers to the number of valid responses from participants who rated the strength of evidence as neutral or somewhat weak to very weak.

DHA, docosahexaenoic acid; IQR, interquartile range; NA, not applicable.

## Supplementary Results Table S6. Micronutrient supplementation during lactation

| **For each of the following, please indicate how important dietary supplementation is during lactation for a person who is at low risk of pregnancy complications:** | | | | | | | | | | | | | | |
| --- | --- | --- | --- | --- | --- | --- | --- | --- | --- | --- | --- | --- | --- | --- |
|  | **Survey 1** | | | | | | | **Survey 2** | | | | | | |
|  | **n*** | **Slightly not to not at all important (%)** | **Neutral (%)** | | **Slightly to extremely important (%)** | **Median (IQR)** | | **n*** | **Slightly not to not at all important (%)** | | **Neutral (%)** | **Slightly to extremely important (%)** | | **Median (IQR)** |
| Vitamin B1 | 28 | 25.00 | 21.43 | | 53.57 | 1 (2.25) | | - | - | | - | - | | - |
| Vitamin B2 | 28 | 25.00 | 21.43 | | 53.57 | 1 (2.25) | | - | - | | - | - | | - |
| Vitamin B6 | 28 | 25.00 | 21.43 | | 53.57 | 1 (2.25) | | - | - | | - | - | | - |
| Vitamin B12 | 28 | 10.71 | 25.00 | | 64.29 | 1.5 (2) | | - | - | | - | - | | - |
| Vitamin D | 32 | 3.13 | 12.50 | | 84.38 | 2 (1.25) | | - | - | | - | - | | - |
| Vitamin K | 28 | 28.57 | 25.00 | | 46.43 | 0 (3) | | - | - | | - | - | | - |
| Folic acid | 29 | 17.24 | 17.24 | | 65.52 | 2 (2) | | - | - | | - | - | | - |
| Choline | 26 | 15.38 | 30.77 | | 53.85 | 1 (2) | | - | - | | - | - | | - |
| Iodine | 28 | 7.14 | 28.57 | | 64.29 | 1.5 (2) | | - | - | | - | - | | - |
| Magnesium | 28 | 21.43 | 17.86 | | 60.71 | 2 (2) | | - | - | | - | - | | - |
| Calcium | 31 | 9.68 | 16.13 | | 74.19 | 2 (2) | | 30 | 3.33 | | 6.67 | 90.00 | | 2 (0.75) |
| Iron | 31 | 6.45 | 22.58 | | 70.97 | 2 (2.5) | | 30 | 6.67 | | 6.67 | 86.67 | | 2 (1) |
| Selenium | 28 | 17.86 | 21.43 | | 60.71 | 2 (2) | | - | - | | - | - | | - |
| DHA | 27 | 7.41 | 14.81 | | 77.78 | 2 (1) | | - | - | | - | - | | - |
|  | **For each of the following, please rate your perception of the strength/quality of evidence that supplementation during lactation for a person who has experienced a low-risk pregnancy improves maternal health and/or infant development:** | | | | | | | **Follow-up question for neutral or somewhat weak to very weak evidence ratings only: Please provide a reasoning for your answer** | | | | | | |
|  | **Survey 2** | | | | | | | **Survey 2** | | | | | | |
|  | **n*** | **Somewhat to very strong (%)** | **Neutral (%)** | | **Somewhat to very weak (%)** | **Median (IQR)** | | **n^†^** | **Lack of evidence (%)** | | **Discrepancies in existing evidence (%)** | **Existing evidence is of low quality (%)** | | **Other (%)** |
| Vitamin B1 | 26 | 23.08 | 15.38 | | 61.54 | -1 (2) | | 20 | 100.00 | | 0.00 | 0.00 | | 0.00 |
| Vitamin B2 | 27 | 25.93 | 14.81 | | 59.26 | -1 (2.5) | | 20 | 100.00 | | 0.00 | 0.00 | | 0.00 |
| Vitamin B6 | 26 | 26.92 | 19.23 | | 53.85 | -1 (2.75) | | 19 | 89.47 | | 5.26 | 5.26 | | 0.00 |
| Vitamin B12 | 28 | 53.57 | 14.29 | | 32.14 | 1 (2.25) | | 13 | 92.31 | | 7.69 | 0.00 | | 0.00 |
| Vitamin D | 30 | 90.00 | 3.33 | | 6.67 | 2 (1) | | 3 | 100.00 | | 0.00 | 0.00 | | 0.00 |
| Vitamin K | 26 | 11.54 | 26.92 | | 61.54 | -1 (2) | | 23 | 82.61 | | 13.04 | 4.35 | | 0.00 |
| Folic acid | 31 | 29.03 | 32.26 | | 38.71 | 0 (2) | | 22 | 86.36 | | 4.55 | 9.09 | | 0.00 |
| Choline | 27 | 18.52 | 29.63 | | 51.85 | -1 (2) | | 22 | 90.91 | | 0.00 | 9.09 | | 0.00 |
| Iodine | 31 | 67.74 | 16.13 | | 16.13 | 1 (1) | | 10 | 90.00 | | 10.00 | 0.00 | | 0.00 |
| Magnesium | 29 | 27.59 | 31.03 | | 41.38 | 0 (2) | | 21 | 90.48 | | 9.52 | 0.00 | | 0.00 |
| Calcium | 31 | 87.10 | 3.23 | | 9.68 | 1 (1) | | 4 | 75.00 | | 25.00 | 0.00 | | 0.00 |
| Iron | 31 | 80.65 | 6.45 | | 12.90 | 1 (1) | | 6 | 83.33 | | 16.67 | 0.00 | | 0.00 |
| Selenium | 28 | 14.29 | 32.14 | | 53.57 | -1 (2) | | 24 | 91.67 | | 0.00 | 8.33 | | 0.00 |
| DHA | 30 | 90.00 | 3.33 | | 6.67 | 1 (1) | | 3 | 66.67 | | 33.33 | 0.00 | | 0.00 |
| **To ensure optimal nutrient intake postpartum, should a person who is not breastfeeding be advised to continue dietary supplementation? If so, for how long? Please rate your level of agreement with each of the following statements:** | | | | | | | | | | | | | | |
|  | **Survey 1** | | | | | | | | | | | | | |
|  | **n*** | | | **Somewhat to strongly disagree (%)** | | | **Neither agree nor disagree (%)** | | | **Somewhat to strongly agree (%)** | | | **Median (IQR)** | |
| Continuing dietary supplementation should not be advised | 31 | | | 54.84 | | | 25.81 | | | 19.35 | | | -1 (2) | |
| Longer than 6 months after birth | 28 | | | 39.29 | | | 25.00 | | | 35.71 | | | 0 (3) | |
| Up to 6 months after birth | 27 | | | 37.04 | | | 18.52 | | | 44.44 | | | 0 (3.5) | |
| Up to 3 months after birth | 27 | | | 29.63 | | | 22.22 | | | 48.15 | | | 0 (3.5 | |
| Up to 1 month after birth | 27 | | | 29.63 | | | 22.22 | | | 48.15 | | | 0 (2.5) | |
| **To ensure optimal nutrient intake following lactation, should a person be advised to continue dietary supplementation after weaning? If so, for how long? Please rate your level of agreement with each of the following statements:** | | | | | | | | | | | | | | |
| Continuing dietary supplementation should not be advised | 31 | | | 45.16 | | | 32.26 | | | 22.58 | | | 0 (2) | |
| Up to 6 months after weaning | 27 | | | 33.33 | | | 25.93 | | | 40.74 | | | 0 (2.5) | |
| Up to 3 months after weaning | 27 | | | 25.93 | | | 25.93 | | | 48.15 | | | 0 (2.5) | |
| Up to 1 month after weaning | 27 | | | 37.04 | | | 29.63 | | | 33.33 | | | 0 (2.5) | |

*n refers to the number of valid responses; ‘NA/unsure’ responses were considered invalid and excluded.

^†^n refers to the number of valid responses from participants who rated the strength of evidence as neutral or somewhat weak to very weak.

DHA, docosahexaenoic acid; IQR, interquartile range; NA, not applicable.

## Supplementary Results Table S7. Considerations around specific micronutrients

| **To ensure optimal nutrient intake from preconception through to lactation, please indicate your level of agreement with the following micronutrient-specific considerations:** | | | | | |
| --- | --- | --- | --- | --- | --- |
|  | **Survey 1** | | | | |
|  | **n*** | **Somewhat to strongly disagree (%)** | **Neither agree nor disagree (%)** | **Somewhat to strongly agree (%)** | **Median  (IQR)** |
| For folate supplementation, food fortification is the optimal strategy | 31 | 48.39 | 12.90 | 38.71 | 0 (2.5) |
| For folate supplementation, food fortification is insufficient to meet the needs of a person who is hoping to get pregnant, who is currently pregnant, or who is lactating | 30 | 6.66 | 13.33 | **80.00** | 1 (1) |
| For folate supplementation, 5-MTHF should be used instead of folic acid | 21 | 14.29 | 23.81 | 61.90 | 1 (1) |
| For iron supplementation, the type of salt that is used is important | 24 | 20.83 | 4.17 | **75.00** | 2 (1.25) |
| For vitamin D supplementation, it is important to follow an individualized rather than ‘one-fits-all’ approach | 32 | 28.13 | 3.13 | 68.75 | 1.5 (3) |
| For iodine supplementation, it is important to follow an individualized rather than ‘one-fits-all’ approach | 32 | 25.00 | 3.13 | **71.88** | 2 (2.25) |
| Optimal levels of DHA should be achieved through supplementation rather than regular intake of fish | 31 | 41.94 | 22.58 | 35.48 | 0 (2.5) |

| **For each of the following, please indicate how concerned you are about the tolerable upper intake^†^ level being exceeded?** | | | | | |
| --- | --- | --- | --- | --- | --- |
|  | **Survey 1** | | | | |
|  | **n*** | **Slightly unconcerned to not at all concerned (%)** | **Neutral  (%)** | **Slightly concerned to extremely concerned (%)** | **Median**  **(IQR)** |
| Vitamin B6 | 30 | 53.33 | 13.33 | 33.33 | -2 (3) |
| Vitamin B12 | 30 | 60.00 | 16.66 | 23.33 | -2 (2) |
| Folic acid/folate | 32 | 34.38 | 12.50 | 53.13 | 1 (3.25) |
| Iron | 33 | 24.24 | 12.12 | 63.64 | 1 (2) |
| Iodine | 30 | 13.33 | 20.00 | 66.67 | 1 (2) |

*n refers to the number of valid responses; ‘NA/unsure’ responses were considered invalid and excluded.

^†^The tolerable upper intake level refers to the maximum amount that can be consumed daily that is not expected to pose a health risk.

Figures in bold reached the threshold for consensus.

5-MTHF, 5-methyltetrahydrofolate (5-MTHF); DHA, docosahexaenoic acid; IQR, interquartile range; NA, not applicable.

## Supplementary Results Table S8. Risk and lifestyle-based approaches

| **From preconception through to lactation, should dietary supplementation recommendations be personalized or tailored? Please rate your level of agreement with the following statements:** | | | | | |
| --- | --- | --- | --- | --- | --- |
|  | **Survey 1** | | | | |
|  | **n*** | **Somewhat to strongly disagree (%)** | **Neither agree nor disagree (%)** | **Somewhat to strongly agree (%)** | **Median (IQR)** |
| The recommendations are universally applicable and do not need to be tailored or personalized | 32 | 84.38 | 3.13 | 12.50 | -2 (1) |
| The recommendations should be personalized | 32 | 9.38 | 3.13 | 87.50 | 2 (1) |
| The recommendations should be tailored to a person’s lifestyle | 32 | 3.13 | 6.25 | 90.63 | 2 (2) |
| The recommendations should be tailored to a person’s medical conditions | 32 | 0.00 | 0.00 | 100.00 | 3 (1) |
| The recommendations should be tailored to a person’s history of pregnancy and birth complications | 32 | 6.25 | 0.00 | 93.75 | 3 (2) |
| **From preconception through to lactation, please indicate how important it is to have tailored approaches to diet and/or dietary supplementation for the following groups:** | | | | | |
|  | **Survey 1** | | | | |
|  | **n*** | **Slightly not to not at all important (%)** | **Neutral (%)** | **Slightly to extremely important (%)** | **Median (IQR)** |
| People who are athletes or highly active | 29 | 6.90 | 6.90 | 86.21 | 2 (2) |
| People who are following a vegan diet | 32 | 0.00 | 0.00 | 100.00 | 3 (0) |
| People who are following a vegetarian diet | 32 | 0.00 | 0.00 | 100.00 | 2 (1) |
| People with celiac disease | 31 | 6.45 | 0.00 | 93.55 | 3 (1) |
| People with obesity | 32 | 0.00 | 6.25 | 93.75 | 2 (1) |
| People with polycystic ovary syndrome (PCOS) | 28 | 3.57 | 7.14 | 89.29 | 2 (1) |
| People with diabetes | 31 | 0.00 | 0.00 | 100.00 | 3 (0) |
| People with a history of pregnancy and birth complications (e.g. history of previous NTDs, or those at risk of congenital malformations, pre-term births, pre-eclampsia, gestational diabetes) | 32 | 0.00 | 0.00 | 100.00 | 3 (0) |
| **For each of the following, please indicate how important it is to adjust the dietary supplementation recommendations for an athlete or highly active person who is hoping to get pregnant, who is currently pregnant, or who is lactating:** | | | | | |
|  | **Survey 1** | | | | |
|  | **n*** | **Slightly not to not at all important (%)** | **Neutral (%)** | **Slightly to extremely important (%)** | **Median (IQR)** |
| Vitamin B1 | 21 | 23.81 | 14.29 | 61.90 | 1 (2) |
| Vitamin B2 | 21 | 23.81 | 14.29 | 61.90 | 1 (2) |
| Vitamin B6 | 21 | 23.81 | 9.52 | 66.67 | 1 (2) |
| Vitamin B12 | 21 | 14.29 | 9.52 | 76.19 | 2 (1) |
| Vitamin D | 22 | 13.64 | 9.09 | 77.27 | 2 (1.75) |
| Vitamin K | 20 | 25.00 | 25.00 | 50.00 | 0.5 (2.5) |
| Folic acid | 24 | 20.83 | 4.17 | 75.00 | 2 (2.25) |
| Choline | 21 | 19.05 | 9.52 | 71.43 | 2 (2) |
| Iodine | 22 | 18.18 | 0.00 | 81.82 | 2 (2) |
| Magnesium | 21 | 19.05 | 9.52 | 71.43 | 2 (3) |
| Calcium | 23 | 13.04 | 8.70 | 78.26 | 2 (1.5) |
| Iron | 24 | 16.67 | 0.00 | 83.33 | 2 (1.25) |
| Selenium | 21 | 19.05 | 9.52 | 71.43 | 1 (2) |
| DHA | 22 | 13.64 | 0.00 | 86.36 | 2 (2) |
| **For each of the following, please indicate how important it is to adjust the dietary supplementation recommendations for a person following a vegetarian diet who is hoping to get pregnant, who is currently pregnant, or who is lactating:** | | | | | |
|  | **Survey 1** | | | | |
|  | **n*** | **Slightly not to not at all important (%)** | **Neutral (%)** | **Slightly to extremely important (%)** | **Median (IQR)** |
| Vitamin B1 | 25 | 24.00 | 16.00 | 60.00 | 1 (2) |
| Vitamin B2 | 25 | 20.00 | 16.00 | 64.00 | 1 (2) |
| Vitamin B6 | 25 | 20.00 | 16.00 | 64.00 | 1 (2) |
| Vitamin B12 | 30 | 0.00 | 6.67 | 93.33 | 2 (1) |
| Vitamin D | 28 | 0.00 | 10.71 | 89.29 | 2 (1) |
| Vitamin K | 24 | 20.83 | 33.33 | 45.83 | 0 (2) |
| Folic acid | 29 | 24.14 | 6.90 | 68.97 | 2 (2) |
| Choline | 23 | 17.39 | 21.74 | 60.87 | 2 (2) |
| Iodine | 26 | 7.69 | 7.69 | 84.62 | 2 (0.75) |
| Magnesium | 24 | 29.17 | 20.83 | 50.00 | 0.5 (3) |
| Calcium | 29 | 0.00 | 10.34 | 89.66 | 2 (2) |
| Iron | 30 | 0.00 | 0.00 | 100.00 | 2 (1) |
| Selenium | 24 | 16.67 | 20.83 | 62.50 | 1.5 (2) |
| DHA | 28 | 3.57 | 3.57 | 92.86 | 2 (1) |
| **For each of the following, please indicate how important it is to adjust the dietary supplementation recommendations for a person following a vegan diet who is hoping to get pregnant, who is currently pregnant, or who is lactating:** | | | | | |
|  | **Survey 1** | | | | |
|  | **n*** | **Slightly not to not at all important (%)** | **Neutral (%)** | **Slightly to extremely important (%)** | **Median (IQR)** |
| Vitamin B1 | 26 | 19.23 | 15.38 | 65.38 | 1.5 (3) |
| Vitamin B2 | 26 | 15.38 | 15.38 | 69.23 | 2 (3) |
| Vitamin B6 | 26 | 15.38 | 15.38 | 69.23 | 2 (3) |
| Vitamin B12 | 31 | 0.00 | 6.45 | 93.55 | 3 (0.5) |
| Vitamin D | 31 | 0.00 | 9.68 | 90.32 | 3 (1) |
| Vitamin K | 25 | 24.00 | 32.00 | 44.00 | 0 (2) |
| Folic acid | 30 | 16.67 | 10.00 | 73.33 | 2 (2.75) |
| Choline | 24 | 12.50 | 25.00 | 62.50 | 1.5 (3) |
| Iodine | 28 | 3.57 | 10.71 | 85.71 | 2 (1) |
| Magnesium | 25 | 20.00 | 24.00 | 56.00 | 1 (3) |
| Calcium | 31 | 0.00 | 6.45 | 93.55 | 3 (1) |
| Iron | 31 | 0.00 | 0.00 | 100.00 | 3 (0.5) |
| Selenium | 25 | 12.00 | 24.00 | 64.00 | 2 (3) |
| DHA | 28 | 7.14 | 0.00 | 92.86 | 3 (1) |
| **For each of the following, please indicate how important it is to adjust the dietary supplementation recommendations for a person with celiac disease who is hoping to get pregnant, who is currently pregnant, or who is lactating:** | | | | | |
|  | **Survey 1** | | | | |
|  | **n*** | **Slightly not to not at all important (%)** | **Neutral (%)** | **Slightly to extremely important (%)** | **Median (IQR)** |
| Vitamin B1 | 23 | 13.04 | 13.04 | 73.91 | 2 (2) |
| Vitamin B2 | 23 | 13.04 | 13.04 | 73.91 | 2 (2) |
| Vitamin B6 | 23 | 13.04 | 8.70 | 78.26 | 2 (1) |
| Vitamin B12 | 24 | 12.50 | 8.33 | 79.17 | 2 (1.25) |
| Vitamin D | 24 | 8.33 | 16.67 | 75.00 | 2 (1.5) |
| Vitamin K | 23 | 21.74 | 17.39 | 60.87 | 1 (2) |
| Folic acid | 24 | 4.17 | 4.17 | 91.67 | 2 (1) |
| Choline | 22 | 13.64 | 13.64 | 72.73 | 2 (1.75) |
| Iodine | 22 | 9.09 | 4.55 | 86.36 | 2 (0.75) |
| Magnesium | 21 | 14.29 | 14.29 | 71.43 | 2 (3) |
| Calcium | 24 | 12.50 | 4.17 | 83.33 | 2 (1) |
| Iron | 24 | 12.50 | 4.17 | 83.33 | 2 (1) |
| Selenium | 22 | 13.64 | 13.64 | 72.73 | 2 (2.5) |
| DHA | 22 | 13.64 | 4.55 | 81.82 | 2 (1) |
| **For each of the following, please indicate how important it is to adjust the dietary supplementation recommendations for a person with obesity who is hoping to get pregnant, who is currently pregnant, or who is lactating:** | | | | | |
|  | **Survey 1** | | | | |
|  | **n*** | **Slightly not to not at all important (%)** | **Neutral (%)** | **Slightly to extremely important (%)** | **Median (IQR)** |
| Vitamin B1 | 24 | 20.83 | 20.83 | 58.33 | 1 (1.25) |
| Vitamin B2 | 24 | 20.83 | 20.83 | 58.33 | 1 (1.25) |
| Vitamin B6 | 24 | 20.83 | 20.83 | 58.33 | 1 (2) |
| Vitamin B12 | 26 | 19.23 | 19.23 | 61.54 | 1 (2) |
| Vitamin D | 26 | 11.54 | 7.69 | 80.77 | 2 (1) |
| Vitamin K | 25 | 28.00 | 24.00 | 48.00 | 0 (3) |
| Folic acid | 28 | 7.14 | 3.57 | 89.29 | 3 (1) |
| Choline | 24 | 25.00 | 20.83 | 54.17 | 1 (2.25) |
| Iodine | 26 | 15.38 | 11.54 | 73.08 | 2 (1.75) |
| Magnesium | 24 | 12.50 | 20.83 | 66.67 | 1 (2) |
| Calcium | 25 | 12.00 | 20.00 | 68.00 | 2 (3) |
| Iron | 25 | 16.00 | 4.00 | 80.00 | 2 (2) |
| Selenium | 24 | 16.67 | 16.67 | 66.67 | 1 (2) |
| DHA | 25 | 16.00 | 4.00 | 80.00 | 2 (2) |
| **For each of the following, please indicate how important it is to adjust the dietary supplementation recommendations for a person with polycystic ovary syndrome (PCOS) who is hoping to get pregnant, who is currently pregnant, or who is lactating:** | | | | | |
|  | **Survey 1** | | | | |
|  | **n*** | **Slightly not to not at all important (%)** | **Neutral (%)** | **Slightly to extremely important (%)** | **Median (IQR)** |
| Vitamin B1 | 20 | 10.00 | 10.00 | 80.00 | 1 (1) |
| Vitamin B2 | 20 | 10.00 | 10.00 | 80.00 | 1 (1) |
| Vitamin B6 | 20 | 10.00 | 5.00 | 85.00 | 2 (1) |
| Vitamin B12 | 21 | 9.52 | 4.76 | 85.71 | 2 (1) |
| Vitamin D | 21 | 9.52 | 4.76 | 85.71 | 2 (1) |
| Vitamin K | 23 | 13.04 | 17.39 | 52.17 | 1 (2) |
| Folic acid | 23 | 0.00 | 0.00 | 100.00 | 2 (1) |
| Choline | 19 | 10.53 | 21.05 | 68.42 | 2 (2) |
| Iodine | 21 | 0.00 | 0.00 | 100.00 | 2 (1) |
| Magnesium | 20 | 5.00 | 15.00 | 80.00 | 1 (1.25) |
| Calcium | 20 | 5.00 | 5.00 | 90.00 | 2 (1.25) |
| Iron | 19 | 5.26 | 0.00 | 94.74 | 2 (0.5) |
| Selenium | 19 | 5.26 | 21.05 | 73.68 | 2 (1.5) |
| DHA | 20 | 5.00 | 0.00 | 95.00 | 2 (1) |
| **For each of the following, please indicate how important it is to adjust the dietary supplementation recommendations for a person with diabetes who is hoping to get pregnant, who is currently pregnant, or who is lactating:** | | | | | |
|  | **Survey 1** | | | | |
|  | **n*** | **Slightly not to not at all important (%)** | **Neutral (%)** | **Slightly to extremely important (%)** | **Median (IQR)** |
| Vitamin B1 | 23 | 21.74 | 13.04 | 65.22 | 1 (2) |
| Vitamin B2 | 23 | 21.74 | 13.04 | 65.22 | 1 (2) |
| Vitamin B6 | 24 | 20.83 | 12.50 | 62.50 | 1 (2) |
| Vitamin B12 | 25 | 20.00 | 8.00 | 72.00 | 2 (2) |
| Vitamin D | 26 | 11.54 | 3.85 | 84.62 | 2 (2) |
| Vitamin K | 23 | 26.08 | 26.08 | 47.83 | 0 (3) |
| Folic acid | 27 | 7.41 | 0.00 | 92.59 | 3 (1) |
| Choline | 23 | 21.74 | 21.74 | 56.52 | 1 (2) |
| Iodine | 24 | 12.50 | 8.33 | 79.17 | 2 (1) |
| Magnesium | 24 | 12.50 | 16.67 | 66.67 | 1 (2) |
| Calcium | 24 | 12.50 | 12.50 | 75.00 | 2 (1.25) |
| Iron | 23 | 17.39 | 0.00 | 82.61 | 2 (1) |
| Selenium | 23 | 17.39 | 13.04 | 69.57 | 1 (2) |
| DHA | 24 | 16.67 | 0.00 | 83.33 | 2 (2) |
| **For each of the following, please indicate how important it is to adjust the dietary supplementation recommendations for a person with a history of pregnancy and birth complications who is hoping to get pregnant, who is currently pregnant, or who is lactating:** | | | | | |
|  | **Survey 1** | | | | |
|  | **n*** | **Slightly not to not at all important (%)** | **Neutral (%)** | **Slightly to extremely important (%)** | **Median (IQR)** |
| Vitamin B1 | 25 | 12.00 | 16.00 | 72.00 | 2 (2) |
| Vitamin B2 | 25 | 12.00 | 16.00 | 72.00 | 2 (2) |
| Vitamin B6 | 25 | 12.00 | 12.00 | 76.00 | 2 (1) |
| Vitamin B12 | 25 | 12.00 | 12.00 | 76.00 | 2 (2) |
| Vitamin D | 26 | 11.54 | 11.54 | 76.92 | 2 (2) |
| Vitamin K | 24 | 16.67 | 25.00 | 58.33 | 1 (3) |
| Folic acid | 26 | 0.00 | 0.00 | 100.00 | 3 (1) |
| Choline | 24 | 12.50 | 20.83 | 66.67 | 2 (2) |
| Iodine | 25 | 4.00 | 4.00 | 92.00 | 2 (1) |
| Magnesium | 24 | 8.33 | 16.67 | 75.00 | 2 (2.25) |
| Calcium | 24 | 8.33 | 12.50 | 79.17 | 2 (2) |
| Iron | 25 | 4.00 | 0.00 | 96.00 | 2 (1) |
| Selenium | 25 | 12.00 | 20.00 | 68.00 | 1 (3) |
| DHA | 25 | 8.00 | 4.00 | 88.00 | 2 (1) |

*n refers to the number of valid responses; ‘NA/unsure’ responses were considered invalid and excluded.

DHA, docosahexaenoic acid; IQR, interquartile range; NA, not applicable; NTD, neutral tube defect.
