# Supplementary material for: International expert consensus on micronutrient supplement use during the early life course
Source: BMC Pregnancy Childbirth. 2025 Jan 20;25:44. doi: 10.1186/s12884-024-07123-5 (PMC11744953; doi:10.1186/s12884-024-07123-5)
Supplement: Supplementary file 3 — Supplementary Material 3: Additional File 3 [file 12884_2024_7123_MOESM3_ESM.docx]

**Supplementary Methods**

**International Expert Consensus on Micronutrient Supplement Use During the Early Life Course**

Irene Cetin, Roland Devlieger, Erika Isolauri, Rima Obeid, Francesca Parisi, Stefan Pilz, Lenie van Rossem, [Maternal Nutrition Delphi Study Group], Régine Steegers-Theunissen

**Contents**

[Supplementary Methods 4](#_Toc157612606)

[Literature Review 4](#_Toc157612607)

[Survey 1 Consensus Questions/Statements 5](#_Toc157612608)

[Survey 2 Consensus Questions/Statements 31](#_Toc157612638)

# **Supplementary Methods**

# *Literature Review*

To inform survey development and provide structure to steering committee discussions, a targeted literature review (TLR) was conducted, focusing on four key areas. Articles were screened, and duplicates, articles with irrelevant title/abstract/full text, or non-English articles were removed.

1. Guidelines
   1. Search date: 01 Nov 2022
   2. Search terms: ((guideline*[Title] OR recommendation*[Title]) AND (("pregnancy" OR "pregnant" OR "preconception" OR "periconception" OR "lactation" OR "breast feeding" OR breastfeeding"))) AND (("maternal nutrition" OR "micronutrient" OR "supplementation" OR "dietary supplements"[MeSH Terms] OR ("dietary"[All Fields] AND "supplements"[All Fields]) OR "dietary supplements"[All Fields])) AND (Europe OR Germany OR Poland OR Czech Republic OR Czechia OR Austria OR Slovakia OR Slovenia OR Hungary)
   3. Filters: Humans, within past 10 years
   4. Articles: 53 hits, 14 included
2. Micronutrients
   1. Search date: 31 Oct 2022
   2. Search terms: ("pregnancy" OR "pregnant" OR "preconception" OR "periconception" OR "lactation" OR "breast feeding" OR "breastfeeding") AND ("micronutrient" OR "supplementation" OR "iodine" OR "Docosahexaenoic acid" OR "choline" OR "folic acid" OR "folate" OR "Metafolin" OR "methylfolate" OR "L-methylfolate" OR "methyltetrahydrofolate" OR "Levomefolate" OR "vitamin") AND ("dose" OR "amount" OR “recommended daily” OR "intake") AND ("time" OR "duration" OR "weeks" or "months" or "days")
   3. Filters: Humans, within past 5 years, not reviews, and not guidelines
   4. Articles: 785 hits, 26 included
3. Risks and benefits
   1. Search date: 31 Oct 2022 Search terms: (((("pregnancy" OR "pregnant" OR "preconception" OR "lactation" OR "breast feeding" OR "breastfeeding")) AND ("micronutrient" OR "supplementation" OR "iodine" OR “Docosahexaenoic acid" OR "choline" OR "folic acid" OR "folate" OR "Metafolin" OR "methylfolate" OR "vitamin") AND ("fetal malformation" OR "birth defect" OR "fetal brain development" OR "infant brain development" OR "cognitive function" OR "neurocognitive function" OR "premature birth" OR "pre-term birth" OR (("pregnancy-induced hypertension") OR ("pregnancy" AND "hypertension") OR "gestational hypertension") OR "birth weight" OR "gestational diabetes" OR "gestational length" OR "miscarriage" OR "intra-uterine growth restriction" OR "IUGR") AND ("benefit" OR "risk")
   2. Filters: Humans, within past 5 years, not reviews, and not guidelines
   3. Articles: 725 hits, 40 included
4. 5-methyltetrahydrofolate
   1. Search date: 01 Nov 2022
   2. Search terms: MTHFR + folic acid: ("folic acid" OR "folate") AND (pregnancy OR "pregnant" OR "preconception") AND (("MTHFR gene polymorphism") OR ("MTHFR polymorphism")) AND (prevalence OR incidence) MTHFR + B6: (“vitamin B6" OR “B6") AND (pregnancy OR "pregnant" OR "preconception") AND (("MTHFR gene polymorphism") OR ("MTHFR polymorphism")) AND (prevalence OR incidence) MTHFR + B12: (“vitamin B12" OR “B12") AND (pregnancy OR "pregnant" OR "preconception") AND (("MTHFR gene polymorphism") OR ("MTHFR polymorphism")) AND (prevalence OR incidence)
5. Articles: 12 hits, 2 included

# **Survey 1 Consensus Questions/Statements**

General considerations around maternal nutrition

## Is there a need for increased awareness and education around maternal nutrition, including diet and dietary supplementation, for pregnant people and the general public?

### Please rate your level of agreement for each of the following stages of pregnancy:

Neither agree

Strongly

Somewhat

nor

Somewhat

Strongly Unsure/not

disagree Disagree

disagree

disagree

agree Agree

agree

applicable


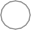

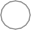

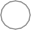

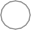

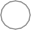

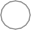

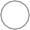

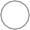


Pre-conception/planning phase


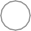

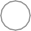

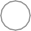

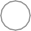

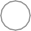

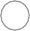

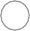

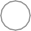

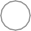

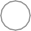

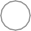

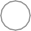

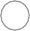

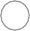
First trimester (Weeks 5–12)
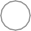


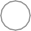

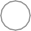

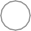

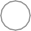

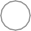

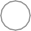

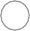

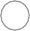


Second trimester (Weeks 13–27)

Third trimester (Weeks 28–41)
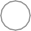


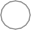

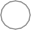

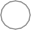

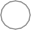

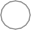

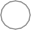

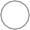

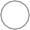


Postpartum/lactation

Additional comments


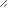


## Is there a need for increased awareness and education around maternal nutrition, including diet and dietary supplementation, for healthcare professionals?

### Please rate your level of agreement for each of the following stages of pregnancy:

Neither

Strongly

disagree Disagree

Somewhat disagree

agree nor disagree

Somewhat

agree Agree

Strongly agree

Unsure/not applicable


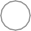

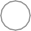

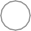

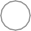

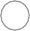

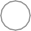

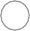

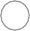


Pre- conception/planning phase


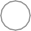

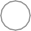

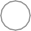

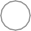

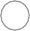

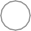

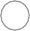

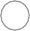
First trimester (Weeks 5–12)


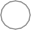

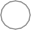

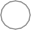

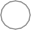

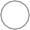

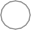

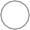

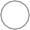


Second trimester (Weeks 13–27)

Third trimester (Weeks 28–41)


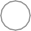

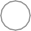

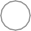

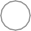

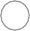

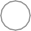

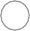

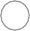


Postpartum/lactation


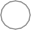

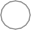

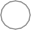

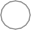

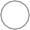

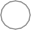

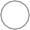

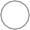
Additional comments


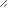


## What role should maternal nutritional care play in the delivery of healthcare?

### Please rate your level of agreement with each of the following statements:

Neither

Strongly

disagree Disagree

Somewhat disagree

agree nor disagree

Somewhat

agree Agree

Strongly agree

Unsure/not applicable


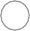

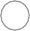

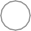

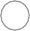

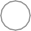

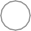

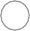

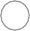


Maternal nutritional care should be considered a component of pre- conceptional and prenatal healthcare


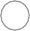

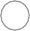

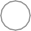

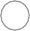

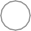

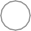

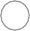

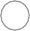
Maternal nutritional care should be reimbursed

Additional comments


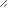


## How do you perceive the current guidelines for maternal nutrition, including diet and dietary supplementation?

### Please rate your level of agreement with each of the following statements:

Neither

Strongly

disagree Disagree

Somewhat disagree

agree nor disagree

Somewhat

agree Agree

Strongly agree

Unsure/not applicable


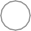


Current guidelines are clear and

consistent/harmonised across countries

Current guidelines are clear and

consistent/harmonised

across diﬀerent organisations

Current guidelines are reﬂective of the latest evidence

Current guidelines comprehensively cover all stages of pregnancy, from preconception through to lactation

Current guidelines are easy to access

Current guidelines are easy to follow

Additional comments

## Does gestational weight serve as a reliable proxy measure for maternal nutrition?

### Please rate your level of agreement with each of the following statements:

Neither

Strongly

disagree Disagree

Somewhat disagree

agree nor disagree

Somewhat

agree Agree

Strongly agree

Unsure/not applicable

Gestational weight is not a useful measure

Gestational weight alone provides a reliable proxy measure

Gestational weight should be combined with other metrics for a reliable proxy measure

Additional comments

## To ensure optimal nutrient intake from preconception through to lactation, which diet(s) should a person who is hoping to get pregnant, who is currently pregnant, or who is lactating be advised to follow?

### Please rate your level of agreement with each of the following statements:

Neither

Strongly

disagree Disagree

Somewhat disagree

agree nor disagree

Somewhat

agree Agree

Strongly agree

Unsure/not applicable

Speciﬁc diets should not be advised

Mediterranean diet

DASH diet (Dietary Approaches to Stop Hypertension)

Regular national dietary guidelines

Additional comments

## For each of the following stages of pregnancy, please indicate how important it is for healthcare professionals to provide recommendations on maternal diet:

Slightly

Not at all

Not

not

Slightly

Extremely Unsure/not

important

important

important Neutral

important Important

important

applicable

Pre- conception/planning phase

First trimester (Weeks 5–12)

Second trimester (Weeks 13–27)

Third trimester (Weeks 28–41)

Postpartum/lactation

Additional comments

## Please indicate how important you consider each of the following dietary supplementation approaches in achieving optimal nutrient intake from preconception through to lactation:

Not at all

Not

Slightly not

Slightly

Extremely Unsure/not

important

important

important Neutral

important Important

important

applicable

General multi- vitamin/multi- micronutrient supplementation (e.g. including folate, iron and other essential nutrients)

Individual vitamin/micronutrient supplementation

Approaches that are tailored to speciﬁc stages of pregnancy

Approaches that are tailored to a person’s individual

vitamin/micronutrient

status (e.g. based on serum vitamin D and ferritin assessment)

Additional comments

## For each of the following stages of pregnancy, please indicate how important it is for healthcare professionals to provide recommendations on maternal dietary supplementation:

Not at all

Not

Slightly not

Slightly

Extremely Unsure/not

important

important

important Neutral

important Important

important

applicable

Pre- conception/planning phase

First trimester (Weeks 5–12)

Second trimester (Weeks 13–27)

Third trimester (Weeks 28–41)

Postpartum/lactation

Additional comments

Nutrition from preconception through to lactation

## To ensure optimal nutrient intake, when should a person who is **hoping to get pregnant** be advised to start dietary supplementation?

### Please rate your level of agreement with each of the following statements:

Neither

Strongly

disagree Disagree

Somewhat disagree

agree nor disagree

Somewhat

agree Agree

Strongly agree

Unsure/not applicable

A balanced diet is suﬀicient; dietary supplementation should not be explicitly advised

Irrespective of when they hope to get pregnant, people of reproductive age

who are not using

contraception should be advised to start dietary supplementation

Up to 6 months before they hope to get pregnant

Up to 3 months

before they hope to get pregnant

Up to 1 month before they hope to get pregnant

Once it has been

conﬁrmed that they are pregnant

Additional comments

## For each of the following, please indicate how important dietary supplementation is for a person who is **hoping to get pregnant** and at low risk of pregnancy complications:

Slightly

Not at all

Not

not

Slightly

Extremely Unsure/not

important

important

important Neutral

important Important

important

applicable

Vitamin B1

Vitamin B2

Vitamin B6

Vitamin B12

Vitamin D

Vitamin K

Folic acid/folate

Choline

Iodine

Magnesium

Calcium

Iron

Selenium

Docosahexaenoic acid (DHA)

Additional comments

## For each of the following, please indicate how important dietary supplementation is during the **ﬁrst trimester** for a person who is at low risk of pregnancy complications:

Slightly

Not at all

Not

not

Slightly

Extremely Unsure/not

important

important

important Neutral

important Important

important

applicable

Vitamin B1

Vitamin B2

Vitamin B6

Vitamin B12

Vitamin D

Vitamin K

Folic acid/folate

Choline

Iodine

Magnesium

Calcium

Iron

Selenium

Docosahexaenoic acid (DHA)

Additional comments

## For each of the following, please indicate how important dietary supplementation is during the **second trimester** for a person who is at low risk of pregnancy complications:

Slightly

Not at all

Not

not

Slightly

Extremely Unsure/not

important

important

important Neutral

important Important

important

applicable

Vitamin B1

Vitamin B2

Vitamin B6

Vitamin B12

Vitamin D

Vitamin K

Folic acid/folate

Choline

Iodine

Magnesium

Calcium

Iron

Selenium

Docosahexaenoic acid (DHA)

Additional comments

## For each of the following, please indicate how important dietary supplementation is during the **third trimester** for a person who is at low risk of pregnancy complications:

Slightly

Not at all

Not

not

Slightly

Extremely Unsure/not

important

important

important Neutral

important Important

important

applicable

Vitamin B1

Vitamin B2

Vitamin B6

Vitamin B12

Vitamin D

Vitamin K

Folic acid/folate

Choline

Iodine

Magnesium

Calcium

Iron

Selenium

Docosahexaenoic acid (DHA)

Additional comments

## For each of the following, please indicate how important dietary supplementation is

**during lactation** for a person who is at low risk of pregnancy complications:

Slightly

Not at all

Not

not

Slightly

Extremely Unsure/not

important

important

important Neutral

important Important

important

applicable

Vitamin B1

Vitamin B2

Vitamin B6

Vitamin B12

Vitamin D

Vitamin K

Folic acid/folate

Choline

Iodine

Magnesium

Calcium

Iron

Selenium

Docosahexaenoic acid (DHA)

Additional comments

## To ensure optimal nutrient intake **postpartum**, should a person who is not breastfeeding be advised to continue dietary supplementation? If so, for how long?

### Please rate your level of agreement with each of the following statements:

Neither

Strongly

disagree Disagree

Somewhat disagree

agree nor disagree

Somewhat

agree Agree

Strongly agree

Unsure/not applicable

Continuing dietary supplementation should not be advised

Longer than 6 months after birth

Up to 6 months after birth

Up to 3 months after birth

Up to 1 month after birth

Additional comments

## To ensure optimal nutrient intake **following lactation**, should a person be advised to continue dietary supplementation after weaning? If so, for how long?

### Please rate your level of agreement with each of the following statements:

Neither

Strongly

disagree Disagree

Somewhat disagree

agree nor disagree

Somewhat

agree Agree

Strongly agree

Unsure/not applicable

Continuing dietary supplementation should not be advised

Up to 6 months after weaning

Up to 3 months after weaning

Up to 1 month after weaning

Additional comments

Considerations around speciﬁc micronutrients

## For each of the following, please indicate how concerned you are about the tolerable upper intake level* being exceeded?

### *The tolerable upper intake level refers to the maximum amount that can be consumed daily that is not expected to pose a health risk

Not at all

Not

Slightly

Slightly

Extremely Unsure/not

concerned concerned unconcerned Neutral

concerned Concerned concerned

applicable

Vitamin B6

Vitamin B12

Folic acid/folate

Iron

Iodine

Additional comments

## To ensure optimal nutrient intake from preconception through to lactation, please indicate your level of agreement with the following micronutrient-speciﬁc considerations:

Neither

Strongly

disagree Disagree

Somewhat disagree

agree nor disagree

Somewhat

agree Agree

Strongly agree

Unsure/not applicable

For folate supplementation, food fortiﬁcation is the optimal strategy

For folate supplementation, food fortiﬁcation is insuﬀicient to meet the needs of a person who is hoping to get pregnant, who is currently pregnant, or who is lactating

For folate supplementation, 5- MTHF should be used instead of folic acid

For iron supplementation, the type of salt that is used is important

For vitamin D supplementation, it is important to follow an individualised rather than “one-ﬁts-all” approach

For iodine supplementation, it is important to follow an individualised rather than “one-ﬁts-all” approach

Optimal levels of DHA should be achieved through supplementation rather than regular intake of ﬁsh

Additional comments

Risk and lifestyle-based approaches

## From preconception through to lactation, should dietary supplementation recommendations be personalised or tailored?

### Please rate your level of agreement with the following statements:

Neither

Strongly

disagree Disagree

Somewhat disagree

agree nor disagree

Somewhat

agree Agree

Strongly agree

Unsure/not applicable

The recommendations are universally applicable and do not need to be tailored or personalised

The recommendations should be personalised

The recommendations should be tailored to a person’s lifestyle

The recommendations

should be tailored to

a person’s medical conditions

The recommendations should be tailored to a person’s history of pregnancy and birth complications

Additional comments

## From preconception through to lactation, please indicate how important is it to have tailored approaches to diet and/or dietary supplementation for the following groups:

Not at all

Not

Slightly not

Slightly

Extremely Unsure/not

important

important

important Neutral

important Important

important

applicable

People who are athletes or highly active

People who are

following a vegan diet

People who are following a vegetarian diet

People with coeliac disease

People with obesity

People with

polycystic ovary syndrome (PCOS)

People with diabetes

People with a history of pregnancy and birth complications (e.g. history of previous NTDs, or

those at risk for

congenital malformations, pre- term births, pre- eclampsia, gestational diabetes)

Additional comments

## For each of the following, please indicate how important it is to adjust the dietary supplementation recommendations for an **athlete or highly active person** who is hoping to get pregnant, who is currently pregnant, or who is lactating:

Slightly

Not at all

Not

not

Slightly

Extremely Unsure/not

important

important

important Neutral

important Important

important

applicable

Vitamin B1

Vitamin B2

Vitamin B6

Vitamin B12

Vitamin D

Vitamin K

Folic acid/folate

Choline

Iodine

Magnesium

Calcium

Iron

Selenium

Docosahexaenoic acid (DHA)

Additional comments

## For each of the following, please indicate how important it is to adjust the dietary supplementation recommendations for a person following a **vegetarian diet** who is hoping to get pregnant, who is currently pregnant, or who is lactating:

Slightly

Not at all

Not

not

Slightly

Extremely Unsure/not

important

important

important Neutral

important Important

important

applicable

Vitamin B1

Vitamin B2

Vitamin B6

Vitamin B12

Vitamin D

Vitamin K

Folic acid/folate

Choline

Iodine

Magnesium

Calcium

Iron

Selenium

Docosahexaenoic acid (DHA)

Additional comments

## For each of the following, please indicate how important it is to adjust the dietary supplementation recommendations for a person following a **vegan diet** who is hoping to get pregnant, who is currently pregnant, or who is lactating:

Slightly

Not at all

Not

not

Slightly

Extremely Unsure/not

important

important

important Neutral

important Important

important

applicable

Vitamin B1

Vitamin B2

Vitamin B6

Vitamin B12

Vitamin D

Vitamin K

Folic acid/folate

Choline

Iodine

Magnesium

Calcium

Iron

Selenium

Docosahexaenoic acid (DHA)

Additional comments

## For each of the following, please indicate how important it is to adjust the dietary supplementation recommendations for a person with **coeliac disease** who is hoping to get pregnant, who is currently pregnant, or who is lactating:

Slightly

Not at all

Not

not

Slightly

Extremely Unsure/not

important

important

important Neutral

important Important

important

applicable

Vitamin B1

Vitamin B2

Vitamin B6

Vitamin B12

Vitamin D

Vitamin K

Folic acid/folate

Choline

Iodine

Magnesium

Calcium

Iron

Selenium

Docosahexaenoic acid (DHA)

Additional comments

## For each of the following, please indicate how important it is to adjust the dietary supplementation recommendations for a person with **obesity** who is hoping to get pregnant, who is currently pregnant, or who is lactating:

Slightly

Not at all

Not

not

Slightly

Extremely Unsure/not

important

important

important Neutral

important Important

important

applicable

Vitamin B1

Vitamin B2

Vitamin B6

Vitamin B12

Vitamin D

Vitamin K

Folic acid/folate

Choline

Iodine

Magnesium

Calcium

Iron

Selenium

Docosahexaenoic acid (DHA)

Additional comments

## For each of the following, please indicate how important it is to adjust the dietary supplementation recommendations for a person with **polycystic ovary syndrome (PCOS)** who is hoping to get pregnant, who is currently pregnant, or who is lactating:

Slightly

Not at all

Not

not

Slightly

Extremely Unsure/not

important

important

important Neutral

important Important

important

applicable

Vitamin B1

Vitamin B2

Vitamin B6

Vitamin B12

Vitamin D

Vitamin K

Folic acid/folate

Choline

Iodine

Magnesium

Calcium

Iron

Selenium

Docosahexaenoic acid (DHA)

Additional comments

## For each of the following, please indicate how important it is to adjust the dietary supplementation recommendations for a person with **diabetes** who is hoping to get pregnant, who is currently pregnant, or who is lactating:

Slightly

Not at all

Not

not

Slightly

Extremely Unsure/not

important

important

important Neutral

important Important

important

applicable

Vitamin B1

Vitamin B2

Vitamin B6

Vitamin B12

Vitamin D

Vitamin K

Folic acid/folate

Choline

Iodine

Magnesium

Calcium

Iron

Selenium

Docosahexaenoic acid (DHA)

Additional comments

## For each of the following, please indicate how important it is to adjust the dietary supplementation recommendations for a person with a **history of pregnancy and birth complications** who is hoping to get pregnant, who is currently pregnant, or who is lactating:

Not at all

Not

Slightly not

Slightly

Extremely Unsure/not

important

important

important Neutral

important Important

important

applicable

Vitamin B1

Vitamin B2

Vitamin B6

Vitamin B12

Vitamin D

Vitamin K

Folic acid/folate

Choline

Iodine

Magnesium

Calcium

Iron

Selenium

Docosahexaenoic acid (DHA)

Additional comments

# **Survey 2 Consensus Questions/Statements**

Section 1 of 4: Preconception

For each of the following, please indicate how important dietary supplementation is for a person who is **hoping to get pregnant** and at **low risk** of pregnancy complications

Slightly

Not at all

Not

not

Slightly

Extremely Unsure/not

important

important

important Neutral

important Important

important

applicable

**Vitamin D** *(almost consensus on importance in Survey 1: 74.19% selected slightly to extremely important)*

**Iodine**

*(almost consensus in Survey 1: 70.37% selected slightly to extremely important)*

**Iron**

*(almost consensus in Survey 1: 70.00% selected slightly to extremely important)*

Additional comments

Preconception: Vitamin B1 *(no consensus on importance in Survey 1)*

Please rate your perception of the strength/quality of evidence that supplementation of **vitamin B1** during **preconception** for a person who is at **low risk** of complications improves pregnancy outcomes and/or foetal development

Very weak Weak

Somewhat

weak Neutral

Somewhat

strong Strong Very strong

Unsure/not applicable

Additional comments

*[If somewhat to very weak or neutral]*

Please provide a reasoning for your answer

There is a lack of evidence

There are discrepancies in existing evidence Existing evidence is of low quality

Other (please explain)

Preconception: Vitamin B2 *(no consensus on importance in Survey 1)*

Please rate your perception of the strength/quality of evidence that supplementation of **vitamin B2** during **preconception** for a person who is at **low risk** of complications improves pregnancy outcomes and/or foetal development

Very weak Weak

Somewhat

weak Neutral

Somewhat

strong Strong Very strong

Unsure/not applicable

Additional comments

*[If somewhat to very weak]*

Please provide a reasoning for your answer

There is a lack of evidence

There are discrepancies in existing evidence Existing evidence is of low quality

Other (please explain)

Preconception: Vitamin B6 *(no consensus on importance in Survey 1)*

Please rate your perception of the strength/quality of evidence that supplementation of **vitamin B6** during **preconception** for a person who is at **low risk** of complications improves pregnancy outcomes and/or foetal development

Very weak Weak

Somewhat

weak Neutral

Somewhat

strong Strong Very strong

Unsure/not applicable

Additional comments

*[If somewhat to very weak or neutral]*

Please provide a reasoning for your answer

There is a lack of evidence

There are discrepancies in existing evidence Existing evidence is of low quality

Other (please explain)

Preconception: Vitamin B12 *(no consensus on importance in Survey 1)*

Please rate your perception of the strength/quality of evidence that supplementation of **vitamin B12** during **preconception** for a person who is at **low risk** of complications improves pregnancy outcomes and/or foetal development

Very weak Weak

Somewhat

weak Neutral

Somewhat

strong Strong Very strong

Unsure/not applicable

Additional comments

*[If somewhat to very weak or neutral]*

Please provide a reasoning for your answer

There is a lack of evidence

There are discrepancies in existing evidence Existing evidence is of low quality

Other (please explain)

Preconception: Vitamin D *(almost consensus on importance in Survey 1)*

Please rate your perception of the strength/quality of evidence that supplementation of **vitamin D** during **preconception** for a person who is at **low risk** of complications improves pregnancy outcomes and/or foetal development

Very weak Weak

Somewhat

weak Neutral

Somewhat

strong Strong Very strong

Unsure/not applicable

Additional comments

*[If somewhat to very weak or neutral]*

Please provide a reasoning for your answer

There is a lack of evidence

There are discrepancies in existing evidence Existing evidence is of low quality

Other (please explain)

Preconception: Vitamin K *(no consensus on importance in Survey 1)*

Please rate your perception of the strength/quality of evidence that supplementation of **vitamin K** during **preconception** for a person who is at **low risk** of complications improves pregnancy outcomes and/or foetal development

Very weak Weak

Somewhat

weak Neutral

Somewhat

strong Strong Very strong

Unsure/not applicable

Additional comments

*[If somewhat to very weak or neutral]*

Please provide a reasoning for your answer

There is a lack of evidence

There are discrepancies in existing evidence Existing evidence is of low quality

Other (please explain)

Preconception: Folic acid/folate *(consensus on importance in Survey 1)*

Please rate your perception of the strength/quality of evidence that supplementation of **folic acid/folate** during **preconception** for a person who is at **low risk** of complications improves pregnancy outcomes and/or foetal development

Very weak Weak

Somewhat

weak Neutral

Somewhat

strong Strong Very strong

Unsure/not applicable

Additional comments

*[If somewhat to very weak or neutral]*

Please provide a reasoning for your answer

There is a lack of evidence

There are discrepancies in existing evidence Existing evidence is of low quality

Other (please explain)

Preconception: Choline *(no consensus on importance in Survey 1)*

Please rate your perception of the strength/quality of evidence that supplementation of **choline** during **preconception** for a person who is at **low risk** of complications improves pregnancy outcomes and/or foetal development

Very weak Weak

Somewhat

weak Neutral

Somewhat

strong Strong Very strong

Unsure/not applicable

Additional comments

*[If somewhat to very weak or neutral]*

Please provide a reasoning for your answer

There is a lack of evidence

There are discrepancies in existing evidence Existing evidence is of low quality

Other (please explain)

Preconception: Iodine *(almost consensus on importance in Survey 1)*

Please rate your perception of the strength/quality of evidence that supplementation of **iodine** during **preconception** for a person who is at **low risk** of complications improves pregnancy outcomes and/or foetal development

Very weak Weak

Somewhat

weak Neutral

Somewhat

strong Strong Very strong

Unsure/not applicable

Additional comments

*[If somewhat to very weak or neutral]*

Please provide a reasoning for your answer

There is a lack of evidence

There are discrepancies in existing evidence Existing evidence is of low quality

Other (please explain)

Preconception: Magnesium *(no consensus on importance in Survey 1)*

Please rate your perception of the strength/quality of evidence that supplementation of **magnesium** during **preconception** for a person who is at **low risk** of complications improves pregnancy outcomes and/or foetal development

Very weak Weak

Somewhat

weak Neutral

Somewhat

strong Strong Very strong

Unsure/not applicable

Additional comments

*[If somewhat to very weak or neutral]*

Please provide a reasoning for your answer

There is a lack of evidence

There are discrepancies in existing evidence Existing evidence is of low quality

Other (please explain)

Preconception: Calcium *(no consensus on importance in Survey 1)*

Please rate your perception of the strength/quality of evidence that supplementation of **calcium** during **preconception** for a person who is at **low risk** of complications improves pregnancy outcomes and/or foetal development

Very weak Weak

Somewhat

weak Neutral

Somewhat

strong Strong Very strong

Unsure/not applicable

Additional comments

*[If somewhat to very weak or neutral]*

Please provide a reasoning for your answer

There is a lack of evidence

There are discrepancies in existing evidence Existing evidence is of low quality

Other (please explain)

Preconception: Iron *(almost consensus on importance in Survey 1)*

Please rate your perception of the strength/quality of evidence that supplementation of **iron** during **preconception** for a person who is at **low risk** of complications improves pregnancy outcomes and/or foetal development

Very weak Weak

Somewhat

weak Neutral

Somewhat

strong Strong Very strong

Unsure/not applicable

Additional comments

*[If somewhat to very weak or neutral]*

Please provide a reasoning for your answer

There is a lack of evidence

There are discrepancies in existing evidence Existing evidence is of low quality

Other (please explain)

Preconception: Selenium *(no consensus on importance in Survey 1)*

Please rate your perception of the strength/quality of evidence that supplementation of **selenium** during **preconception** for a person who is at **low risk** of complications improves pregnancy outcomes and/or foetal development

Very weak Weak

Somewhat

weak Neutral

Somewhat

strong Strong Very strong

Unsure/not applicable

Additional comments

*[If somewhat to very weak or neutral]*

Please provide a reasoning for your answer

There is a lack of evidence

There are discrepancies in existing evidence Existing evidence is of low quality

Other (please explain)

Preconception: Docosahexaenoic acid *(DHA; no consensus on importance in Survey*

*1)*

Please rate your perception of the strength/quality of evidence that supplementation of **DHA** during **preconception** for a person who is at **low risk** of complications improves pregnancy outcomes and/or foetal development

Very weak Weak

Somewhat

weak Neutral

Somewhat

strong Strong Very strong

Unsure/not applicable

Additional comments

*[If somewhat to very weak or neutral]*

Please provide a reasoning for your answer

There is a lack of evidence

There are discrepancies in existing evidence Existing evidence is of low quality

Other (please explain)

Section 2 of 4: First trimester

Slightly

Not at all

Not

not

Slightly

Extremely Unsure/not

important

important

important Neutral

important Important

important

applicable

**Vitamin B12** *(almost consensus in Survey 1: 70.37% selected slightly to extremely important in Survey 1)*

**Iron**

*(almost consensus in Survey 1: 74.19% selected slightly to extremely important in Survey 1)*

Additional comments

First trimester: Vitamin B1 *(no consensus on importance in Survey 1)*

Please rate your perception of the strength/quality of evidence that supplementation of **vitamin B1** during the **ﬁrst trimester** for a person who is at **low risk** of complications improves pregnancy outcomes and/or foetal development

Very weak Weak

Somewhat

weak Neutral

Somewhat

strong Strong Very strong

Unsure/not applicable

Additional comments

*[If somewhat to very weak or neutral]*

Please provide a reasoning for your answer

There is a lack of evidence

There are discrepancies in existing evidence Existing evidence is of low quality

Other (please explain)

First trimester: Vitamin B2 *(no consensus on importance in Survey 1)*

Please rate your perception of the strength/quality of evidence that supplementation of **vitamin B2** during the **ﬁrst trimester** for a person who is at **low risk** of complications improves pregnancy outcomes and/or foetal development

Very weak Weak

Somewhat

weak Neutral

Somewhat

strong Strong Very strong

Unsure/not applicable

Additional comments

*[If somewhat to very weak or neutral]*

Please provide a reasoning for your answer

There is a lack of evidence

There are discrepancies in existing evidence Existing evidence is of low quality

Other (please explain)

First trimester: Vitamin B6 *(no consensus on importance in Survey 1)*

Please rate your perception of the strength/quality of evidence that supplementation of **vitamin B6** during the **ﬁrst trimester** for a person who is at **low risk** of complications improves pregnancy outcomes and/or foetal development

Very weak Weak

Somewhat

weak Neutral

Somewhat

strong Strong Very strong

Unsure/not applicable

Additional comments

*[If somewhat to very weak or neutral]*

Please provide a reasoning for your answer

There is a lack of evidence

There are discrepancies in existing evidence Existing evidence is of low quality

Other (please explain)

First trimester: Vitamin B12 *(almost consensus on importance in Survey 1)*

Please rate your perception of the strength/quality of evidence that supplementation of **vitamin B12** during the **ﬁrst trimester** for a person who is at **low risk** of complications improves pregnancy outcomes and/or foetal development

Very weak Weak

Somewhat

weak Neutral

Somewhat

strong Strong Very strong

Unsure/not applicable

Additional comments

*[If somewhat to very weak or neutral]*

Please provide a reasoning for your answer

There is a lack of evidence

There are discrepancies in existing evidence Existing evidence is of low quality

Other (please explain)

First trimester: Vitamin D *(consensus on importance in Survey 1)*

Please rate your perception of the strength/quality of evidence that supplementation of **vitamin D** during the **ﬁrst trimester** for a person who is at **low risk** of complications improves pregnancy outcomes and/or foetal development

Very weak Weak

Somewhat

weak Neutral

Somewhat

strong Strong Very strong

Unsure/not applicable

Additional comments

*[If somewhat to very weak or neutral]*

Please provide a reasoning for your answer

There is a lack of evidence

There are discrepancies in existing evidence Existing evidence is of low quality

Other (please explain)

First trimester: Vitamin K *(no consensus on importance in Survey 1)*

Please rate your perception of the strength/quality of evidence that supplementation of **vitamin K** during the **ﬁrst trimester** for a person who is at **low risk** of complications improves pregnancy outcomes and/or foetal development

Very weak Weak

Somewhat

weak Neutral

Somewhat

strong Strong Very strong

Unsure/not applicable

Additional comments

*[If somewhat to very weak or neutral]*

Please provide a reasoning for your answer

There is a lack of evidence

There are discrepancies in existing evidence Existing evidence is of low quality

Other (please explain)

First trimester: Folic acid/folate *(consensus on importance in Survey 1)*

Please rate your perception of the strength/quality of evidence that supplementation of **folic acid/folate** during the **ﬁrst trimester** for a person who is at **low risk** of complications improves pregnancy outcomes and/or foetal development

Very weak Weak

Somewhat

weak Neutral

Somewhat

strong Strong Very strong

Unsure/not applicable

Additional comments

*[If somewhat to very weak or neutral]*

Please provide a reasoning for your answer

There is a lack of evidence

There are discrepancies in existing evidence Existing evidence is of low quality

Other (please explain)

First trimester: Choline *(no consensus on importance in Survey 1)*

Please rate your perception of the strength/quality of evidence that supplementation of **choline** during the **ﬁrst trimester** for a person who is at **low risk** of complications improves pregnancy outcomes and/or foetal development

Very weak Weak

Somewhat

weak Neutral

Somewhat

strong Strong Very strong

Unsure/not applicable

Additional comments

*[If somewhat to very weak or neutral]*

Please provide a reasoning for your answer

There is a lack of evidence

There are discrepancies in existing evidence Existing evidence is of low quality

Other (please explain)

First trimester: Iodine *(consensus on importance in Survey 1)*

Please rate your perception of the strength/quality of evidence that supplementation of **iodine** during the **ﬁrst trimester** for a person who is at **low risk** of complications improves pregnancy outcomes and/or foetal development

Very weak Weak

Somewhat

weak Neutral

Somewhat

strong Strong Very strong

Unsure/not applicable

Additional comments

*[If somewhat to very weak or neutral]*

Please provide a reasoning for your answer

There is a lack of evidence

There are discrepancies in existing evidence Existing evidence is of low quality

Other (please explain)

First trimester: Magnesium *(no consensus on importance in Survey 1)*

Please rate your perception of the strength/quality of evidence that supplementation of **magnesium** during the **ﬁrst trimester** for a person who is at **low risk** of complications improves pregnancy outcomes and/or foetal development

Very weak Weak

Somewhat

weak Neutral

Somewhat

strong Strong Very strong

Unsure/not applicable

Additional comments

*[If somewhat to very weak or neutral]*

Please provide a reasoning for your answer

There is a lack of evidence

There are discrepancies in existing evidence Existing evidence is of low quality

Other (please explain)

First trimester: Calcium *(no consensus on importance in Survey 1)*

Please rate your perception of the strength/quality of evidence that supplementation of **calcium** during the **ﬁrst trimester** for a person who is at **low risk** of complications improves pregnancy outcomes and/or foetal development

Very weak Weak

Somewhat

weak Neutral

Somewhat

strong Strong Very strong

Unsure/not applicable

Additional comments

*[If somewhat to very weak or neutral]*

Please provide a reasoning for your answer

There is a lack of evidence

There are discrepancies in existing evidence Existing evidence is of low quality

Other (please explain)

First trimester: Iron *(almost consensus on importance in Survey 1)*

Please rate your perception of the strength/quality of evidence that supplementation of **iron** during the **ﬁrst trimester** for a person who is at **low risk** of complications improves pregnancy outcomes and/or foetal development

Very weak Weak

Somewhat

weak Neutral

Somewhat

strong Strong Very strong

Unsure/not applicable

Additional comments

*[If somewhat to very weak or neutral]*

Please provide a reasoning for your answer

There is a lack of evidence

There are discrepancies in existing evidence Existing evidence is of low quality

Other (please explain)

First trimester: Selenium *(no consensus on importance in Survey 1)*

Please rate your perception of the strength/quality of evidence that supplementation of **selenium** during the **ﬁrst trimester** for a person who is at **low risk** of complications improves pregnancy outcomes and/or foetal development

Very weak Weak

Somewhat

weak Neutral

Somewhat

strong Strong Very strong

Unsure/not applicable

Additional comments

*[If somewhat to very weak or neutral]*

Please provide a reasoning for your answer

There is a lack of evidence

There are discrepancies in existing evidence Existing evidence is of low quality

Other (please explain)

First trimester: Docosahexaenoic acid *(DHA; consensus on importance in Survey 1)*

Please rate your perception of the strength/quality of evidence that supplementation of **DHA** during the **ﬁrst trimester** for a person who is at **low risk** of complications improves pregnancy outcomes and/or foetal development

Very weak Weak

Somewhat

weak Neutral

Somewhat

strong Strong Very strong

Unsure/not applicable

Additional comments

*[If somewhat to very weak or neutral]*

Please provide a reasoning for your answer

There is a lack of evidence

There are discrepancies in existing evidence Existing evidence is of low quality

Other (please explain)

Section 3 of 5: Second trimester

For each of the following, please indicate how important dietary supplementation is during the **second trimester** for a person who is at **low risk** of pregnancy complications

Slightly

Not at all

Not

not

Slightly

Extremely Unsure/not

important Important important important Neutral important Important important applicable

**Vitamin B12** *(almost consensus in Survey 1:*

*70.37%*

*selected slightly to extremely important in Survey 1)*

**Folic acid/folate** *(almost consensus in Survey 1:*

*71.88%*

*selected slightly to extremely important in Survey 1)*

Additional comments

Second trimester: Vitamin B1 *(no consensus on importance in Survey 1)*

Please rate your perception of the strength/quality of evidence that supplementation of **vitamin B1** during the **second trimester** for a person who is at **low risk** of complications improves pregnancy outcomes and/or foetal development

Very weak Weak

Somewhat

weak Neutral

Somewhat

strong Strong Very strong

Unsure/not applicable

Additional comments

*[If somewhat to very weak or neutral]*

Please provide a reasoning for your answer

There is a lack of evidence

There are discrepancies in existing evidence Existing evidence is of low quality

Other (please explain)

Second trimester: Vitamin B2 *(no consensus on importance in Survey 1)*

Please rate your perception of the strength/quality of evidence that supplementation of **vitamin B2** during the **second trimester** for a person who is at **low risk** of complications improves pregnancy outcomes and/or foetal development

Very weak Weak

Somewhat

weak Neutral

Somewhat

strong Strong Very strong

Unsure/not applicable

Additional comments

*[If somewhat to very weak or neutral]*

Please provide a reasoning for your answer

There is a lack of evidence

There are discrepancies in existing evidence Existing evidence is of low quality

Other (please explain)

Second trimester: Vitamin B6 *(no consensus on importance in Survey 1)*

Please rate your perception of the strength/quality of evidence that supplementation of **vitamin B6** during the **second trimester** for a person who is at **low risk** of complications improves pregnancy outcomes and/or foetal development

Very weak Weak

Somewhat

weak Neutral

Somewhat

strong Strong Very strong

Unsure/not applicable

Additional comments

*[If somewhat to very weak or neutral]*

Please provide a reasoning for your answer

There is a lack of evidence

There are discrepancies in existing evidence Existing evidence is of low quality

Other (please explain)

Second trimester: Vitamin B12 *(almost consensus on importance in Survey 1)*

Please rate your perception of the strength/quality of evidence that supplementation of **vitamin B12** during the **second trimester** for a person who is at **low risk** of complications improves pregnancy outcomes and/or foetal development

Very weak Weak

Somewhat

weak Neutral

Somewhat

strong Strong Very strong

Unsure/not applicable

Additional comments

*[If somewhat to very weak or neutral]*

Please provide a reasoning for your answer

There is a lack of evidence

There are discrepancies in existing evidence Existing evidence is of low quality

Other (please explain)

Second trimester: Vitamin D *(consensus on importance in Survey 1)*

Please rate your perception of the strength/quality of evidence that supplementation of **vitamin D** during the **second trimester** for a person who is at **low risk** of complications improves pregnancy outcomes and/or foetal development

Very weak Weak

Somewhat

weak Neutral

Somewhat

strong Strong Very strong

Unsure/not applicable

Additional comments

*[If somewhat to very weak or neutral]*

Please provide a reasoning for your answer

There is a lack of evidence

There are discrepancies in existing evidence Existing evidence is of low quality

Other (please explain)

Second trimester: Vitamin K *(no consensus on importance in Survey 1)*

Please rate your perception of the strength/quality of evidence that supplementation of **vitamin K** during the **second trimester** for a person who is at **low risk** of complications improves pregnancy outcomes and/or foetal development

Very weak Weak

Somewhat

weak Neutral

Somewhat

strong Strong Very strong

Unsure/not applicable

Additional comments

*[If somewhat to very weak or neutral]*

Please provide a reasoning for your answer

There is a lack of evidence

There are discrepancies in existing evidence Existing evidence is of low quality

Other (please explain)

Second trimester: Folic acid/folate *(almost consensus on importance in Survey 1)*

Please rate your perception of the strength/quality of evidence that supplementation of **folic acid/folate** during the **second trimester** for a person who is at **low risk** of complications improves pregnancy outcomes and/or foetal development

Very weak Weak

Somewhat

weak Neutral

Somewhat

strong Strong Very strong

Unsure/not applicable

Additional comments

*[If somewhat to very weak or neutral]*

Please provide a reasoning for your answer

There is a lack of evidence

There are discrepancies in existing evidence Existing evidence is of low quality

Other (please explain)

Second trimester: Choline *(no consensus on importance in Survey 1)*

Please rate your perception of the strength/quality of evidence that supplementation of **choline** during the **second trimester** for a person who is at **low risk** of complications improves pregnancy outcomes and/or foetal development

Very weak Weak

Somewhat

weak Neutral

Somewhat

strong Strong Very strong

Unsure/not applicable

Additional comments

*[If somewhat to very weak or neutral]*

Please provide a reasoning for your answer

There is a lack of evidence

There are discrepancies in existing evidence Existing evidence is of low quality

Other (please explain)

Second trimester: Iodine *(consensus on importance in Survey 1)*

Please rate your perception of the strength/quality of evidence that supplementation of **iodine** during the **second trimester** for a person who is at **low risk** of complications improves pregnancy outcomes and/or foetal development

Very weak Weak

Somewhat

weak Neutral

Somewhat

strong Strong Very strong

Unsure/not applicable

Additional comments

*[If somewhat to very weak or neutral]*

Please provide a reasoning for your answer

There is a lack of evidence

There are discrepancies in existing evidence Existing evidence is of low quality

Other (please explain)

Second trimester: Magnesium *(no consensus on importance in Survey 1)*

Please rate your perception of the strength/quality of evidence that supplementation of **magnesium** during the **second trimester** for a person who is at **low risk** of complications improves pregnancy outcomes and/or foetal development

Very weak Weak

Somewhat

weak Neutral

Somewhat

strong Strong Very strong

Unsure/not applicable

Additional comments

*[If somewhat to very weak or neutral]*

Please provide a reasoning for your answer

There is a lack of evidence

There are discrepancies in existing evidence Existing evidence is of low quality

Other (please explain)

Second trimester: Calcium *(no consensus on importance in Survey 1)*

Please rate your perception of the strength/quality of evidence that supplementation of **calcium** during the **second trimester** for a person who is at **low risk** of complications improves pregnancy outcomes and/or foetal development

Very weak Weak

Somewhat

weak Neutral

Somewhat

strong Strong Very strong

Unsure/not applicable

Additional comments

*[If somewhat to very weak or neutral]*

Please provide a reasoning for your answer

There is a lack of evidence

There are discrepancies in existing evidence Existing evidence is of low quality

Other (please explain)

Second trimester: Iron *(consensus on importance in Survey 1)*

Please rate your perception of the strength/quality of evidence that supplementation of **iron** during the **second trimester** for a person who is at **low risk** of complications improves pregnancy outcomes and/or foetal development

Very weak Weak

Somewhat

weak Neutral

Somewhat

strong Strong Very strong

Unsure/not applicable

Additional comments

*[If somewhat to very weak or neutral]*

Please provide a reasoning for your answer

There is a lack of evidence

There are discrepancies in existing evidence Existing evidence is of low quality

Other (please explain)

Second trimester: Selenium *(no consensus on importance in Survey 1)*

Please rate your perception of the strength/quality of evidence that supplementation of **selenium** during the **second trimester** for a person who is at **low risk** of complications improves pregnancy outcomes and/or foetal development

Very weak Weak

Somewhat

weak Neutral

Somewhat

strong Strong Very strong

Unsure/not applicable

Additional comments

*[If somewhat to very weak or neutral]*

Please provide a reasoning for your answer

There is a lack of evidence

There are discrepancies in existing evidence Existing evidence is of low quality

Other (please explain)

Second trimester: Docosahexaenoic acid *(DHA; consensus on importance in Survey*

*1)*

Please rate your perception of the strength/quality of evidence that supplementation of **DHA** during the **second trimester** for a person who is at **low risk** of complications improves pregnancy outcomes and/or foetal development

Very weak Weak

Somewhat

weak Neutral

Somewhat

strong Strong Very strong

Unsure/not applicable

Additional comments

*[If somewhat to very weak or neutral]*

Please provide a reasoning for your answer

There is a lack of evidence

There are discrepancies in existing evidence Existing evidence is of low quality

Other (please explain)

Section 4 of 5: Third trimester

For each of the following, please indicate how important dietary supplementation is during the **third trimester** for a person who is at **low risk** of pregnancy complications

Slightly

Not at all

Not

not

Slightly

Extremely Unsure/not

important Important important important Neutral important Important important applicable

**Vitamin B12** *(almost consensus in Survey 1:*

*70.37%*

*selected slightly to extremely important in Survey 1)*

**Iodine** *(almost consensus in Survey 1:*

*71.43%*

*selected slightly to extremely important in Survey 1)*

**Calcium** *(almost consensus in Survey 1:*

*72.41%*

*selected slightly to extremely important in Survey 1)*

Additional comments

Third trimester: Vitamin B1 *(no consensus on importance in Survey 1)*

Please rate your perception of the strength/quality of evidence that supplementation of **vitamin B1** during the **third trimester** for a person who is at **low risk** of complications improves pregnancy outcomes and/or foetal development

Very weak Weak

Somewhat

weak Neutral

Somewhat

strong Strong Very strong

Unsure/not applicable

Additional comments

*[If somewhat to very weak or neutral]*

Please provide a reasoning for your answer

There is a lack of evidence

There are discrepancies in existing evidence Existing evidence is of low quality

Other (please explain)

Third trimester: Vitamin B2 *(no consensus on importance in Survey 1)*

Please rate your perception of the strength/quality of evidence that supplementation of **vitamin B2** during the **third trimester** for a person who is at **low risk** of complications improves pregnancy outcomes and/or foetal development

Very weak Weak

Somewhat

weak Neutral

Somewhat

strong Strong Very strong

Unsure/not applicable

Additional comments

*[If somewhat to very weak or neutral]*

Please provide a reasoning for your answer

There is a lack of evidence

There are discrepancies in existing evidence Existing evidence is of low quality

Other (please explain)

Third trimester: Vitamin B6 *(no consensus on importance in Survey 1)*

Please rate your perception of the strength/quality of evidence that supplementation of **vitamin B6** during the **third trimester** for a person who is at **low risk** of complications improves pregnancy outcomes and/or foetal development

Very weak Weak

Somewhat

weak Neutral

Somewhat

strong Strong Very strong

Unsure/not applicable

Additional comments

*[If somewhat to very weak or neutral]*

Please provide a reasoning for your answer

There is a lack of evidence

There are discrepancies in existing evidence Existing evidence is of low quality

Other (please explain)

Third trimester: Vitamin B12 *(almost consensus on importance in Survey 1)*

Please rate your perception of the strength/quality of evidence that supplementation of **vitamin B12** during the **third trimester** for a person who is at **low risk** of complications improves pregnancy outcomes and/or foetal development

Very weak Weak

Somewhat

weak Neutral

Somewhat

strong Strong Very strong

Unsure/not applicable

Additional comments

*[If somewhat to very weak or neutral]*

Please provide a reasoning for your answer

There is a lack of evidence

There are discrepancies in existing evidence Existing evidence is of low quality

Other (please explain)

Third trimester: Vitamin D *(consensus on importance in Survey 1)*

Please rate your perception of the strength/quality of evidence that supplementation of **vitamin D** during the **third trimester** for a person who is at **low risk** of complications improves pregnancy outcomes and/or foetal development

Very weak Weak

Somewhat

weak Neutral

Somewhat

strong Strong Very strong

Unsure/not applicable

Additional comments

*[If somewhat to very weak or neutral]*

Please provide a reasoning for your answer

There is a lack of evidence

There are discrepancies in existing evidence Existing evidence is of low quality

Other (please explain)

Third trimester: Vitamin K *(no consensus on importance in Survey 1)*

Please rate your perception of the strength/quality of evidence that supplementation of **vitamin K** during the **third trimester** for a person who is at **low risk** of complications improves pregnancy outcomes and/or foetal development

Very weak Weak

Somewhat

weak Neutral

Somewhat

strong Strong Very strong

Unsure/not applicable

Additional comments

*[If somewhat to very weak or neutral]*

Please provide a reasoning for your answer

There is a lack of evidence

There are discrepancies in existing evidence Existing evidence is of low quality

Other (please explain)

Third trimester: Folic acid/folate *(no consensus on importance in Survey 1)*

Please rate your perception of the strength/quality of evidence that supplementation of **folic acid/folate** during the **third trimester** for a person who is at **low risk** of complications improves pregnancy outcomes and/or foetal development

Very weak Weak

Somewhat

weak Neutral

Somewhat

strong Strong Very strong

Unsure/not applicable

Additional comments

*[If somewhat to very weak or neutral]*

Please provide a reasoning for your answer

There is a lack of evidence

There are discrepancies in existing evidence Existing evidence is of low quality

Other (please explain)

Third trimester: Choline *(no consensus on importance in Survey 1)*

Please rate your perception of the strength/quality of evidence that supplementation of **choline** during the **third trimester** for a person who is at **low risk** of complications improves pregnancy outcomes and/or foetal development

Very weak Weak

Somewhat

weak Neutral

Somewhat

strong Strong Very strong

Unsure/not applicable

Additional comments

*[If somewhat to very weak or neutral]*

Please provide a reasoning for your answer

There is a lack of evidence

There are discrepancies in existing evidence Existing evidence is of low quality

Other (please explain)

Third trimester: Iodine *(almost consensus on importance in Survey 1)*

Please rate your perception of the strength/quality of evidence that supplementation of **iodine** during the **third trimester** for a person who is at **low risk** of complications improves pregnancy outcomes and/or foetal development

Very weak Weak

Somewhat

weak Neutral

Somewhat

strong Strong Very strong

Unsure/not applicable

Additional comments

*[If somewhat to very weak or neutral]*

Please provide a reasoning for your answer

There is a lack of evidence

There are discrepancies in existing evidence Existing evidence is of low quality

Other (please explain)

Third trimester: Magnesium *(no consensus on importance in Survey 1)*

Please rate your perception of the strength/quality of evidence that supplementation of **magnesium** during the **third trimester** for a person who is at **low risk** of complications improves pregnancy outcomes and/or foetal development

Very weak Weak

Somewhat

weak Neutral

Somewhat

strong Strong Very strong

Unsure/not applicable

Additional comments

*[If somewhat to very weak or neutral]*

Please provide a reasoning for your answer

There is a lack of evidence

There are discrepancies in existing evidence Existing evidence is of low quality

Other (please explain)

Third trimester: Calcium *(almost consensus on importance in Survey 1)*

Please rate your perception of the strength/quality of evidence that supplementation of **calcium** during the **third trimester** for a person who is at **low risk** of complications improves pregnancy outcomes and/or foetal development

Very weak Weak

Somewhat

weak Neutral

Somewhat

strong Strong Very strong

Unsure/not applicable

Additional comments

*[If somewhat to very weak or neutral]*

Please provide a reasoning for your answer

There is a lack of evidence

There are discrepancies in existing evidence Existing evidence is of low quality

Other (please explain)

Third trimester: Iron *(consensus on importance in Survey 1)*

Please rate your perception of the strength/quality of evidence that supplementation of **iron** during the **third trimester** for a person who is at **low risk** of complications improves pregnancy outcomes and/or foetal development

Very weak Weak

Somewhat

weak Neutral

Somewhat

strong Strong Very strong

Unsure/not applicable

Additional comments

*[If somewhat to very weak or neutral]*

Please provide a reasoning for your answer

There is a lack of evidence

There are discrepancies in existing evidence Existing evidence is of low quality

Other (please explain)

Third trimester: Iron *(consensus on importance in Survey 1)*

Please provide a reasoning for your answer

There is a lack of evidence

There are discrepancies in existing evidence Existing evidence is of low quality

Other (please explain)

Third trimester: Selenium *(no consensus on importance in Survey 1)*

Please rate your perception of the strength/quality of evidence that supplementation of **selenium** during the **third trimester** for a person who is at **low risk** of complications improves pregnancy outcomes and/or foetal development

Very weak Weak

Somewhat

weak Neutral

Somewhat

strong Strong Very strong

Unsure/not applicable

Additional comments

*[If somewhat to very weak or neutral]*

Please provide a reasoning for your answer

There is a lack of evidence

There are discrepancies in existing evidence Existing evidence is of low quality

Other (please explain)

Third trimester: Docosahexaenoic acid *(DHA; consensus on importance in Survey*

*1)*

Please rate your perception of the strength/quality of evidence that supplementation of **DHA** during the **third trimester** for a person who is at **low risk** of complications improves pregnancy outcomes and/or foetal development

Very weak Weak

Somewhat

weak Neutral

Somewhat

strong Strong Very strong

Unsure/not applicable

Additional comments

*[If somewhat to very weak or neutral]*

Please provide a reasoning for your answer

There is a lack of evidence

There are discrepancies in existing evidence Existing evidence is of low quality

Other (please explain)

Section 5 of 5: Lactation

For each of the following, please indicate how important dietary supplementation is during

**lactation** for a person who has experienced a **low risk** pregnancy

Slightly

Not at all

Not

not

Slightly

Extremely Unsure/not

important Important important important Neutral important Important important applicable

**Calcium** *(almost consensus in Survey 1:*

*74.19%*

*selected slightly to extremely important in Survey 1)*

**Iron**

*(almost consensus in Survey 1:*

*70.97%*

*selected slightly to extremely important in Survey 1)*

Additional comments

Lactation: Vitamin B1 *(no consensus on importance in Survey 1)*

Please rate your perception of the strength/quality of evidence that supplementation of **vitamin B1** during **lactation** for a person who has experienced a **low risk** pregnancy improves maternal health and/or infant development

Very weak Weak

Somewhat

weak Neutral

Somewhat

strong Strong Very strong

Unsure/not applicable

Additional comments

*[If somewhat to very weak or neutral]*

Please provide a reasoning for your answer

There is a lack of evidence

There are discrepancies in existing evidence Existing evidence is of low quality

Other (please explain)

Lactation: Vitamin B2 *(no consensus on importance in Survey 1)*

Please rate your perception of the strength/quality of evidence that supplementation of **vitamin B2** during **lactation** for a person who has experienced a **low risk** pregnancy improves maternal health and/or infant development

Very weak Weak

Somewhat

weak Neutral

Somewhat

strong Strong Very strong

Unsure/not applicable

Additional comments

*[If somewhat to very weak or neutral]*

Please provide a reasoning for your answer

There is a lack of evidence

There are discrepancies in existing evidence Existing evidence is of low quality

Other (please explain)

Lactation: Vitamin B6 *(no consensus on importance in Survey 1)*

Please rate your perception of the strength/quality of evidence that supplementation of **vitamin B6** during **lactation** for a person who has experienced a **low risk** pregnancy improves maternal health and/or infant development

Very weak Weak

Somewhat

weak Neutral

Somewhat

strong Strong Very strong

Unsure/not applicable

Additional comments

*[If somewhat to very weak or neutral]*

Please provide a reasoning for your answer

There is a lack of evidence

There are discrepancies in existing evidence Existing evidence is of low quality

Other (please explain)

Lactation: Vitamin B12 *(no consensus on importance in Survey 1)*

Please rate your perception of the strength/quality of evidence that supplementation of **vitamin B12** during **lactation** for a person who has experienced a **low risk** pregnancy improves maternal health and/or infant development

Very weak Weak

Somewhat

weak Neutral

Somewhat

strong Strong Very strong

Unsure/not applicable

Additional comments

*[If somewhat to very weak or neutral]*

Please provide a reasoning for your answer

There is a lack of evidence

There are discrepancies in existing evidence Existing evidence is of low quality

Other (please explain)

Lactation: Vitamin D *(consensus on importance in Survey 1)*

Please rate your perception of the strength/quality of evidence that supplementation of **vitamin D** during **lactation** for a person who has experienced a **low risk** pregnancy improves maternal health and/or infant development

Very weak Weak

Somewhat

weak Neutral

Somewhat

strong Strong Very strong

Unsure/not applicable

Additional comments

*[If somewhat to very weak or neutral]*

Please provide a reasoning for your answer

There is a lack of evidence

There are discrepancies in existing evidence Existing evidence is of low quality

Other (please explain)

Lactation: Vitamin K *(no consensus on importance in Survey 1)*

Please rate your perception of the strength/quality of evidence that supplementation of **vitamin K** during **lactation** for a person who has experienced a **low risk** pregnancy improves maternal health and/or infant development

Very weak Weak

Somewhat

weak Neutral

Somewhat

strong Strong Very strong

Unsure/not applicable

Additional comments

*[If somewhat to very weak or neutral]*

Please provide a reasoning for your answer

There is a lack of evidence

There are discrepancies in existing evidence Existing evidence is of low quality

Other (please explain)

Lactation: Folic acid/folate *(no consensus on importance in Survey 1)*

Please rate your perception of the strength/quality of evidence that supplementation of **folic acid/folate** during **lactation** for a person who has experienced a **low risk** pregnancy improves maternal health and/or infant development

Very weak Weak

Somewhat

weak Neutral

Somewhat

strong Strong Very strong

Unsure/not applicable

Additional comments

*[If somewhat to very weak or neutral]*

Please provide a reasoning for your answer

There is a lack of evidence

There are discrepancies in existing evidence Existing evidence is of low quality

Other (please explain)

Lactation: Choline *(no consensus on importance in Survey 1)*

Please rate your perception of the strength/quality of evidence that supplementation of **choline** during **lactation** for a person who has experienced a **low risk** pregnancy improves maternal health and/or infant development

Very weak Weak

Somewhat

weak Neutral

Somewhat

strong Strong Very strong

Unsure/not applicable

Additional comments

*[If somewhat to very weak or neutral]*

Please provide a reasoning for your answer

There is a lack of evidence

There are discrepancies in existing evidence Existing evidence is of low quality

Other (please explain)

Lactation: Iodine *(no consensus on importance in Survey 1)*

Please rate your perception of the strength/quality of evidence that supplementation of **iodine** during **lactation** for a person who has experienced a **low risk** pregnancy improves maternal health and/or infant development

Very weak Weak

Somewhat

weak Neutral

Somewhat

strong Strong Very strong

Unsure/not applicable

Additional comments

*[If somewhat to very weak or neutral]*

Please provide a reasoning for your answer

There is a lack of evidence

There are discrepancies in existing evidence Existing evidence is of low quality

Other (please explain)

Lactation: Magnesium *(no consensus on importance in Survey 1)*

lease rate your perception of the strength/quality of evidence that supplementation of **magnesium** during **lactation** for a person who has experienced a **low risk** pregnancy improves maternal health and/or infant development

Very weak Weak

Somewhat

weak Neutral

Somewhat

strong Strong Very strong

Unsure/not applicable

Additional comments

*[If somewhat to very weak or neutral]*

Please provide a reasoning for your answer

There is a lack of evidence

There are discrepancies in existing evidence Existing evidence is of low quality

Other (please explain)

Lactation: Calcium *(almost consensus on importance in Survey 1)*

Please rate your perception of the strength/quality of evidence that supplementation of **calcium** during **lactation** for a person who has experienced a **low risk** pregnancy improves maternal health and/or infant development

Very weak Weak

Somewhat

weak Neutral

Somewhat

strong Strong Very strong

Unsure/not applicable

Additional comments

*[If somewhat to very weak or neutral]*

Please provide a reasoning for your answer

There is a lack of evidence

There are discrepancies in existing evidence Existing evidence is of low quality

Other (please explain)

Lactation: Iron *(almost consensus on importance in Survey 1)*

Please rate your perception of the strength/quality of evidence that supplementation of **iron** during **lactation** for a person who has experienced a **low risk** pregnancy improves maternal health and/or infant development

Very weak Weak

Somewhat

weak Neutral

Somewhat

strong Strong Very strong

Unsure/not applicable

Additional comments

*[If somewhat to very weak or neutral]*

Please provide a reasoning for your answer

There is a lack of evidence

There are discrepancies in existing evidence Existing evidence is of low quality

Other (please explain)

Lactation: Selenium *(no consensus on importance in Survey 1)*

Please rate your perception of the strength/quality of evidence that supplementation of **selenium** during **lactation** for a person who has experienced a **low risk** pregnancy improves maternal health and/or infant development

Very weak Weak

Somewhat

weak Neutral

Somewhat

strong Strong Very strong

Unsure/not applicable

Additional comments

*[If somewhat to very weak or neutral]*

Please provide a reasoning for your answer

There is a lack of evidence

There are discrepancies in existing evidence Existing evidence is of low quality

Other (please explain)

Lactation: Docosahexaenoic acid *(DHA; consensus on importance in Survey 1)*

Please rate your perception of the strength/quality of evidence that supplementation of **DHA** during **lactation** for a person who has experienced a **low risk** pregnancy improves maternal health and/or infant development

Very weak Weak

Somewhat

weak Neutral

Somewhat

strong Strong Very strong

Unsure/not applicable

Additional comments

*[If somewhat to very weak or neutral]*

Please provide a reasoning for your answer

There is a lack of evidence

There are discrepancies in existing evidence Existing evidence is of low quality

Other (please explain)
